# Deuteration of heptamethine cyanine dyes enhances their emission efficacy†

**DOI:** 10.1039/d3cc05153f

**Published:** 2023-12-18

**Authors:** Hana Janeková, Hannah C. Friedman, Marina Russo, Mergime Zyberaj, Tasnim Ahmed, Ash Sueh Hua, Anthony V. Sica, Justin R. Caram, Peter Štacko

**Affiliations:** a Department of Chemistry, University of Zurich Wintherthurerstrasse 190 Zurich 8057 Switzerland peter.stacko@uzh.ch; b Department of Chemistry and Biochemistry, University of California, Los Angeles 607 Charles E. Young Drive Los Angeles CA 90095-1569 USA jcaram@chem.ucla.edu

## Abstract

The design of bright short-wave infrared fluorophores remains a grand challenge. Here we investigate the impact of deuteration on the properties in a series of heptamethine dyes, the absorption of which spans near-infrared and SWIR regions. We demonstrate that it is a generally applicable strategy that leads to enhanced quantum yields of fluorescence, longer-lived singlet excited states and suppressed rates of non-radiative deactivation processes.

## Abstract 2

We demonstrate that deuteration is a generally applicable strategy that leads to enhanced quantum yields of fluorescence, longer-lived singlet excited states and suppressed rates of non-radiative deactivation processes.

## Graphical abstract

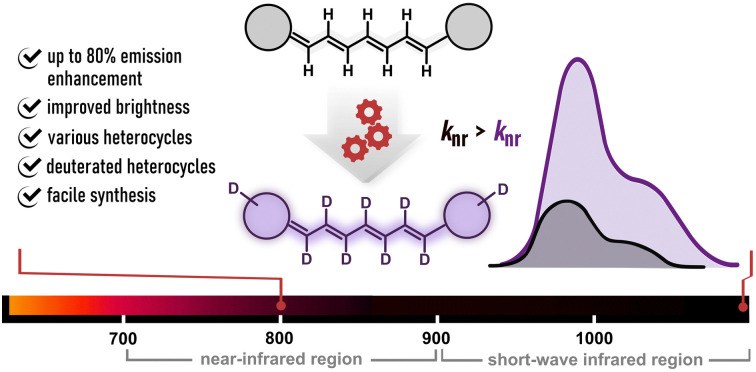

The move towards short-wave infrared (SWIR; 900–2000 nm) region has recently emerged as a complementary approach to enhance bioimaging techniques with unrivaled spatiotemporal resolution.^1^ The feasibility of SWIR imaging was demonstrated *in vivo* using carbon nanotubes,^2^ quantum dots,^3^ rare-earth nanomaterials^4^ and small molecules.^5^ The full potential of SWIR imaging in terms of deep penetration, high spatial resolution, multicolor imaging and fast acquisition rates was showcased using quantum dots.^6^ Their high emission efficacy enabled heartbeat and breathing rate quantification in awake animals and construction of a brain vasculature map. SWIR imaging was recently employed to realize excitation multiplexing in awake animals with video-rate *in vivo* imaging.^7,8^

However, the full potential of bioimaging in SWIR region is restricted by critically underperforming organic small-molecules-based fluorescent probes with fluorescence quantum yields (*Φ*_F_) generally below 1%. The existing probes are usually based on donor–acceptor–donor motif or, more commonly, cyanine scaffold (Cy7).^5,9–14^ We and Schnermann have shown that the low *Φ*_F_ of Cy7 cyanines are linked to fast non-radiative deactivation processes in the singlet excited state instead of *E*–*Z* photoisomerization.^15,16^ The rate of these processes increases exponentially with the decreasing HOMO–LUMO energy gap (*i.e.* red-shifted absorption maximum), also known as the “energy gap law”.^17^ As a result, the design of bright and efficient organic SWIR fluorophores remains a grand challenge. Besides SWIR applications, extending the lifetimes is crucial also in the context of other applications that rely on chemistry occurring from the excited state.^18–24^

Some of us recently conceptualized the deuteration of cyanine scaffold as a potentially productive avenue to highly emissive SWIR fluorophores (Fig. 1) *via* suppression of non-radiative deactivation rates, owed to stiffer and less energetic C–D stretching vibrations (*ṽ* ∼ 2200 cm^−1^) compared to those of C–H (*ṽ* ∼ 3100 cm^−1^).^25^ Recently, deuteration of *N*-methyl substituents in rhodamines resulted in a significant increase of their *Φ*_F_.^26^ Besides affecting emission properties, deuteration has been shown to increase the thermal stability of indocyanine green (ICG) and extend its shelf life harnessing the kinetic isotope effect.^27^ Herein, we investigate the deuteration along the entire central chain in a series of near-infrared- and SWIR-emitting Cy7 dyes bearing different terminal heterocycles as a general strategy to enhance their emissive properties.

**Fig. 1:**
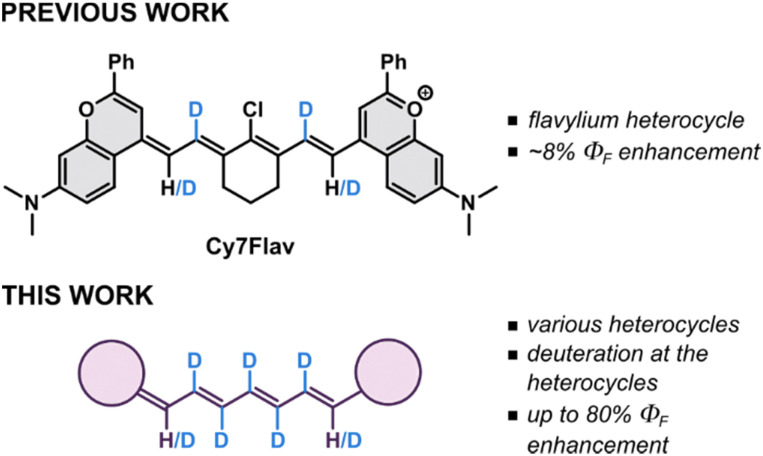
Comparison of the state-of-the-art with this work.

Cyanines 1–4D were synthesized from pyridine-d_5_ as a cheap and readily available source of deuterium atoms (Scheme 1). Pyridine-d_5_ was transformed to the corresponding Zincke salt 5,^28^ and subsequently ring-opened using aniline in D_2_O/CD_3_OD to provide intermediate 6D in a good overall yield. The intermediate 6D was activated by *in situ* acetylation using Ac_2_O and condensed with the corresponding heterocycles 9–13 to provide deuterated Cy7 1–4D as mixtures of d_7_ : d_6_ : d_5_ in variable ratios determined from isotope pattern of HRMS. In case of 1H–D, intensive degassing was necessary to prevent oxidation of the flavylium heterocycle 10 as observed previously by Sletten and co-workers.^29^ The deuteria contained in the heterocycle 9 required for 1DD were introduced by methylation of the starting 3-aminophenol with CD_3_I (see ESI†). The protonated derivatives 1–3H were prepared in analogous fashion starting from the commercial 6H.

**Scheme 1:**
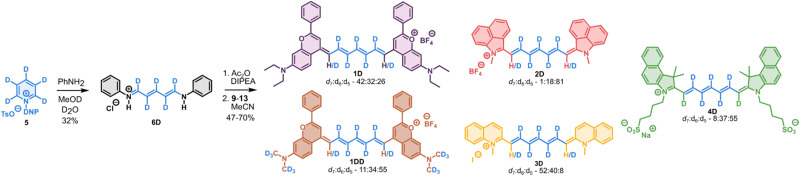
Synthesis of deuterated cyanines 1–4D and 1DD. Protonated analogues 1H–3H were prepared in analogous fashion using non-deuterated solvents.

Photophysical and photochemical properties of the synthesized fluorophores are summarized in Table 1. The measurements were performed in dichloromethane (DCM) to decouple the investigations from aggregation phenomena, and to facilitate straightforward comparison with the values reported in the literature. Deuteration of the heptamethine chain showed negligible effect on the absorption properties of 1–4D and led only to a minor shift of their absorption maxima (Fig. 2A and B). The derivatives 1H, 1D, 1DD and 2H–D possess absorption maxima at ∼990 nm and emission maxima above ∼1020 nm, consistent with analogous derivatives.^12^ Consistent with the literature,^16,27^3D and 4D display more blue shifted absorption maxima at 832 and 782 nm, respectively, and ∼30 and ∼50 nm Stokes shifts, respectively. All the derivatives show large molar absorption coefficients typical for Cy7 dyes,^7,16,30^ and small Stokes shifts (25–52 nm) which did not display trend nor were they significantly altered by deuteration.

**Table 1:** Photophysical properties of the studied Cy7 fluorophores

	*λ*_abs_ a/nm	*λ*_em_ a/nm	*ε* a b	*d* c/%	*Φ*_F_ ×10^2^^a^^d^	*χ* e/%	*εΦ*_F_
1H	992	1029	280 800	—	1.33 ± 0.08	—	3730
1D	990	1022	268 700	14	1.56 ± 0.09	17 ± 0.9	4190
1DD	982	1015	296 300	47	2.39 + 0.02	80 ± 0.8	7080
2H	987	1014	236 900	—	0.33 ± 0.026	—	780
2D	986	1011	219 900	21	0.36 ± 0.025	9.9 ± 0.9	790
3H	832	860	224 300	—	0.06 ± 0.004	—	130
3D	832	860	237 900	26	0.05 ± 0.004	−17 ± 1.0	120
4H	784^f^	830	226 000^f^	—	11.5 ± 0.6	—	26 000
4D	782^f^	834	169 700^f^	12	13.7 ± 0.6	19 ± 0.7	23 100

a Determined in DCM with 0.4% of DMSO.

b The molar absorption coefficient, *ε*_max_/mol^−1^ dm^3^ cm^−1^.

c Overall degree of the deuteration.

d Quantum yield of fluorescence relative to the reference. Average and standard deviations of the mean are given.

e Enhancement of the quantum yield of fluorescence by deuteration defined as *χ =* 100 × (*Φ*_D_/*Φ*_H_ −1).

f Determined in MeOH with 0.4% DMSO due to its aggregation in DCM.

**Fig. 2:**
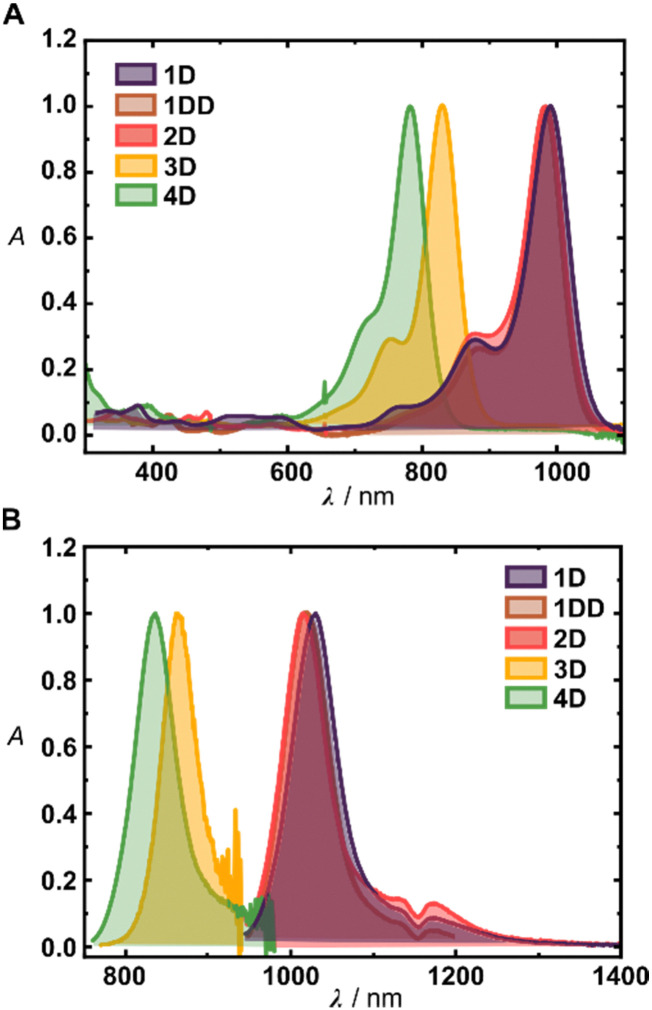
(A) UV-Vis absorption spectra of 1D–4D in DCM. (B) Emission spectra of 1D–4D in DCM.

The emerging importance of cyanines as SWIR fluorophores motivated us to investigate the effect of deuteration on their quantum yields of fluorescence (*Φ*_F_). In general, the deuterated analogues demonstrated a clear enhancement of *Φ*_F_ (*χ* = 100 × (*Φ*_D_/*Φ*_H_ −1), Fig. 3C), whereas the protonated parent compounds showed *Φ*_F_ values consistent with the literature.^7,16^ Specifically, cyanines 1D, 1DD, 2D and 4D display *χ* of 17%, 80%, 10%, and 19%, respectively, whereas the quinolinium derivative 3D exhibits a relative decrease of 17%. We attribute the origin of this outlier to its very weak emission (*Φ*_F_ < 0.06%) that is at the very limit of the InGaAs detector, introducing a large experimental error. We determined *χ* for 4H–D determined also by an absolute method using an integrating sphere, and the value of *χ* (20%) was in an excellent agreement with the relative method (19%). The observed *χ* for 4H–D was also comparable to the results of Smith and co-workers obtained in DMSO.^27^1DD was prepared to evaluate if *χ* increases with the degree of deuteration. Indeed, 1DD incorporating higher deuterium content experienced significantly higher *χ* compared to that of 1D. With the total brightness of 7080 M^−1^ cm^−1^, 1DD is 4.8-fold brighter than the previously best performing flavylium-based Cy7, and substantially brighter than the structurally related chromenylium analogue (∼65%).^8^

**Fig. 3:**
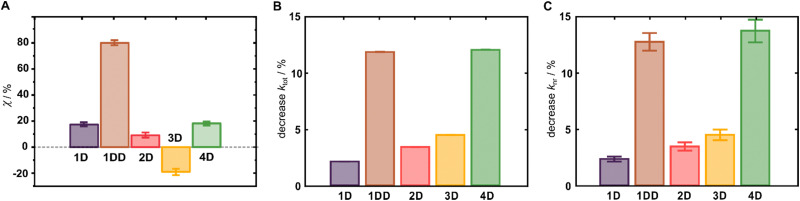
(A) Enhancement (*χ*) of *Φ*_F_ in 1D–4D induced by deuteration of the Cy7 scaffold. (B) Percent difference for *k*_tot_. (C) Percent difference for *k*_nr_.

Excluding 1DD, no clear correlation of *χ* with the HOMO–LUMO energy gap (*i.e.* the absorption maxima) was observed in our series as would be expected from the energy gap law. Intrigued by this, we decided to consider other potential deactivation pathways since the efficacy of the fluorophore is limited by the worst deactivation pathway. *Φ*_F_ of 1D is not improved in more viscous DMSO (*η* = 2.0 cP) compared to DCM (*η* = 0.5 cP), suggesting that rotation of the phenyl substituent at the flavylium core is also not significantly involved in the deactivation (Fig. S52, ESI†). The excellent work of Sletten also provides important insights in this regard.^8^ They showed that, unlike in other chromophores, introduction of julolidine to restrict the motion around the C–N bond does not improve *Φ*_F_, suggesting twisted intramolecular charge transfer (TICT) is not a major deactivation pathway. At the same time, introduction of a *tert*-Bu substituent in the 2-position of the heterocycles improved *Φ*_F_ by 2.8-fold. Therefore, we conclude that different C–H vibrations, *e.g.* located on the terminal aromatic rings, are likely significantly more efficient at dissipating the energy of the excited state in different Cy7 scaffolds. This view is consistent with the previous work of Hirata on perdeuterated aromatic amines.^31^ In this context, deuteration of the heterocycles in combination with their modification, especially in the 2-position, may be a path forward to bright SWIR fluorophores.

Notably, we also observed a large, 3-fold increase of *Φ*_F_ in 1H compared to its analogue containing a cyclohexenyl ring embedded in the central chain.^8,25^ This is contrary to the popular notion in the literature that the ring increases *Φ*_F_ of Cy7 dyes through rigidification and suppression of the potential *E*–*Z* photoisomerization. While this structural feature provides benefits from the synthetic point of view, we have recently shown that it provides no improvements of photophysical properties.^16^ Little improvement of *Φ*_F_*via* complete rigidification of the Cy7 scaffold reported by Schnermann and co-workers further corroborates this notion.^15^ Nevertheless, the explicit negative influence of the ring on *Φ*_F_ was unexpected. We speculated whether the large *Φ*_F_ increase is due to elimination of the deactivation pathways conferred by the additional C–H bonds in the ring, decreased *Φ*_ISC_ due to eliminating potential heavy atom effects of Cl atom. Alternatively, the increase could come from 1H–DD being blue-shifted compared to the flavylium derivatives in the literature (∼40 nm) due to absence of electron accepting Cl atom. While the latter complicates the direct comparison, independent energy gap parameter as described by Caram and coworkers which decouples the phenomena,^25^ shows a value of 0.5 which is indicative of an increase quantum yield compared to the cyclohexyl ring analogue beyond that of the blue shift.

To corroborate observations from stead-state emission spectroscopy, we utilized time-resolved emission spectroscopy to gain additional insight into the effect of deuteration on the excited state lifetimes and the rates of non-radiative deactivation processes. We observed a statistically significant increase of lifetimes upon deuteration in the entire series of Cy7 dyes (Fig. 3B and C, Table 2). An overall decrease in non-radiative rates upon deuteration was also observed, with 1DD exhibiting improvement that is statistically significant *via t*-test (*p* < 0.05), which indicates that deuteration is eliminating deactivation pathways in all dyes. Additionally, all these dyes are at least 40 nm blue shifted from the parent cyclohexyl Cy7Flav analogue, which means thatthe non-radiative energy gap law for internal conversion would be less impactful to the change in quantum yield. On the other hand, 1DD may show decrease in non-radiative rate because TICT of the amine may dominate the rate in the linear dyes more because of this blue shift. When analyzing a decrease in rate compared to the rate for the protonated analogue, we observe smaller changes than those found in the quantum yield analysis. Using this analysis on the previously reported Cy7Flav derivatives,^25^ we observe similar decrease values for non-radiative rate for the partially deuterated compounds (4.1 ± 0.01% and 5.5 ± 0.02% for deuteration degrees of 2 and 2.12 in Cy7Flav scaffold, respectively). More interestingly, the rate decrease for the radiative rate is −1.11 ± 0.06% and −2.2 ± 0.2%, respectively, which is much lower change than the 1D change in the radiative rate. Though much more scanning of the synthetic space must be considered, this may be indicative of deuteration impacting the transition dipole moment in certain derivatives.

**Table 2:** Time resolved fluorescence lifetimes and rates of Cy7 fluorophores^a^

	*τ*/ps	*k*_tot_/10^9^ s^−1^	*h* b/%	*k*_r_/10^6^ s^−1^	*h* b/%	*k*_nr_/10^9^ s^−1^	*h* b/%
1H	138.5 ± 0.1	7.22 ± 0.01		95 ± 6	—	7.1 ± 0.4	—
1D	141.5 ± 0.4	7.06 ± 0.02	2.10 ± 0.01	110 ± 6	−15 ± 1	7.0 ± 0.4	2.2 ± 0.2
1DD	158.7 ± 1.0	6.2 ± 0.05	11.74 ± 0.02	142 ± 1	−48 ± 3	6.23 ± 0.06	12.5 ± 8
2H	67.5 ± 0.2	14.81 ± 0.05		49 ± 5	—	15 ± 1	—
2D	69.9 ± 0.3	14.31 ± 0.05	3.38 ± 0.02	52 ± 4	−5.4 ± 0.5	14 ± 1	3.4 ± 0.4
3H	80.69 ± 0.09	12.39 ± 0.01		7. ± 0.5	—	12 ± 1	—
3D	84.44 ± 0.09	11.84 ± 0.01	4.43 ± 0.01	5.9 ± 0.5	20 ± 2	11.8 ± 0.9	4.43 ± 0.5
4H	481.2 ± 0.3	2.076 ± 0.001		239 ± 10	—	1.8 ± 0.1	—
4D	547 ± 0.3	1.828 ± 0.001	11.93 ± 0.01	239 ± 16	−0.32 ± 0.03	1.6 ± 0.1	14 ± 1

a Determined in pure DCM.

b Percent of the rate of decrease by deuteration defined as *h =* 100 × (1-*k*_D_/*k*_H_), *k*_r_ = *Φ*_F_ × *k*_tot_, *k*_nr_ = *k*_tot_-*k*_r_.

In conclusion, we demonstrate that deuteration is a valuable strategy applicable across the family of SWIR-absorbing Cy7 fluorophores to increase their emission efficacy and suppress the competing non-radiative deactivation pathways. We believe that the valuable lessons learned herein will guide the rational design of SWIR fluorophores and spur the investigations to identify bond vibrations which represent the greatest offenders in this context, or inspire alternative approaches to suppress these non-productive pathways, *e.g. via* perfluorination of the central polymethine chain or the appending heterocycles (*ṽ* of C–F ∼1200 cm^−1^).

We acknowledge Swiss National Science Foundation (P.Š/PZ00P2_193425), the Department of Chemistry, University of Zurich (Legerlotz Stiftung, UZH Candoc), and especially the Prof. Hans E. Schmid Stiftung for funding this research project. We would like to thank Prof. Cristina Nevado, Prof. Karl Gademann and Prof. Michal Juríček (University of Zurich) for the generous support of our researchJustin, Hannah, Tasnim, Ash, and Anthony would like to acknowledge support from the U.S. National Science Foundation (CHE-2204263, CHE-1945572), and the Cottrell foundation.

## Conflicts of interest

There are no conflicts to declare.

## Supplementary Material

CC-060-D3CC05153F-s001
