# Supplementary material for: Deuteration of heptamethine cyanine dyes enhances their emission efficacy
Source: Chem Commun (Camb). 2023 Dec 18;60(8):1000–3. doi: 10.1039/d3cc05153f (PMC10805072; doi:10.1039/d3cc05153f)
Supplement: CC-060-D3CC05153F-s001 [file CC-060-D3CC05153F-s001.pdf]

Electronic Supplementary Material (ESI) for ChemComm.
This journal is © The Royal Society of Chemistry 2023

Supporting information

Deuteration of Heptamethne Cyanine Dyes Enhances Their Emission Efficacy

Hana Janeková,[1] Hannah C. Friedman,[2] Marina Russo,[1] Mergime Zyberaj,[1] Tasnim Ahmed,[2] Ash Sueh Hua,[2] Anthony V. Sica,[2] Justin R. Caram,[2,*] Peter Štacko[1,*]

[1] Department of Chemistry, University of Zurich, Wintherthurerstrasse 190, 8057 Zurich, Switzerland
[2] Department of Chemistry and Biochemistry, University of California, Los Angeles, 607 Charles E. Young Drive, Los Angeles, CA 90095-1569, USA

Table of Contents

Materials and Methods 2
Synthesis 6
NMR Spectroscopy 12
HRMS Spectroscopy 29
UV-Vis and Emission Spectroscopy 31
Deuterium-Exchange Experiment 37
Additional Experiments 38
References 39

Materials and Methods

Reagents and solvents of the highest purity available were used as purchased, or they were purified/dried using standard methods when necessary. The intermediates were synthesized according to the published procedures (**10**[1], **11**[2], **12**[3], **13**[4,5]) or purchased (**4H**) from standard suppliers (Merck, TCI, Across Organics, etc.).

Flash column chromatography was performed using silica gel (230−400 mesh). ^{1}H NMR spectra were recorded on 400 or 500 MHz spectrometers; ^{13}C NMR spectra were obtained on 125 MHz instruments in $CDCl_3$, CD_3OD, and d_6-DMSO. ^{19}F NMR were obtained on 376 MHz or 470 MHz instruments. ^{1}H chemical shifts are reported in ppm relative to $CDCl_3$ (δ = 7.26 ppm), CD_3OD (δ = 3.31 ppm) and d_6-DMSO (δ = 2.50 ppm) as an internal reference. ^{13}C chemical shifts are reported in ppm with $CDCl_3$ (δ = 77.67 ppm), CD_3OD (δ = 49.30 ppm) and d_6-DMSO (δ = 39.52 ppm) as internal references. ^{2}H NMR spectra were measured using non-deuterated solvents with addition of 5 μL of respective deuterated solvent to allow locking of a sample. Deuterated solvents were kept under nitrogen atmosphere. HRMS of the synthesized compounds was obtained using a triple quadrupole electrospray ionization mass spectrometer in a positive or negative mode coupled with direct inlet. The relative ratio of d_5:d_6:d_7 for the final cyanines **1**–**4D** was calculated from isotope pattern of the molecular peak detected by HRMS.

Steady-State Absorption and Emission Spectroscopy

Absorption spectra and molar absorption coefficients were obtained on a UV-vis spectrometer with matched 1.0 cm quartz cells in CH_2Cl_2 (**1-3**) or MeOH (**4**), with constant amount 0.4% of DMSO. Fluorescence spectra were measured using a fluorescence spectrometer in a 1.0 cm quartz fluorescence cuvette at 20 °C in CH_2Cl_2. The sample concentrations were adjusted to keep the absorbance below ~0.2 at the corresponding excitation wavelength. Each sample was measured five times, and the spectra were averaged. Emission spectra were normalized and corrected by the photomultiplier sensitivity function using correction files supplied by the manufacturer.

Quantum yields of fluorescence were measured in CH_2Cl_2 against the reference using the relative method. For compounds **1** and **2**, IR-1061 was used as a reference standard (Φ_F = 0.32%[6] in CH_2Cl_2). For compounds **3** and **4** dye **4H** (ICG) was used as a reference standard (Φ_F = 12%[7] in methanol), and the calculations were done using eq. bellow for correction of refractive indexes.

$$\Phi_S = \Phi_R \left(\frac{\text{slope}_S}{\text{slope}_R}\right)\left(\frac{n_S}{n_R}\right)^2$$

The subscripts $_S$ and $_R$ refer to the sample and reference, respectively. Φ_R is the known quantum yield of the reference standard, slope is the slope of integrated fluorescence spectrum intensity plotted against absorbance A of the solution at the excitation wavelength (λ_{ex}), and n is the refractive index of the solvent, using values n_S = 1.4244 for CH_2Cl_2 and n_R = 1.4793 for methanol. Each compound was measured using 15 independent solutions with absorbance in a range 0.02-0.2 at a respective excitation wavelength and compared to reference sample measured under the same settings using 10–15 independent solutions with absorbance in a range 0.02-0.2. Error calculation of the quantum yield was propagated from the error in slope of both the reference and the unknown.

Stability of 1 DD In the Dark and Under Irradiation

Compounds **1H** and **1DD** were dissolved in CH_2Cl_2 (A=1 at 988 nm) and irradiated with LEDs at 820 nm (~25 mW/cm^2) and the progress of the irradiation was monitored at periodic intervals (10 min for irradiated, 30 min for the experiment in the dark) by UV−vis spectrometry. The stability of **1H** and **1DD** in the dark was recorded using the same procedure with exclusion of the irradiation source.

Time-Resolved Emission Spectroscopy

Time-correlated single photon counting was employed for the time resolved emission measurement of **1-4D** in solution (DCM). A 780 nm pulsed diode laser (PicoQuant LDH-P-C-780) with a repetition rate of 80 MHz was used as the excitation source. An epifluorescent configuration was used to collect emission with a 650 long pass dichroic mirror (Thorlabs DMLP650R). The laser was filtered using 830 nm longpass filter (Newport Optics 10CGA-830). The emission was fiber coupled into reflective collimators(Thorlabs RC12FC-P01) and detected using superconducting nanowire single-photon detectors (Quantum Opus Opus One). A timing module recorded all photon events (Picoquant HydraHarp 400) in time-tagged time-resolved mode.

Given the short lifetimes of these dye, lifetimes were fit using a single exponential with an offset from the peak maximum based on the Gaussian instrument response function (46 ps for all but 1DD and 17 ps for 1DD) (Figure S1). A overlay of 4H and 4D is provided to show the

difference in lifetimes (Figure S2).

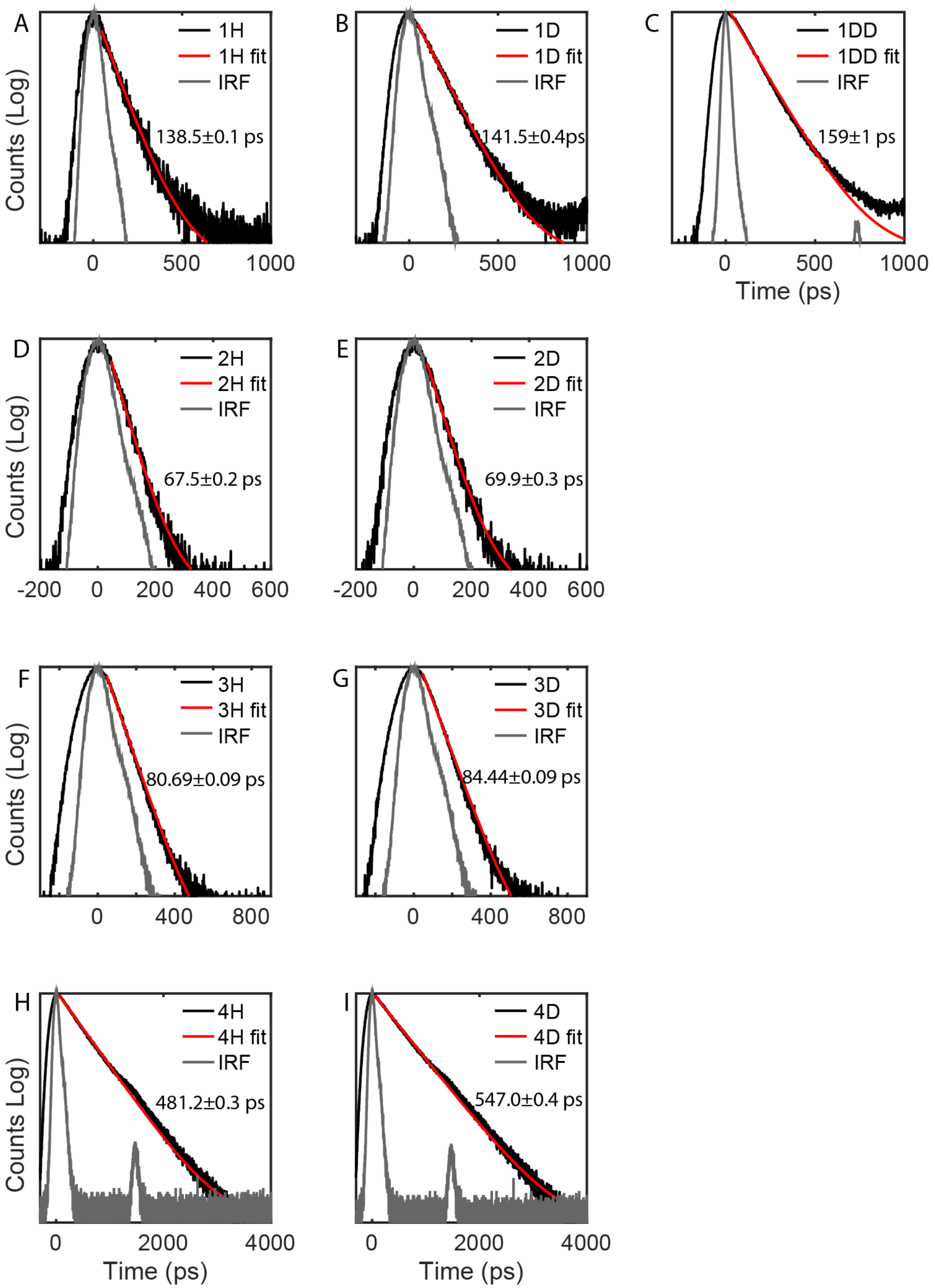

Figure S1: Time correlated photon counts of IRF (grey) and dye (black) and fits (red) of **1H** (A), **1D** (B), **1DD** (C) **2H** (D), **2H** (E), **3H** (F), **3D** (G), **4H** (H), **and 4D** (I).

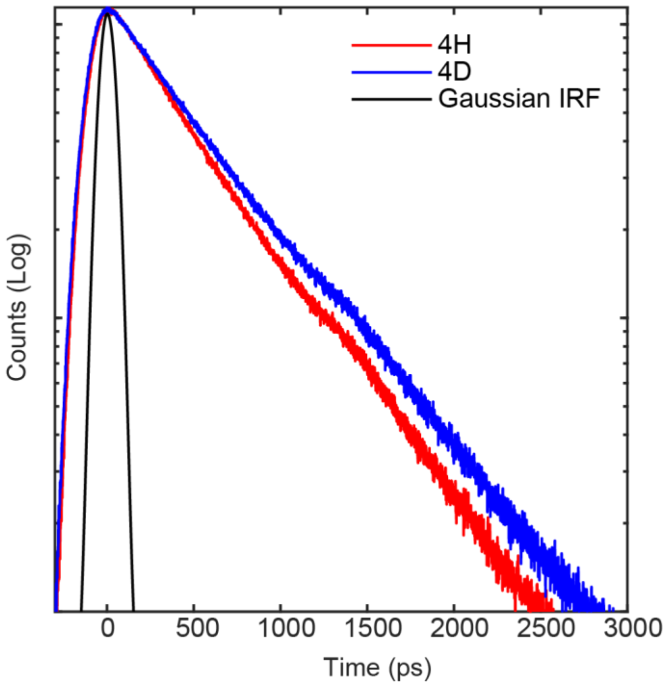

Figure S2: Comparison of the TCSPC lifetime trace of dye **4H** and **4D** to show that deuteration increases lifetime.

Table S2. Values of R-squared for the fits in Figure S1

TSCPC trace	R-squared
IRF (single gaussian)	0.9901
IRF **1DD** (single gaussian)	0.9928
**1H**	0.9818
**1D**	0.9976
**1DD**	0.9981
**2H**	0.9753
**2D**	0.9745
**3H**	0.9974
**3D**	0.9976
**4H**	0.9993
**4D**	0.9993

Synthesis of the Intermediates and Cyanines 1-4D and 1-3H.

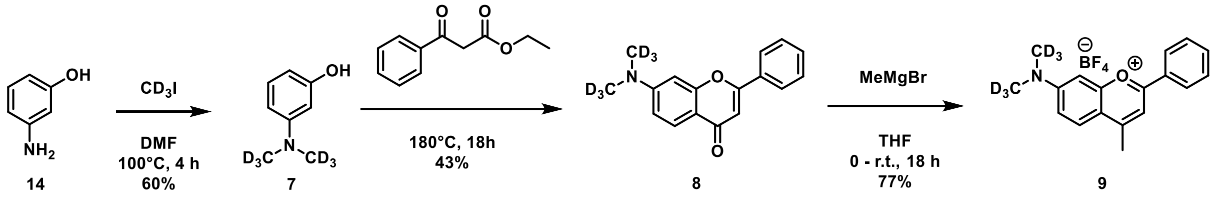

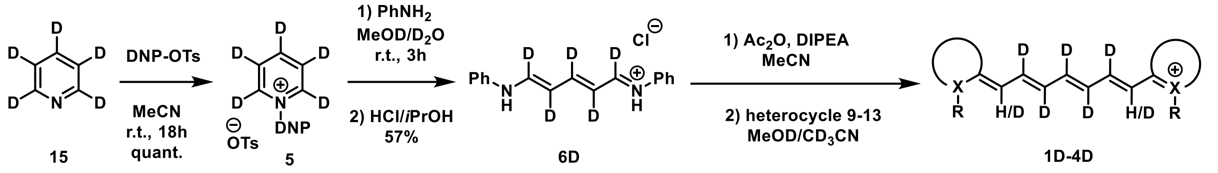

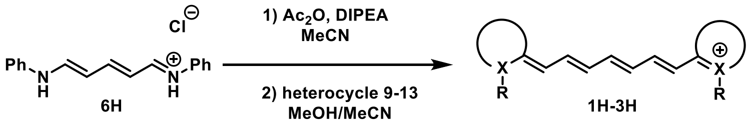

Structures of the studied molecules and the terminal heterocycles:

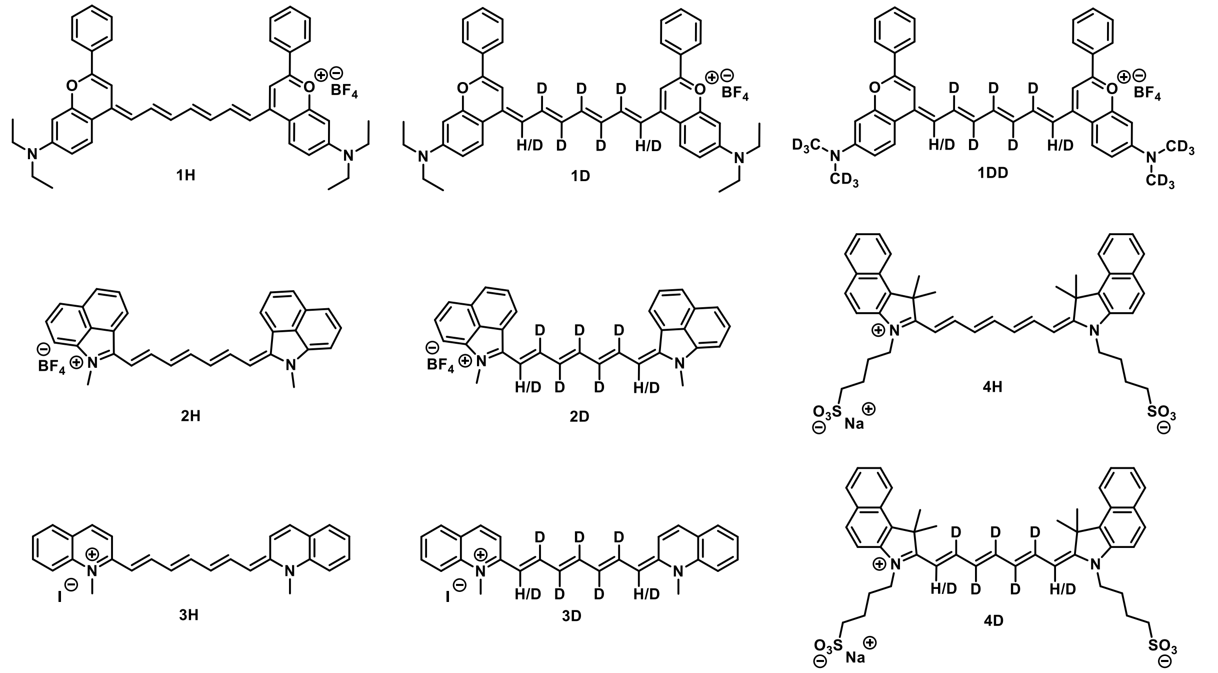

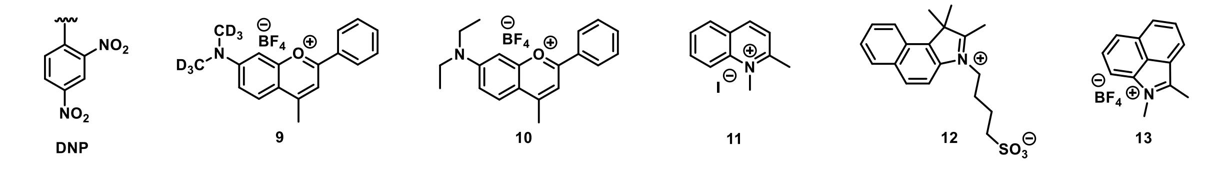

4-(Bis(methyl-*d*$_3$)amino)phenol (7).

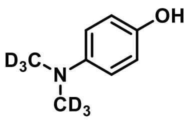

A mixture of 4-aminophenol (**14**) (3.01 g, 27.0 mmol) and CD_3I (2.7 mL, 43.2 mmol) was heated in DMF (20 mL) in the presence of K_2CO_3 (5.97 g, 43.2 mmol) in a pressure tube at 100 °C for 5 h. The reaction mixture was then cooled down to room temperature and stirred for 12 h. The precipitate was filtered off and the filtrate was concentrated under reduced pressure. The solid residue after filtration was dissolved in water (50 mL), neutralized with conc. HCl (32 %) and extracted with EtOAc (3×50 mL). The evaporated filtrate was dissolved in EtOAc and extracted with H_2O (3×75 mL). Organic part was combined with the extraction above, dried with $MgSO_4$, filtered, and the solvent was removed under reduced pressure. The resulting oily mixture was purified by column chromatography (SiO_2, pentane/EtOAc gradient 6:1 – 5:1) providing phenol **7**. Yield: 2.37 g (61%). White solid. M.p. 97.4–98.1°C. ^{1}H NMR (500 MHz, $CDCl_3$) δ (ppm) 7.1 (dd, J_1 = 8.1, J_2 = 8.1 Hz, 1H), 6.41 – 6.18 (m, 3H), 5.10 (brs, 1H).^{2}H NMR (92 MHz, $CDCl_3$) δ (ppm) 2.89 (s, 6H). ^{13}C NMR (126 MHz, $CDCl_3$) δ (ppm) 156.8, 151.7, 130.2, 105.9, 104.8, 100.4, 40.3 (sep, J = 22 Hz). ^{13}C{^{2}H} NMR (151 MHz, $CDCl_3$) δ (ppm) 156.7, 152.0, 130.2 (d, J = 158 Hz), 105.7 (d, J = 160 Hz), 104.3 (d, J = 159 Hz), 100.1 (d, J = 156 Hz), 40.1. HRMS (ESI+) calcd. for [$C_8H_6D_6NO^+$] 144.1290, found 144.1285.

6-(Bis(methyl-*d*$_3$)amino)-2-phenyl-4*H*-chromen-4-one (8).

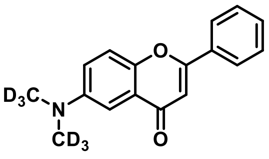

4-(Bis(methyl-*d*$_3$)amino)phenol (**7**) (1.2 g, 8.38 mmol) and ethyl benzoylacetate (2.5 mL, 14.7 mmol) were combined in a 50 mL oven-dried flask equipped with an oven-dried reflux condenser and heated neat to 180°C for 24 h. The solution was cooled down to room temperature, evaporated with Celite and purified by column chromatography (SiO_2, pentane/AcOEt gradient 6:1 – 1:1) to afford flavone **8**. Yield: 0.98 g (43%). Brown solid. M.p. 159.2–159.6°C. ^{1}H NMR (500 MHz, $CDCl_3$) δ (ppm) 8.05 (d, J = 9.0 Hz, 1H), 7.99–7.86 (m, 2H), 7.56–7.47 (m, 3H), 6.93–6.71 (m, 2H), 6.61 (s, 1H).^{2}H NMR (92 MHz, $CDCl_3$) δ (ppm) 3.09 (s, 6H). ^{13}C NMR (126 MHz, $CDCl_3$) δ (ppm) 177.6, 162.7, 158.6, 154.4, 132.2, 131.3, 129.0, 126.7, 126.2, 113.3, 111.1, 106.9, 97.2, 39.4 (sep, J = 21 Hz). ^{13}C{^{2}H} NMR (151 MHz, $CDCl_3$) δ (ppm) 132.3, 131.3 (dt, J_1 = 162, J_2 = 7.6 Hz), 129.1 (d, J = 153 Hz), 126.8 (d, J = 164 Hz), 126.3 (dd, J_1 = 161, J_2 = 6.5 Hz), 113.5, 111.2 (d, J = 158 Hz), 107.1 (d, J = 166 Hz), 97.2 (dd, J_1 = 160, J_2 = 5.2 Hz), 39.6. HRMS (ESI+) calcd. for [$C_{17}H_{10}D_6NO_2^+$] 272.1552, found 272.1542.

6-(Bis(methyl-*d*$_3$)amino)-4-methyl-2-phenylchromenylium tetrafluoroborate (9).

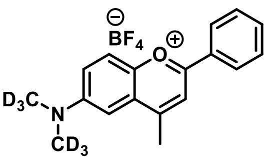

Flavone **8** (336 mg, 1.24 mmol) was dissolved in anhydrous THF (17 mL), cooled to 0 °C in an ice bath, and MeMgBr (1.2 mL, 2.5 M in Et_2O, 3 mmol) was added dropwise. The reaction was left to warm up to room temperature and stirred for 25 h. The reaction was then quenched with HBF_4 (6 mL, 5%, 4.8 mmol), H_2O (50 mL) was added, and the mixture was extracted with CH_2Cl_2 (3×100 mL) dried over $MgSO_4$, filtered, and evaporated. The crude product was purified by trituration with hot AcOEt (70 mL), filtered and washed with Et_2O (3×40 mL) providing flavylium **9**. Yield: 337 mg (69%). Dark red solid. M.p. 256 – 256.5°C (decomp.). ^{1}H NMR (500 MHz, DMSO-*d*$_6$) δ (ppm) 8.36 (d, J = 7.7 Hz, 2H), 8.24–8.20 (m, 2H), 7.80–7.74 (m, 1H), 7.75–7.68 (m, 2H), 7.51 (dd, J_1 = 9.6, J_2 =2.6 Hz, 1H), 7.34 (d, J = 2.5 Hz, 1H), 2.89 (s, 3H).^{2}H NMR (92 MHz, DMSO-*d*$_6$) δ (ppm) 3.30 (s, 6H). ^{13}C NMR (126 MHz, DMSO-*d*$_6$) δ (ppm) 164.7, 164.3, 158.8, 158.1, 134.6, 130.2, 130.0, 129.4, 128.2, 118.7, 118.1, 112.5, 96.5, 20.1.^{13}C{^{2}H} NMR (151 MHz, DMSO-*d*$_6$) δ (ppm) 164.2, 163.8, 158.3 (d, J = 5.4 Hz), 157.9 (dd, J_1 = 97, J_2 =8.4 Hz), 134.1 (d, J = 163 Hz), 129.8 (d, J = 164 Hz), 129.7 (d, J = 163 Hz), 128.9 (d, J = 166 Hz), 127.7 (d, J = 163 Hz), 118.8, 118.2 (d,

J = 166 Hz), 112.0 (dd, J_1 = 173, J_2 = 5.1 Hz), 96.1 (dd, J_1 = 167, J_2 = 5.0 Hz), 19.6 (q, J = 133 Hz). HRMS (ESI+) calcd. for [$C_{18}H_{12}D_6NO^+$] 270.1765, found 270.1765.

***N*-((1*E*,2*E*,4*E*)-5-(Phenylamino)penta-2,4-dien-1-ylidene-1,2,3,4,5-d_5)benzenaminium chloride (6D).**

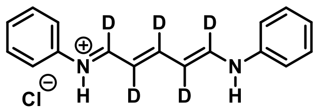

Solution of pyridine-d_5 (1.16 g, 13.8 mmol) and 2,4-dinitrophenyltosylate (5.13 g, 15.2 mmol) in MeCN (10 mL) was stirred at 40°C for 18 h. AcOEt (40 mL) was added to the reaction mixture and after stirring for 1 h, the precipitate was filtered off, washed with Et_2O (3×20 mL), dried and the Zincke salt **5** formed in quantitative yield (5.81 g, 13.8 mmol) as a white solid and was used in the next step without further purification. Aniline (0.46 g, 4.9 mmol) in CD_3OD (80% in D_2O, 3 mL) was added dropwise into a solution of Zincke salt **5** (1.02 g, 2.4 mmol) in CD_3OD (90% in D_2O, 3 mL) and stirred at room temperature for 2 h. The mixture was cooled in ice bath for 30 min, the precipitate was filtered off, washed with Et_2O (15 mL). The filtrate was filtered one more time. The combined solids were suspended in *i*-PrOH (5 mL) containing conc. HCl (32%, 4 mL) and MTBE (25 mL) was added. The precipitate was filtered off, washed with Et_2O/*i*-PrOH (15 mL, 30:1), Et_2O (25 mL) and dried to afford the intermediate **6D**. Note: Even in solid state stored in freezer (-20°C), **6D** undergoes slow shuffling of deuteria and protons on the nitrogen atoms over time, decreasing its deuteration degree, and it is advised to prepare it shortly before use. Yield: 400 mg (57%). Red solid. M.p. 180–181°C. ^{1}H NMR (600 MHz, DMSO-d_6) δ (ppm) 12.19 (s, 2H), 7.49–7.42 (m, 8H), 7.24–7.21 (m, 2H). ^{2}H NMR (61 MHz, DMSO-d_6) δ (ppm) 9.18–8.27 (m, 2D), 8.00–7.87 (m, 1D), 6.91–6.11 (m, 2D). ^{13}C NMR (151 MHz, DMSO-d_6) δ (ppm) 138.8, 129.8, 125.6, 117.6. ^{13}C{^{2}H} NMR (151 MHz, DMSO-d_6) δ (ppm) 162.6, 155.0, 138.8, 129.8 (d, J = 161 Hz), 125.6 (d, J = 162 Hz), 117.6 (d, J = 161 Hz), 108.9. HRMS (ESI+) calcd. for [$C_{17}H_{12}D_5N_2^+$] 254.1700, found 254.1698.

General Procedure for the Synthesis of Cyanines 1H–DD.

Intermediate **6H** or **6D** (1 eq., 0.35 mmol) was dissolved in anhydrous MeCN (4 mL) under nitrogen atmosphere. DIPEA (3 eq., 136 mg, 1.05 mmol) was added dropwise, and the mixture was cooled in an ice bath. Ac_2O (5 eq., 0.16 mL, 1.75 mmol) was added dropwise, and the reaction mixture was stirred at room temperature for 45 min. The mixture was degassed (3 cycles of sonication + vacuum/N_2 overpressure, 1 min. each) and a degassed solution of heterocycle **9** or **10** (3 eq., 1.05 mmol; degassed again by 3 cycles) in CD_3OD/MeCN-d_3 (for **1D**, **1DD**) or CH_3OH/MeCN (for **1**) (8 mL; 1:1) was added. The reaction mixture was carefully degassed again (3 cycles of sonication + vacuum/N_2 overpressure, 1 min. each). The reaction flask was covered in aluminum foil and stirred at room temperature for 20 h. The volatiles were evaporated under reduced pressure, the crude product was dissolved in MeOH (4 mL) and added dropwise into stirred Et_2O (25 mL). The precipitate was filtered, washed with H_2O (10 mL) and Et_2O (10 mL). The resulting solid was purified by column chromatography (SiO_2, CH_2Cl_2/EtOH gradient 200:1 – 50:1 or CH_2Cl_2/MeOH gradient 200:1 – 100:1) to afford the target cyanines.

7-(Diethylamino)-4-((1*E*,3*E*,5*E*)-7-((*E*)-7-(diethylamino)-2-phenyl-4*H*-chromen-4-ylidene)hepta-1,3,5-trien-1-yl)-2-phenylchromenylium tetrafluoroborate (1H).

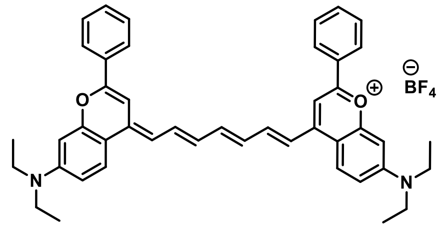

Prepared according to the general procedure from intermediate **6H** (105 mg, 0.35 mmol) and heterocycle **10** (398 mg, 1.05 mmol) in CH_3OH/MeCN (8 mL; 1:1). Purified by column chromatography (SiO_2, CH_2Cl_2/EtOH gradient 200:1 – 50:1). Yield: 187 mg (73%). Purple solid. M.p. 180.3–180.5°C (decomp.). ^{1}H NMR (400 MHz, DMSO-d_6) δ (ppm) 8.25–8.13 (m, 6H), 8.03 (d, J = 9.3 Hz, 2H), 7.84 (s, 2H), 7.65–7.59 (m, 6H), 7.44 – 7.33 (m, 1H), 7.12 (d, J = 13.3 Hz, 2H), 7.01 (dd, J_1 = 9.4, J_2 = 2.5 Hz, 2H), 6.89 (d, J = 2.5 Hz, 2H), 6.84–6.73 (m, 2H), 3.56 (q, J = 7.1 Hz, 8H), 1.20 (t, J = 7.0 Hz, 12H). ^{13}C NMR (126 MHz, DMSO-d_6) δ (ppm) 156.3, 156.2, 152.6, 146.9, 144.4, 131.9, 131.8, 129.7, 129.5, 126.6, 126.5, 116.4, 113.5, 111.4, 102.3, 97.6, 44.9, 13.0. HRMS (ESI+) calcd. for [$C_{45}H_{45}N_2O_2^+$] 645.3481, found 645.3483.

7-(Diethylamino)-4-((1*E*,3*E*,5*E*)-7-((*E*)-7-(diethylamino)-2-phenyl-4*H*-chromen-4-ylidene)hepta-1,3,5-trien-1-yl-1,2,3,4,5,6,7-*d*$_7$)-2-phenylchromenylium tetrafluoroborate (1D).

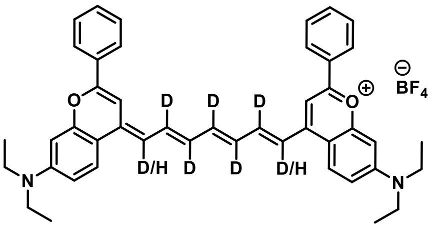

Prepared according to the general procedure from intermediate **6D** (107 mg, 0.35 mmol) and heterocycle **10** (398 mg, 1.05 mmol) in CD_3OD/MeCN-d_3 (8 mL; 1:1) as a mixture of d_7:d_6:d_5 – 42:32:26. Purified by column chromatography (SiO_2, CH_2Cl_2/MeOH gradient 200:1 – 100:1). Yield: 130 mg (54%). Purple solid. M.p. 152–153°C. ^{1}H NMR (600 MHz, DMSO-d_6) δ (ppm) 8.27–8.11 (m, 4H), 8.06–7.89 (m, 2H), 7.81–7.70 (m, 2H), 7.66–7.55 (m, 6H), 7.06 (s, 1H), 7.00–6.92 (m, 2H), 6.87–6.80 (m, 2H), 3.54 (q, J = 7.3 Hz, 8H), 1.20 (t, J = 7.1 Hz, 12H). ^{2}H NMR (92 MHz, MeCN-d_3) δ (ppm) 8.33–7.33 (m, 2H), 7.41–6.14 (m, 5H). ^{13}C NMR (126 MHz, DMSO-d_6) δ (ppm) 155.8, 152.1, 131.4, 131.4, 129.1, 126.2, 126.0, 113.1, 110.9, 101.9, 97.1, 44.5, 12.5. ^{13}C{^{2}H} NMR (151 MHz, DMSO-d_6) δ (ppm) 156.2, 152.5, 150.1, 146.4, 144.3, 131.9 (d, J = 163 Hz), 131.8, 130.1 129.5 (d, J = 158 Hz), 129.2, 126.6 (d, J = 161 Hz), 126.4 (d, J = 160 Hz), 116.8, 116.0, 113.5 (d, J = 162 Hz), 110.9, 101.9 (d, J = 165 Hz), 97.1 (d, J = 163 Hz), 44.5 (t, J = 136 Hz), 12.5 (q, J = 127 Hz). HRMS (ESI+) calcd. for [$C_{45}H_{38}D_7N_2O_2^+$] 652.3920, found 652.3895.

7-(Bis(methyl-*d*$_3$)amino)-4-((1*E*,3*E*,5*E*)-7-((*E*)-7-(bis(methyl-*d*$_3$)amino)-2-phenyl-4*H*-chromen-4-ylidene)hepta-1,3,5-trien-1-yl-1,2,3,4,5,6,7-*d*$_7$)-2-phenylchromenylium tetrafluoroborate (1DD).

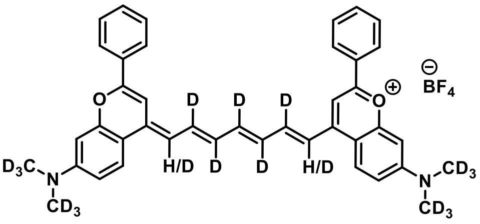

Prepared according to the general procedure from intermediate **6D** (107 mg, 0.35 mmol) and heterocycle **9** (375 mg, 1.05 mmol) in CD_3OD/MeCN-d_3 (8 mL; 1:1) as a mixture of d_7:d_6:d_5 – 11:34:55. Purified by column chromatography (SiO_2, CH_2Cl_2/MeOH gradient 200:1 – 100:1). Yield: 51 mg (21%). Purple solid. M.p. 196.4–197.0°C (decomp.). ^{1}H NMR (500 MHz, MeCN-d_3) δ (ppm) 7.61 (d, J = 7.4 Hz, 4H), 7.40–7.25 (m, 9H), 6.96 (s, 2H), 6.48 (s, 1.48 H), 6.32 (dd, J_1 = 9.1, J_2 = 2.6 Hz, 2H), 6.19 (d, J = 2.7 Hz, 2H). ^{13}C NMR (126 MHz, MeCN-d_3) δ (ppm) 156.5, 156.1, 154.8, 132.2, 131.9, 129.7, 126.5, 125.8, 116.6, 113.2, 112.1, 102.1, 97.8. ^{13}C{^{2}H} NMR (151 MHz, MeCN-d_3) δ (ppm) 156.2, 155.9, 154.5 (d, J = 9.1 Hz), 145.2 (d, J = 243 Hz), 132.4, 132.0, 131.2, 130.2, 129.6 (d, J = 161 Hz), 129.5 (d, J = 161 Hz), 126.5 (d, J = 107 Hz), 125.5

(d, J = 106 Hz), 116.6 (d, J = 109), 113.0 (d, J = 161 Hz), 111.9, 102.0 (d, J = 168 Hz), 97.6 (d, J = 166 Hz), 39.4. HRMS (ESI+) calcd. for [$C_{41}H_{19}D_{18}N_2O_2^+$] 607.3985, found 607.3975.

General Procedure for the Synthesis of 2H–D.
Solution of heterocycle **13** (2 eq., 226 mg, 0.84 mmol), intermediate **6H** or **6D** (1 eq, 0.42 mmol), and sodium acetate (2 eq., 69 mg, 0.84 mmol) in Ac_2O (16 mL) was stirred at room temperature for 2 h. Afterwards, Et_2O (30 mL) was added, and the mixture was cooled for 3 h to 4°C. The precipitate was filtered, the filtrate diluted with ether (50 mL) and filtered again. Combined solids were washed with Et_2O (50 mL), H_2O (25 mL) and again Et_2O (20 mL). The crude product was washed with MeOH (20 mL), AcOEt (10 mL) and Et_2O (20 mL) to afford pure cyanines **2H**–**D**.

1-Methyl-2-((1*E*,3*E*,5*E*,7*E*)-7-(1-methylbenzo[*cd*]indol-2(1*H*)-ylidene)hepta-1,3,5-trien-1-yl)benzo[*cd*]indol-1-ium tetrafluoroborate (2H).

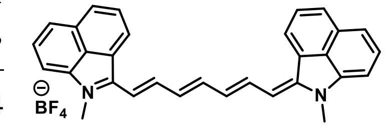

Prepared according to the general procedure from intermediate **6H** (120 mg, 0.42 mmol) and heterocycle **13** (226 mg, 0.84 mmol). Yield: 132 mg (61%). Purple solid. M.p. 243.5–243.7°C (decomp.). ^{1}H NMR (400 MHz, MeCN-d_3) δ (ppm) 8.24 (d, J = 7.2 Hz, 2H), 8.04–7.94 (m, 4H), 7.81–7.71 (m, 2H), 7.52 (d, J = 8.4 Hz, 3H), 7.45–7.35 (m, 2H), 6.96 (d, J = 7.2 Hz, 2H), 6.87–6.76 (m, 2H), 6.43 (d, J = 13.6 Hz, 2H), 3.38 (s, 6H). ^{13}C NMR not measured due to poor solubility. HRMS (ESI+) calcd. for [$C_{31}H_{25}N_2^+$] 425.2018, found 425.2024.

1-Methyl-2-((1*E*,3*E*,5*E*,7*E*)-7-(1-methylbenzo[*cd*]indol-2(1*H*)-ylidene)hepta-1,3,5-trien-1-yl-1,2,3,4,5,6,7-*d*$_7$)benzo[*cd*]indol-1-ium tetrafluoroborate (2D).

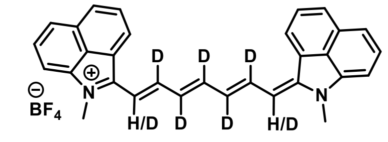

Prepared according to the general procedure from intermediate **6D** (123 mg, 0.42 mmol) and heterocycle **13** (226 mg, 0.84 mmol) as a mixture of d_7:d_6:d_5 – 1:18:81. Yield: 113 mg (52%). Purple solid. M.p. 243.7–244°C (decomp.). ^{1}H NMR (400 MHz, MeCN-d_3) δ (ppm) 8.31 (d, J = 7.2 Hz, 2H), 8.05 (d, J = 8.0 Hz, 2H), 7.85–7.75 (m, 2H), 7.57 (d, J = 8.2 Hz, 2H), 7.50–7.44 (m, 2H), 7.06 (d, J = 7.2 Hz, 2H), 6.50 (s, 1.8H), 3.47 (s, 6H). ^{2}H NMR (92 MHz, MeCN-d_3) δ 9.03–7.30 (m, 4D), 7.13–6.16 (m, 3D).^{13}C NMR not measured due to low solubility. HRMS (ESI+) calcd. for [$C_{31}H_{20}D_5N_2^+$] 430.2332, found 430.2320.

General Procedure for the Synthesis of 3H–D and 4D.
The Schiff base **6H** or **6D** (1 eq., 0.33 mmol) was dissolved in dry MeCN (5 mL) and kept under N_2 atmosphere. DIPEA (3 eq., 127 mg, 0.98 mmol) was added dropwise, and the mixture was cooled in ice bath, followed by the addition of Ac_2O (5 e.q., 0.15 mL, 1.6 mmol). The red solution turned yellow within few minutes. Heterocycle **11** or **12** (3 eq., 0.98 mmol) was then dissolved in MeCN/CH_3OH or MeCN-d_3/CD_3OD (1.5 ml; 2:1) with DIPEA (3 eq., 127 mg, 0.98 mmol) and the solution was added to the reaction mixture. The reaction flask was covered in aluminum foil and stirred at room temperature for 16 h. The volatiles were evaporated under reduced pressure to give the crude product, to which Et_2O (5 mL) was added, the precipitate was filtered off and washed with water (5×10 mL) and diethyl ether (3×10 mL) and dried under reduced pressure to give the target cyanines **3H–4D** if not stated differently.

1-Methyl-2-((1*E*,3*E*,5*E*)-7-((*E*)-1-methylquinolin-2(1*H*)-ylidene)hepta-1,3,5-trien-1-yl)quinolin-1-ium iodide (3H).

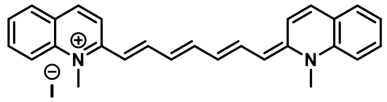

Prepared according to the general procedure from intermediate **6H** (100 mg, 0.33 mmol) and heterocycle **11** (288 mg, 0.98 mmol). Yield: 117 mg (70%). Dark green solid. M.p. 226.7–227.0°C (decomp.). ^{1}H NMR (500 MHz, DMSO-d_6) δ (ppm) 7.98–7.89 (m, 4H), 7.88–7.78 (m, 6H), 7.72 (ddd, J_1 = 8.6, J_2 =7.0, J_3 =1.6 Hz, 2H), 7.47–7.39 (m, 2H), 7.27–7.17 (m, 1H), 6.54–6.45 (m, 2H), 6.39 (d, J = 13.2 Hz, 2H), 3.86 (s, 6H). ^{13}C NMR (126 MHz, DMSO-d_6) δ (ppm) 151.1, 150.3, 146.8, 140.1, 135.2, 132.3, 128.8, 124.8, 124.7, 124.1, 120.1, 116.3, 108.1, 36.2. HRMS (ESI+) calcd. for [$C_{27}H_{25}N_2^+$] 377.2018, found 377.2002.

1-Methyl-2-((1*E*,3*E*,5*E*)-7-((*E*)-1-methylquinolin-2(1*H*)-ylidene)hepta-1,3,5-trien-1-yl-1,2,3,4,5,6,7-*d*$_7$)quinolin-1-ium iodide (3D).

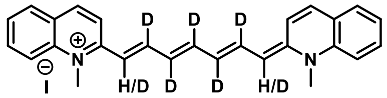

Prepared according to the general procedure from intermediate **6D** (101 mg, 0.33 mmol) and heterocycle **11** (288 mg, 0.98 mmol) as a mixture of d_7:d_6:d_5, 52:40:8. Yield: 135 mg (80%). Dark green solid. M.p. 226.9–227.2°C (decomp.). ^{1}H NMR (500 MHz, DMSO-d_6) δ 7.99–7.89 (m, 4H), 7.87–7.76 (m, 4H), 7.76–7.68 (m, 2H), 7.48–7.37 (m, 2H), 6.38 (s, 0.5H). ^{2}H NMR (92 MHz, DMSO-d_6) δ (ppm) 8.25–7.00 (m, 1D), 6.53 (s, 6D). ^{13}C NMR (126 MHz, DMSO-d_6) δ (ppm) 151.0, 140.0, 135.2, 132.3, 128.8, 124.7, 120.1, 116.3, 36.2. ^{13}C{^{2}H} NMR (151 MHz, DMSO-d_6) δ (ppm) 150.0, 145.4, 139.1, 134.3 (d, J = 168 Hz), 132.0, 131.4 (d, J = 159 Hz), 127.9 (d, J = 162 Hz), 123.8, 123.7 (d, J = 165 Hz), 122.6, 119.1 (d, J = 169 Hz), 115.4 (d, J = 166 Hz) 106.8, 35.3 (q, J = 142 Hz). HRMS (ESI+) calcd. for [$C_{27}H_{18}D_7N_2^+$] 384.2457, found 384.2459.

Sodium 4-(2-((1*E*,3*E*,5*E*,7*E*)-7-(1,1-dimethyl-3-(4-sulfonatobutyl)-1,3-dihydro-2*H*-benzo[*e*]indol-2-ylidene)hepta-1,3,5-trien-1-yl-1,2,3,4,5,6,7-*d*$_7$)-1,1-dimethyl-1*H*-benzo[*e*]indol-3-ium-3-yl)butane-1-sulfonate (4D).

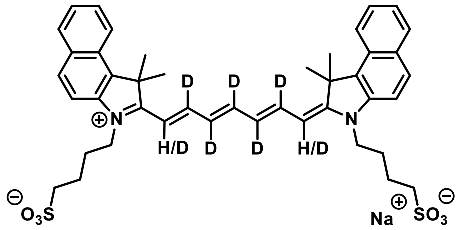

Prepared according to the general procedure from intermediate **6D** (101 mg, 0.33 mmol) and heterocycle **12** (339 mg, 0.98 mmol). The reaction mixture was evaporated under reduced pressure, dissolved in CH_2Cl_2 and washed with brine. Organic layer was separated, dried with $MgSO_4$ and evaporated. Crude product was purified by column chromatography (SiO_2, gradient CH_2Cl_2 /CH_2Cl_2-MeOH 5:1) to afford cyanine **4D** as a mixture of d_7:d_6:d_5, 8:37:55. Yield: 120 mg (47%). Dark green solid. Characterization in accordance with literature.[3]

NMR Spectroscopy

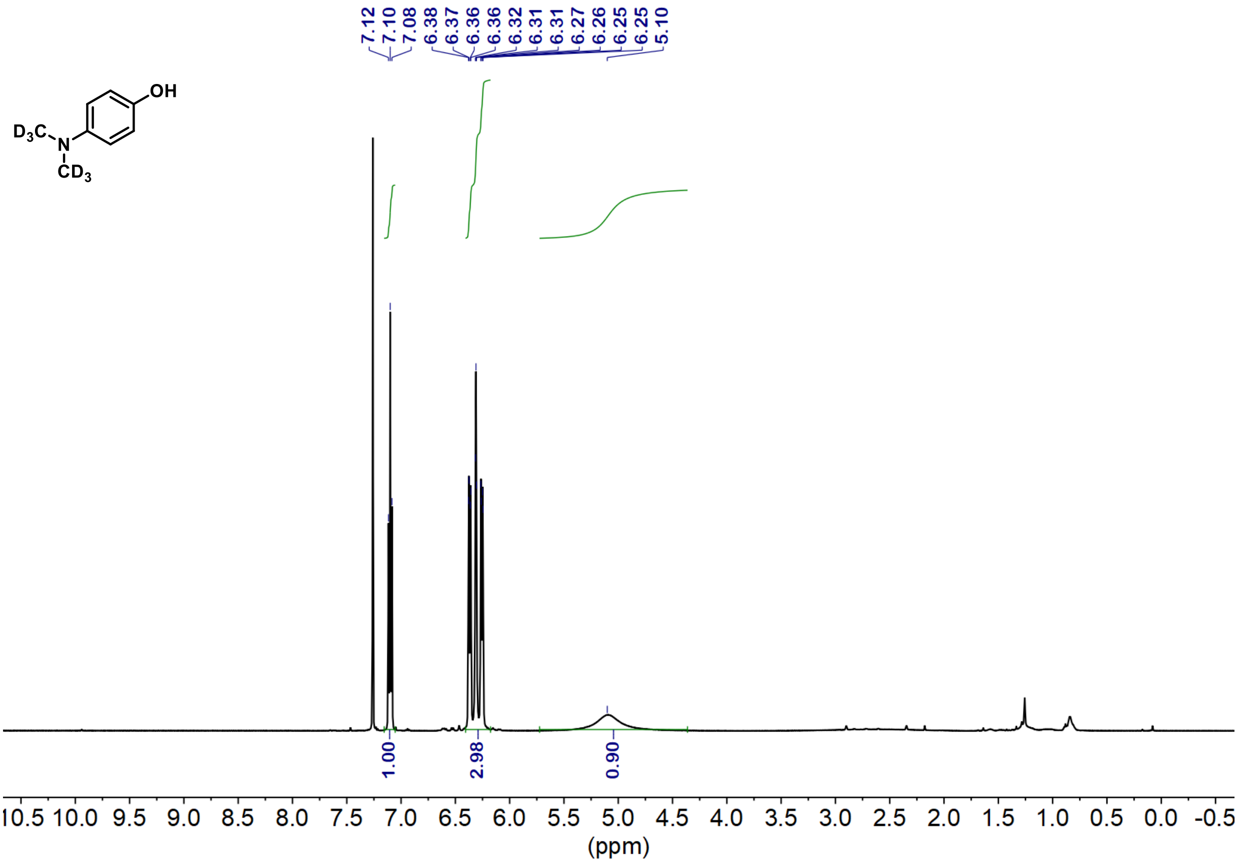

Figure S3. ^{1}H NMR (500 MHz, $CDCl_3$): **7**

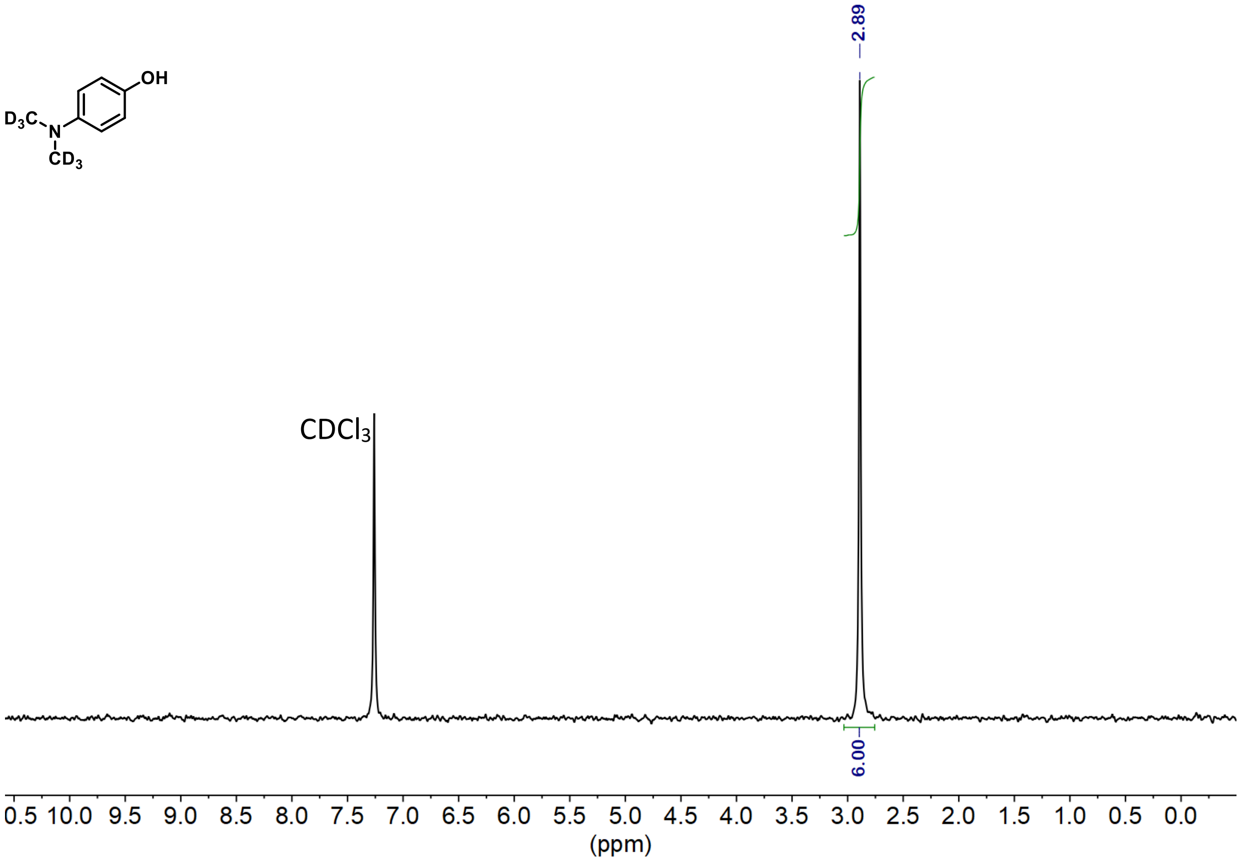

Figure S4. ^{2}H NMR (92 MHz, $CDCl_3$): **7**

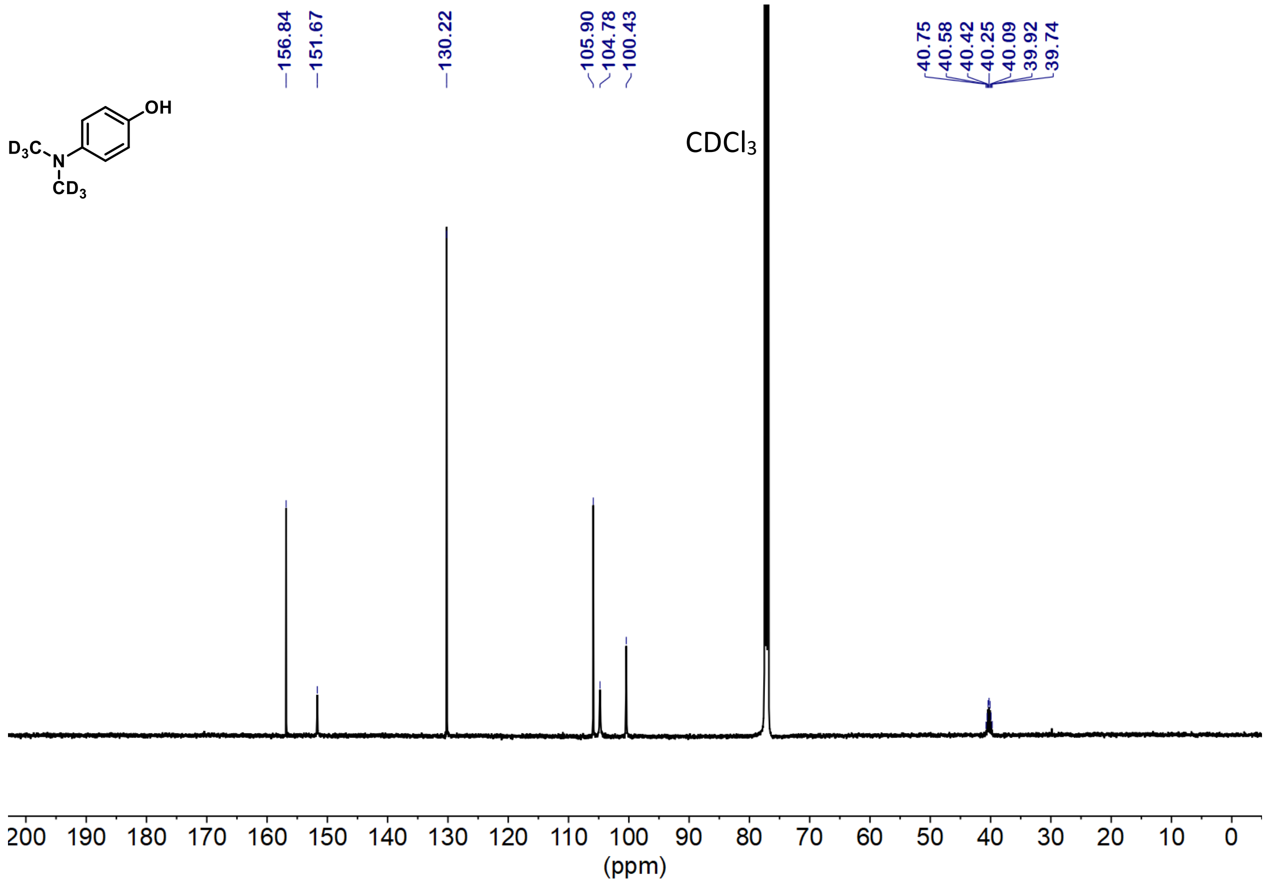

Figure S5. $^{13}C\{^{1}H\}$ NMR (126 MHz, $CDCl_3$) of **7**

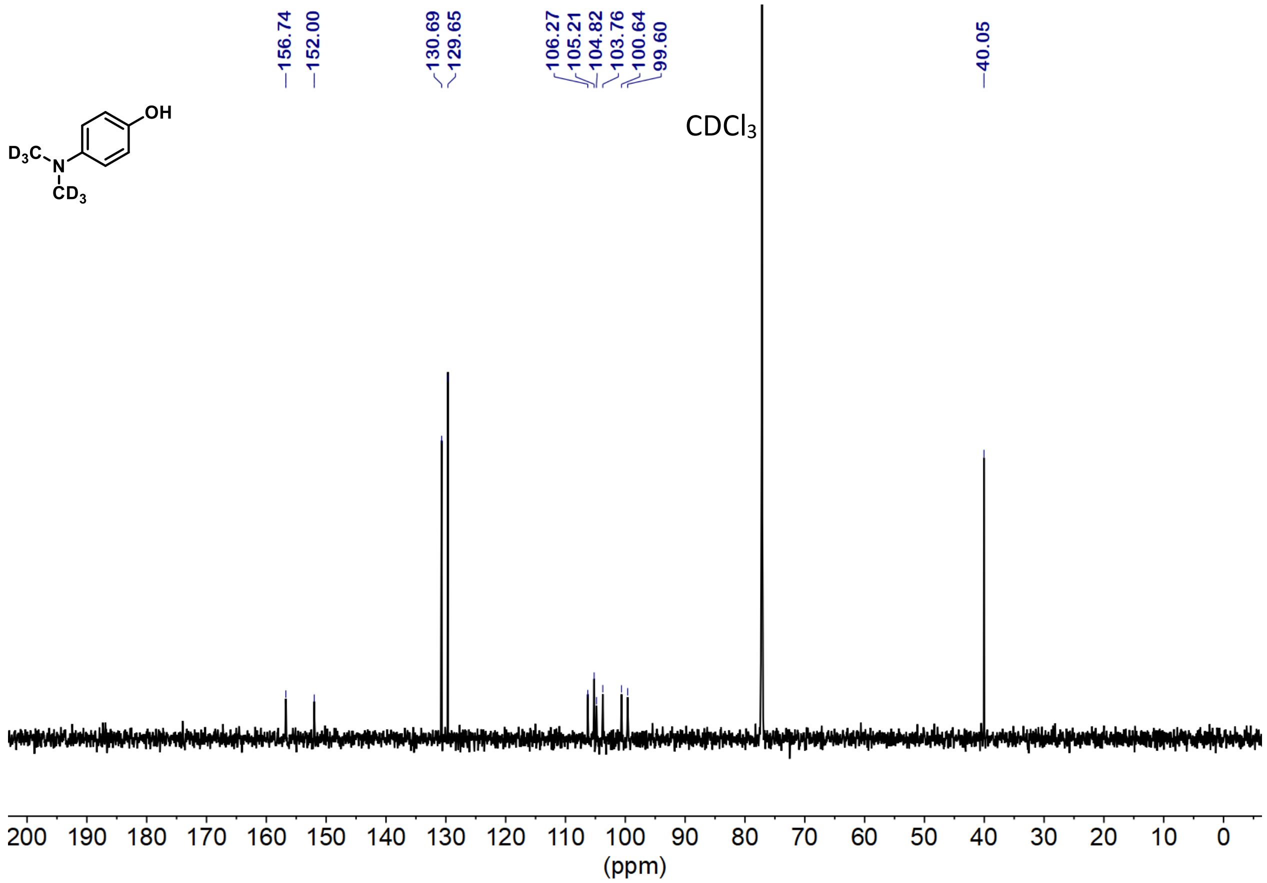

Figure S6. $^{13}C\{^{2}H\}$ NMR (151 MHz, $CDCl_3$): **7**

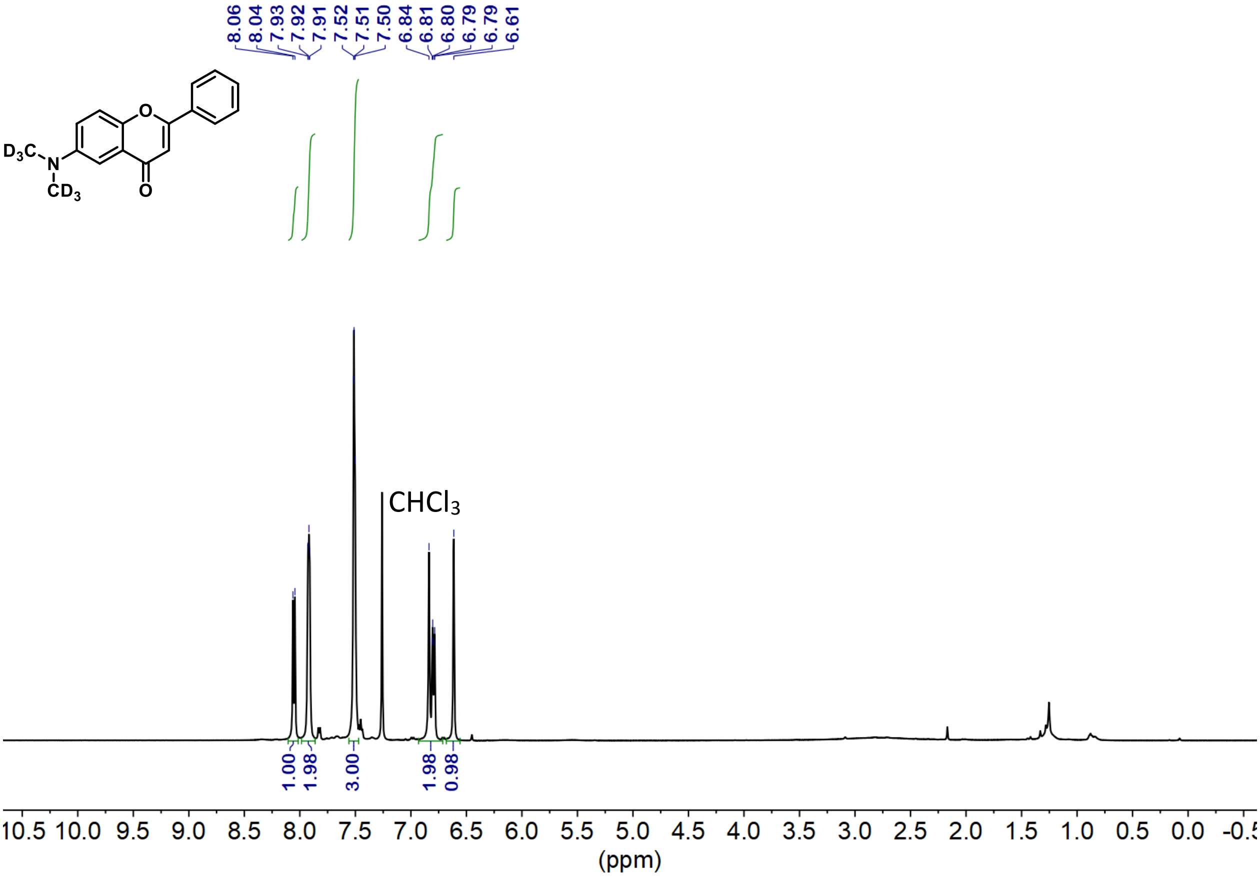

Figure S7. ${}^{1}H$ NMR (500 MHz, $CDCl_3$): **8**

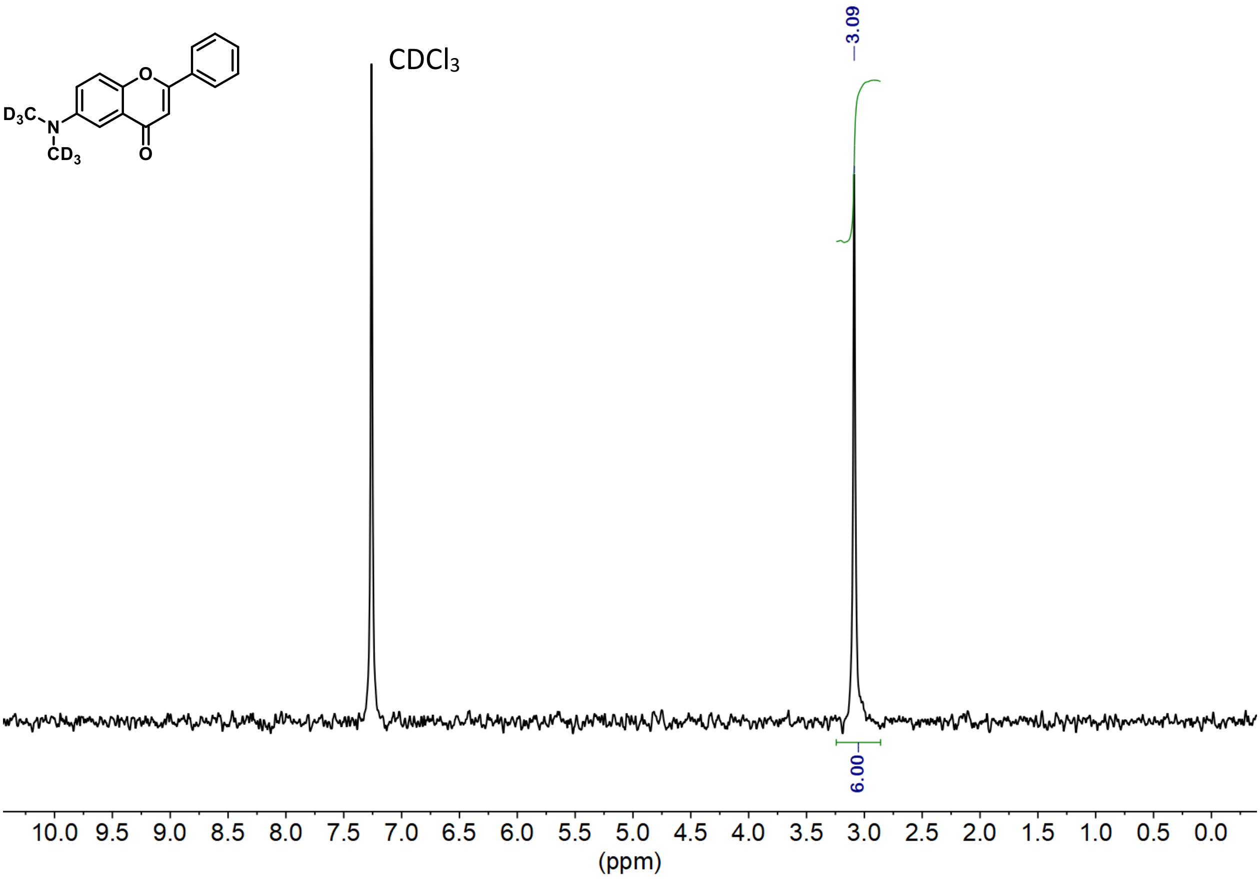

Figure S8. ${}^{2}H$ NMR (92 MHz, $CHCl_3$): **8**

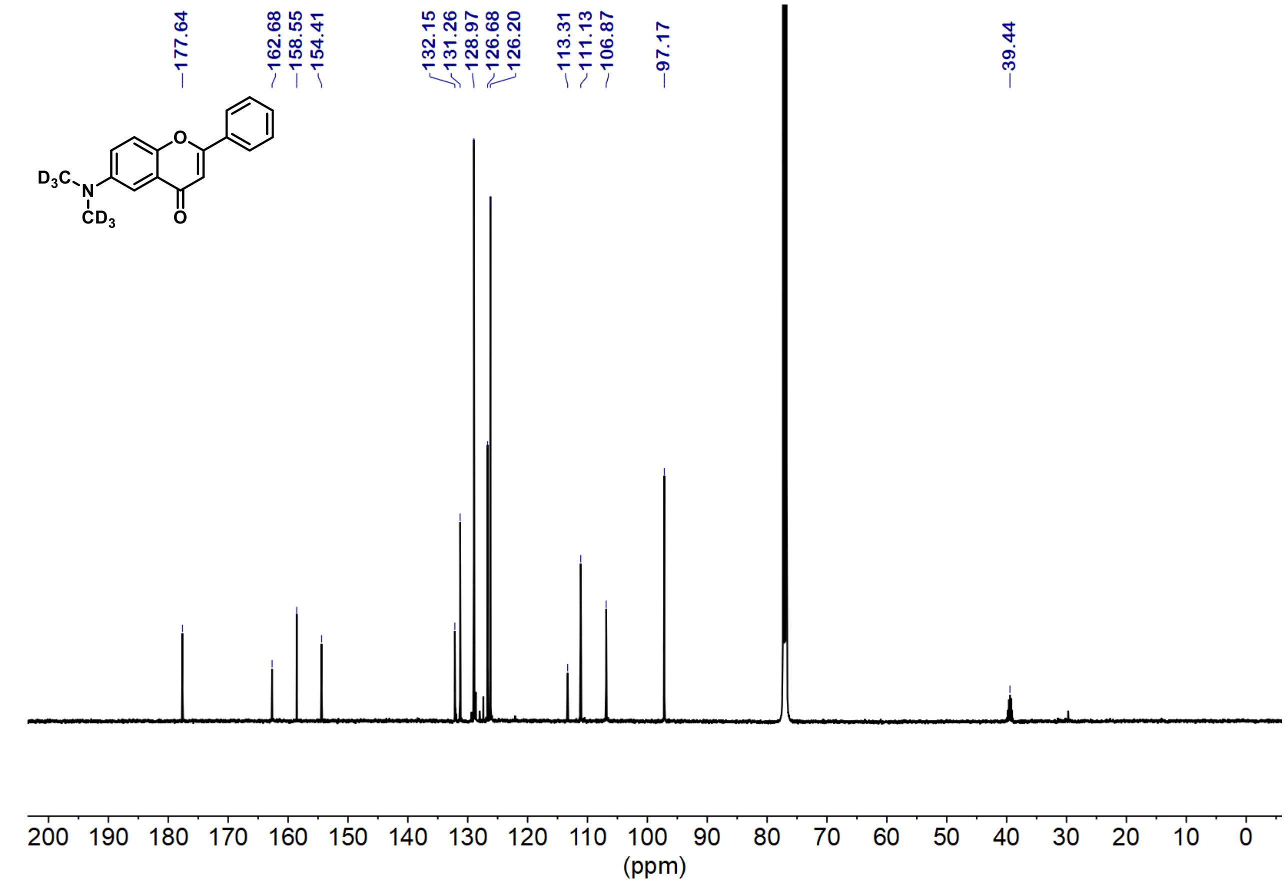

Figure S9. $^{13}C\{^{1}H\}$ NMR (126 MHz, $CDCl_3$): **8**

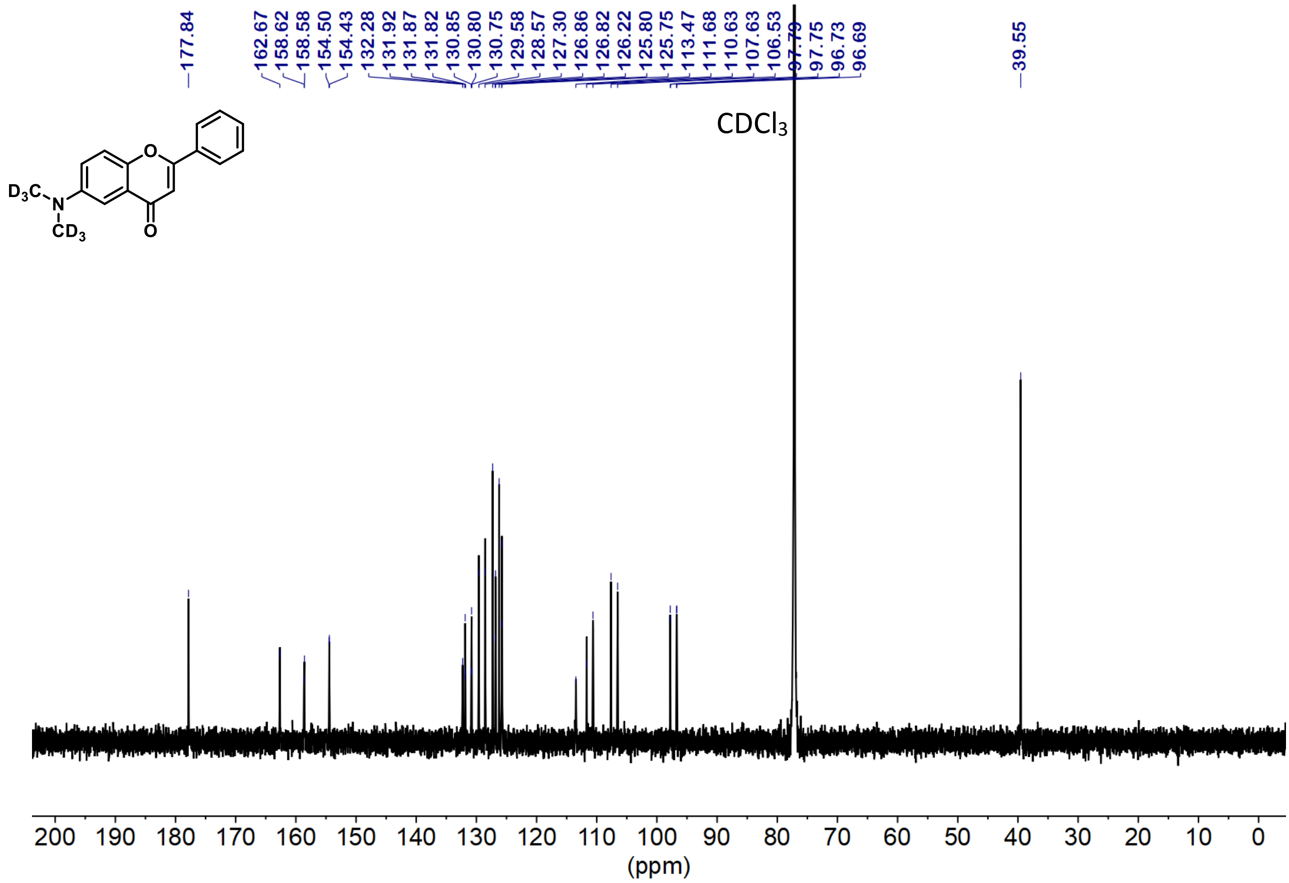

Figure S10. $^{13}C\{^{2}H\}$ NMR (151 MHz, $CDCl_3$): **8**

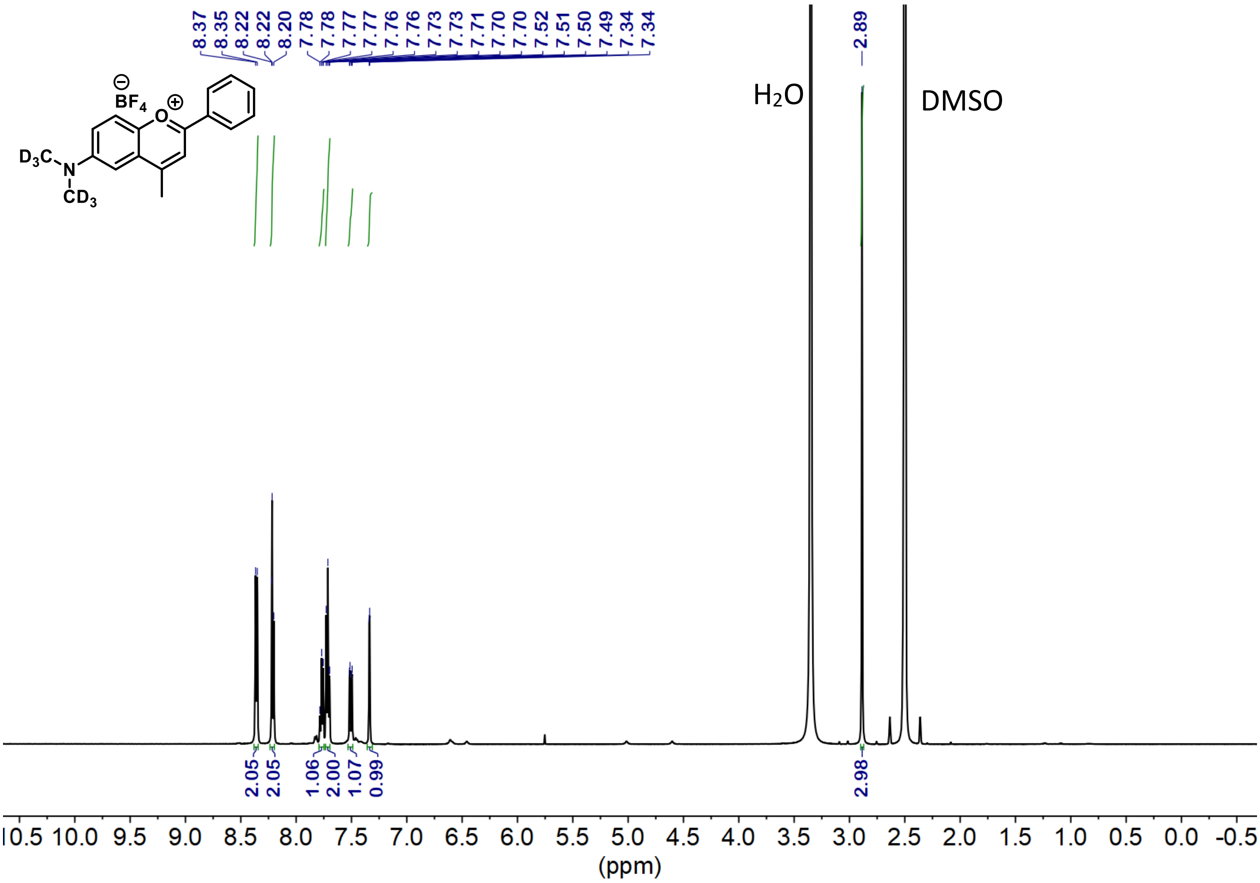

Figure S11. ^{1}H NMR (500 MHz, DMSO-*d*$_6$): **9**

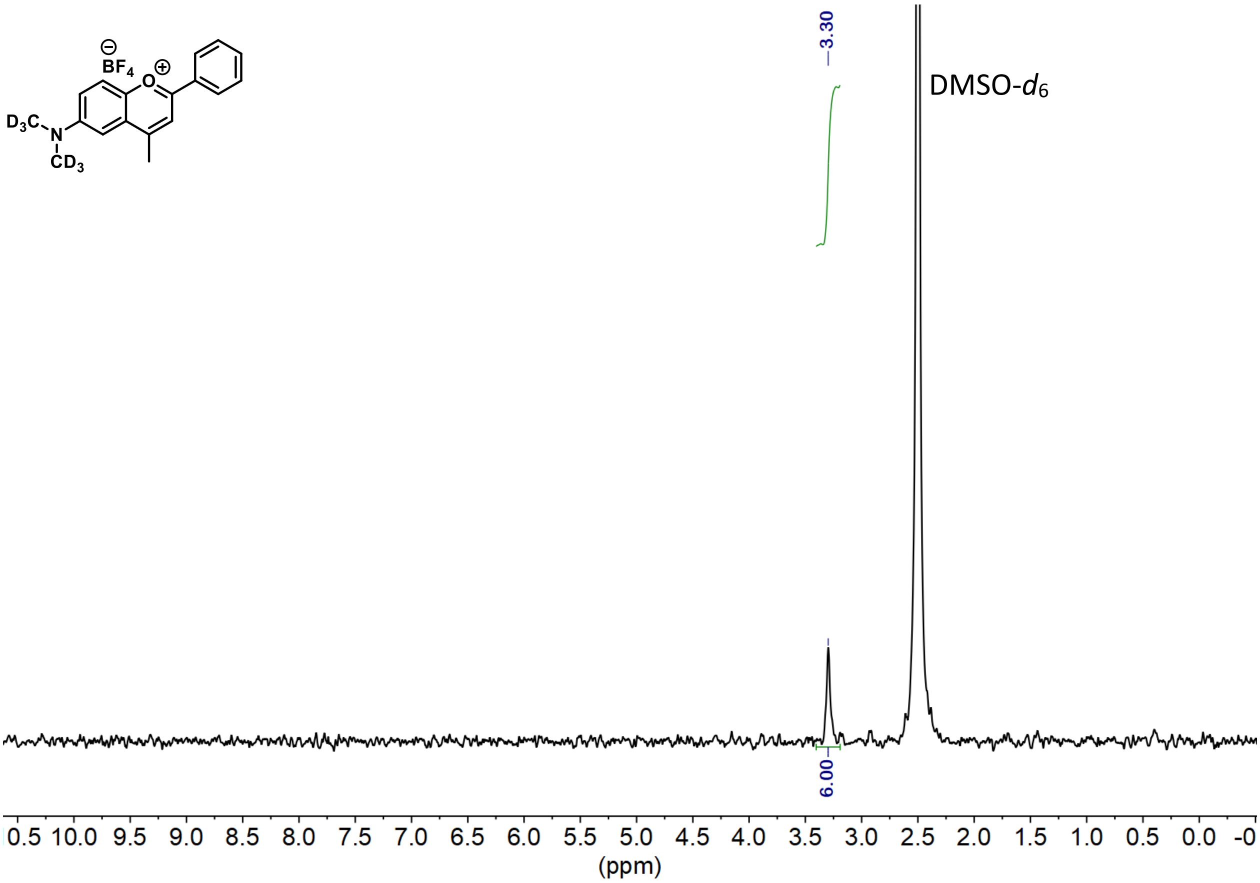

Figure S12. ^{2}H NMR (92 MHz, DMSO): **9**

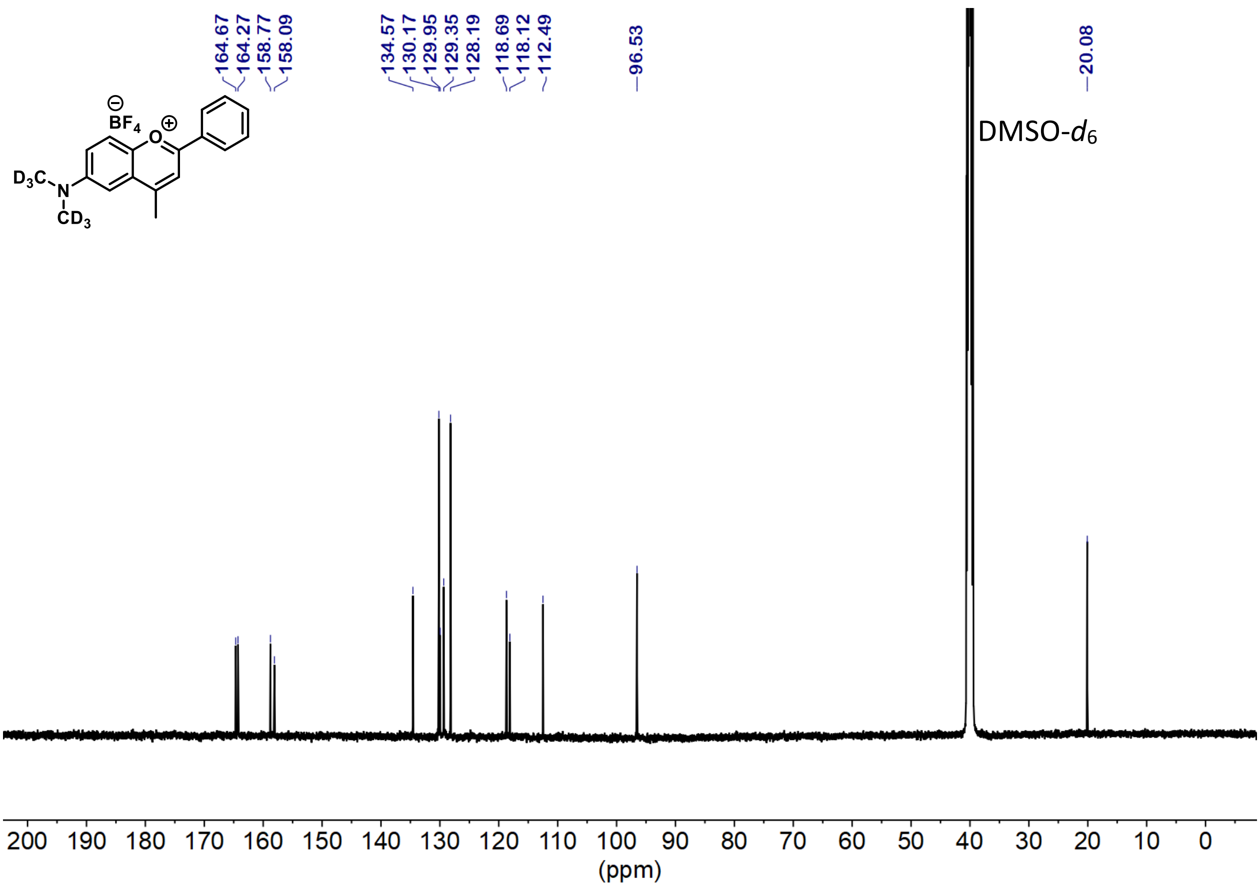

Figure S13. $^{13}C\{^{1}H\}$ NMR (126 MHz, DMSO-d_6): **9**

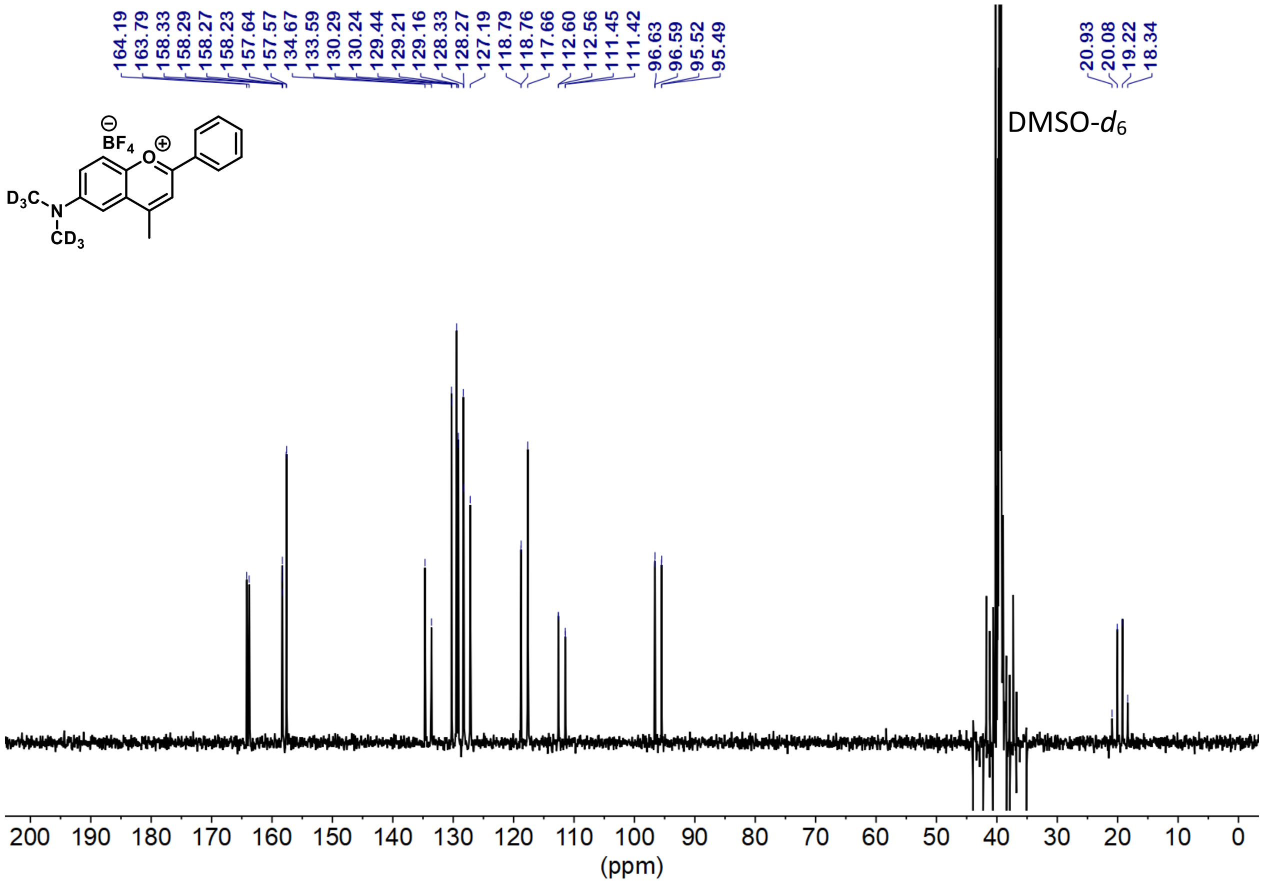

Figure S14. $^{13}C\{^{2}H\}$ NMR (151 MHz, DMSO-d_6): **9**

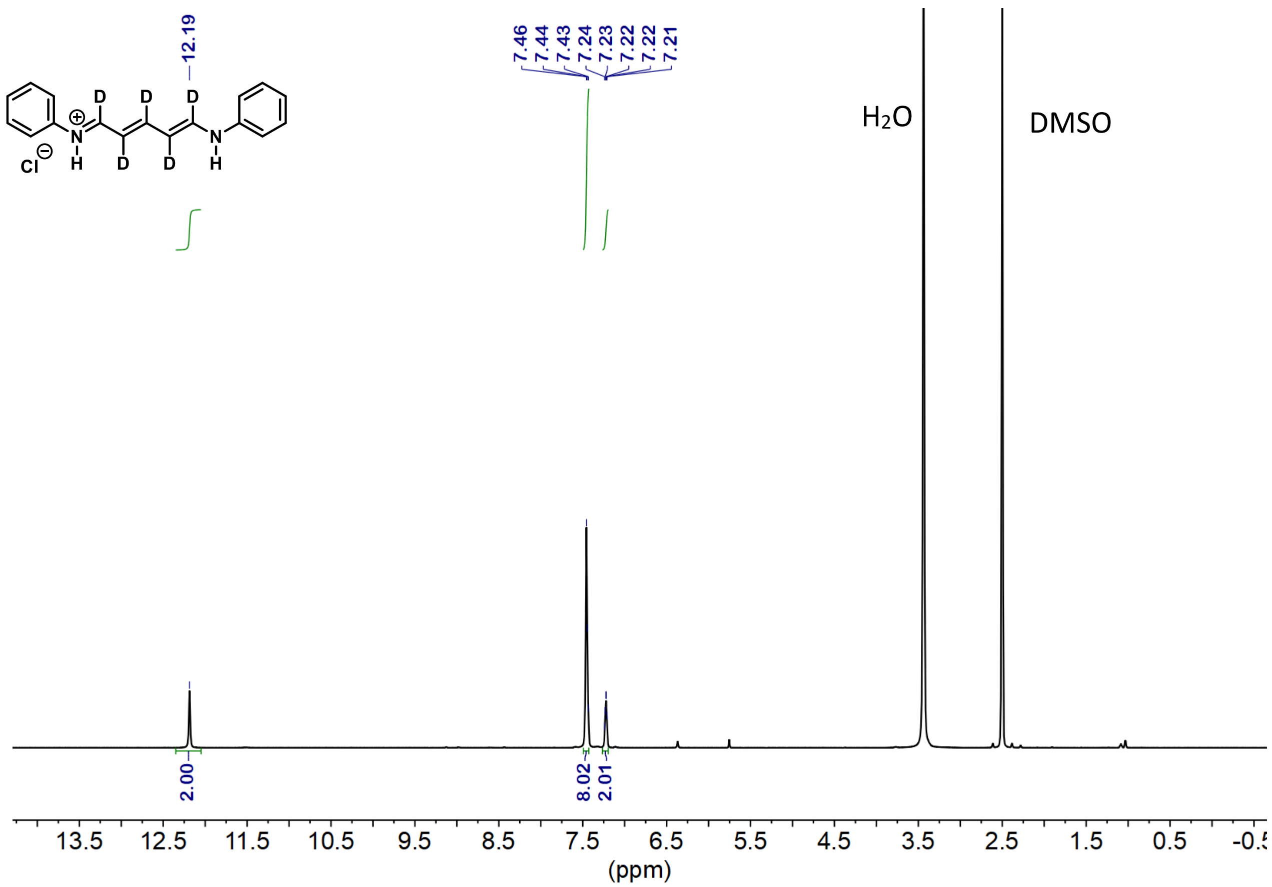

Figure S15. ^{1}H NMR (600 MHz, DMSO-d_6): **6D**

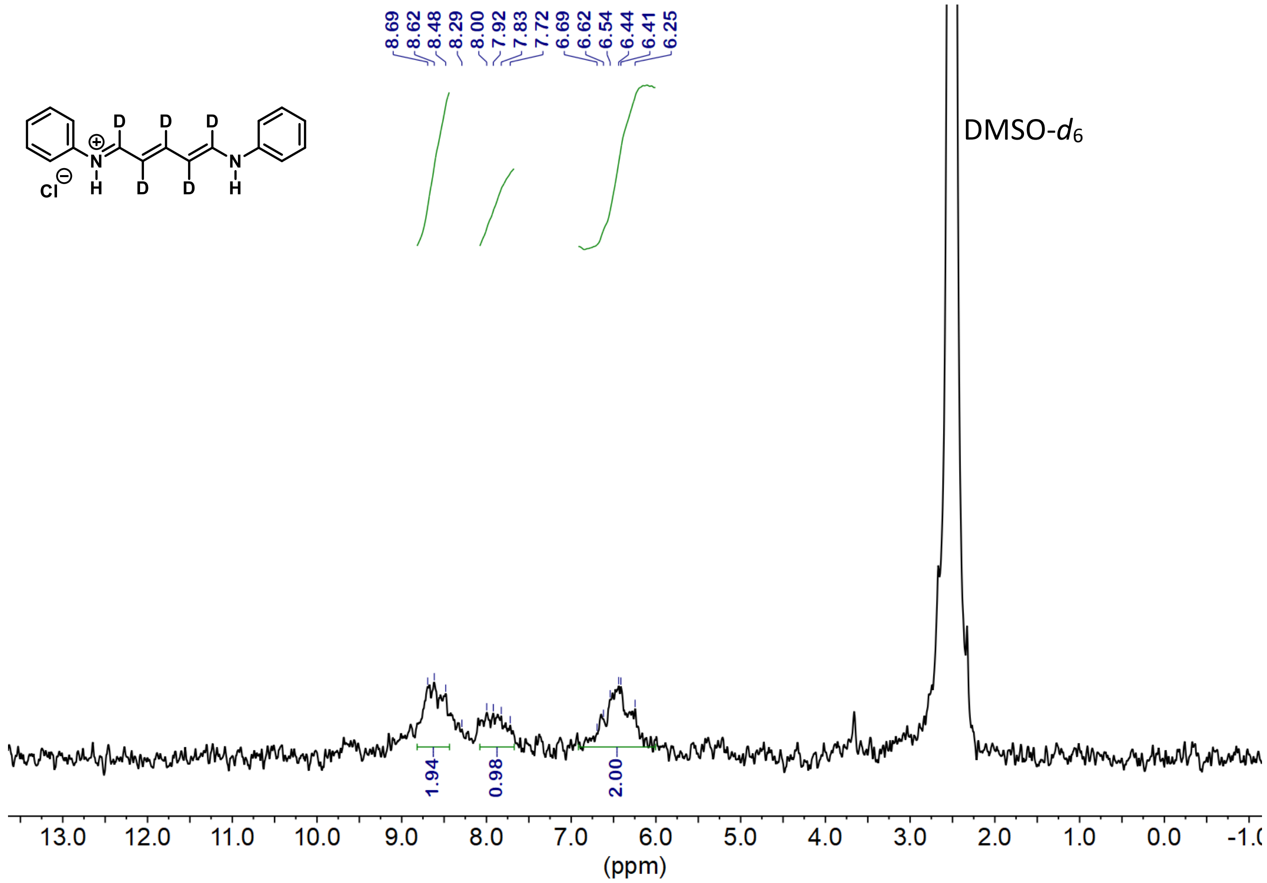

Figure S16. ^{2}H NMR (61 MHz, DMSO-d_6): **6D**

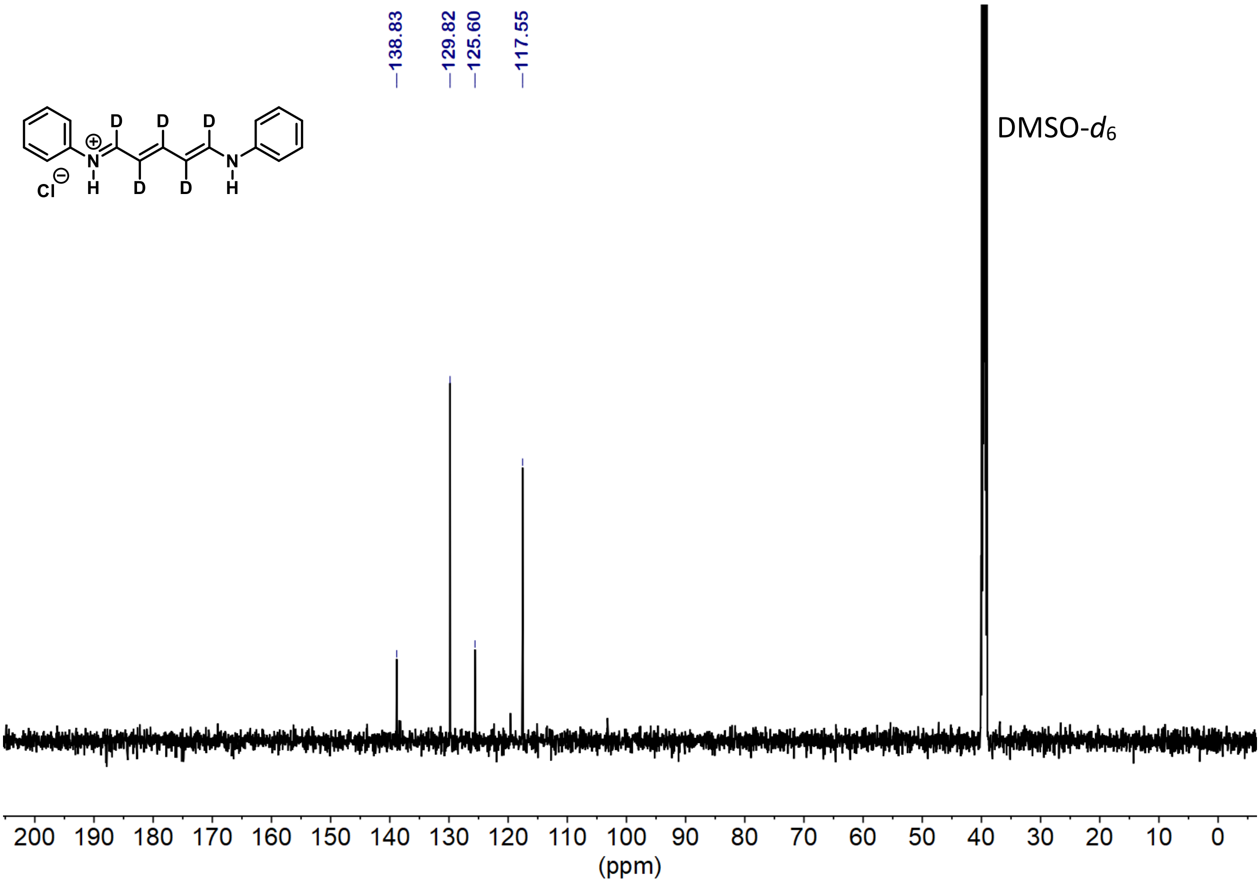

Figure S17. $^{13}C\{^1H\}$ NMR (151 MHz, DMSO-d_6): **6D**

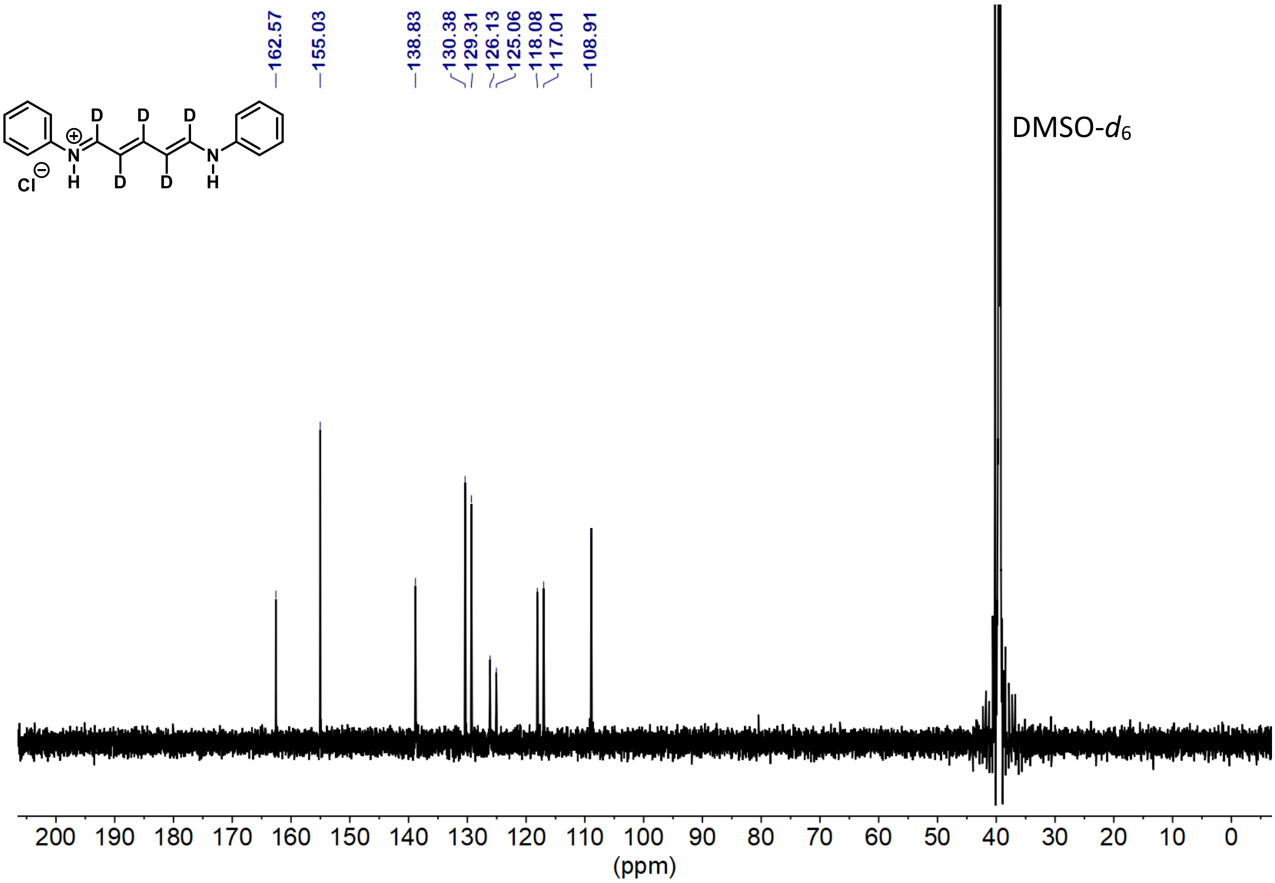

Figure S18. $^{13}C\{^2H\}$ NMR (151 MHz, DMSO-d_6): **6D**

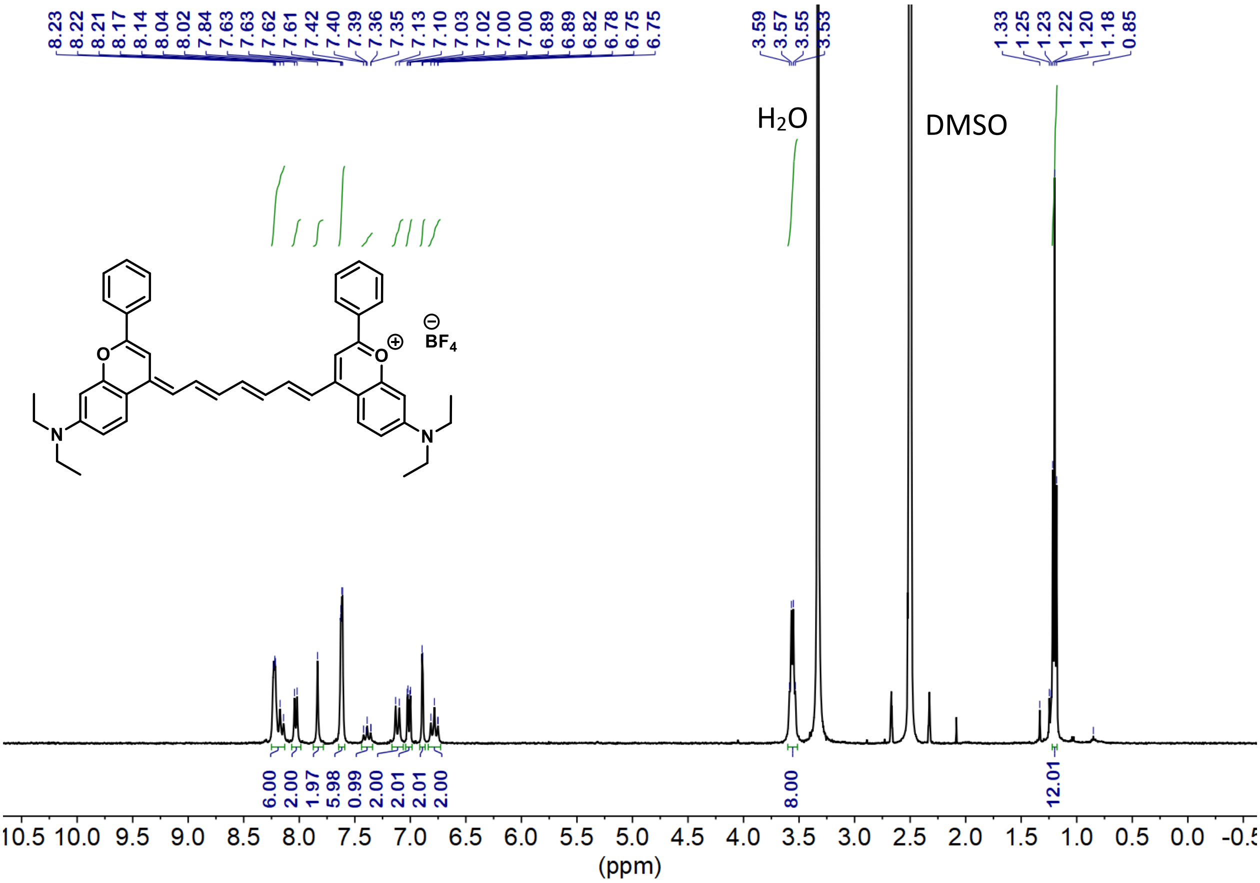

Figure S19. ^{1}H NMR (400 MHz, DMSO-d_6): **1H**

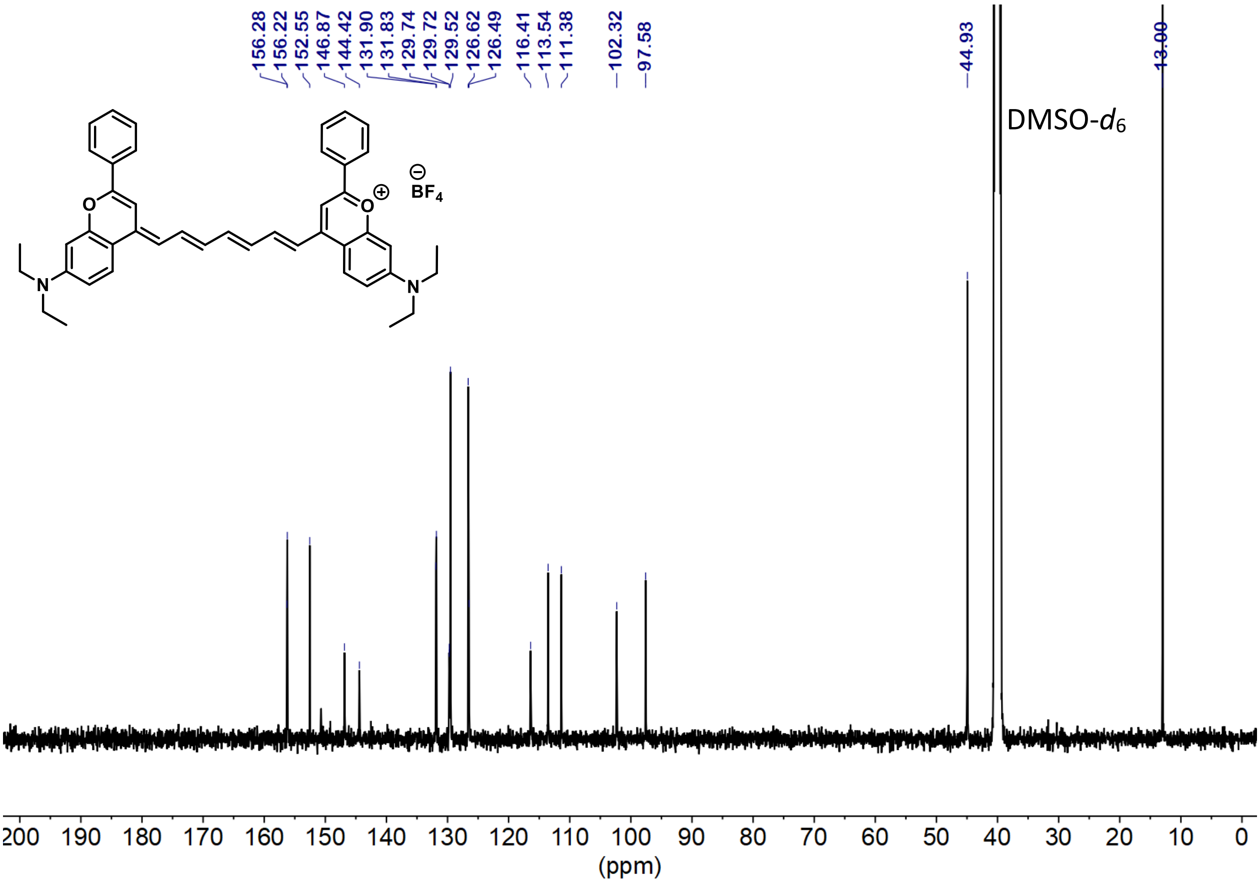

Figure S20. ^{13}C{^{1}H} NMR (126 MHz, DMSO-d_6): **1H**

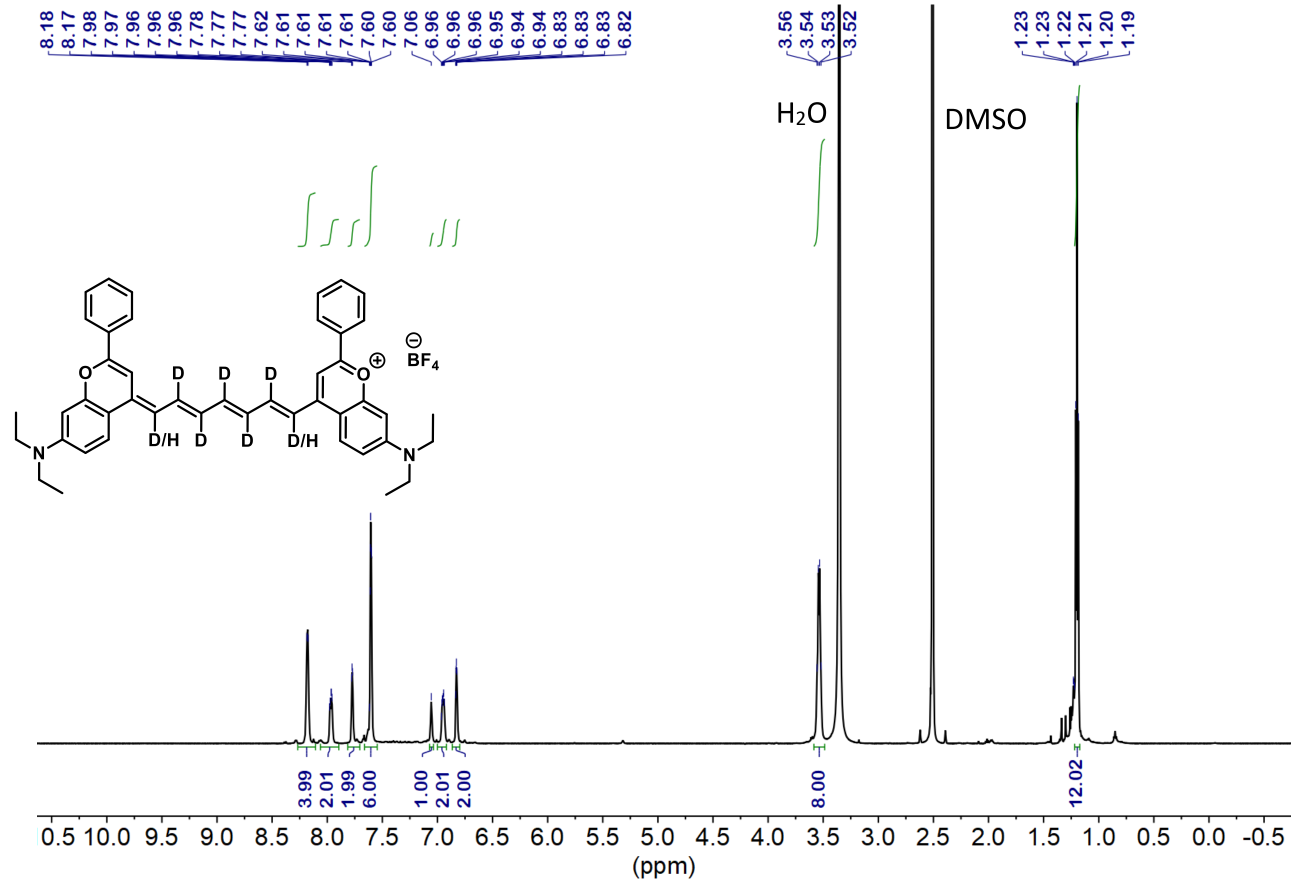

Figure S21. ^{1}H NMR (600 MHz, DMSO-d_6): **1D**

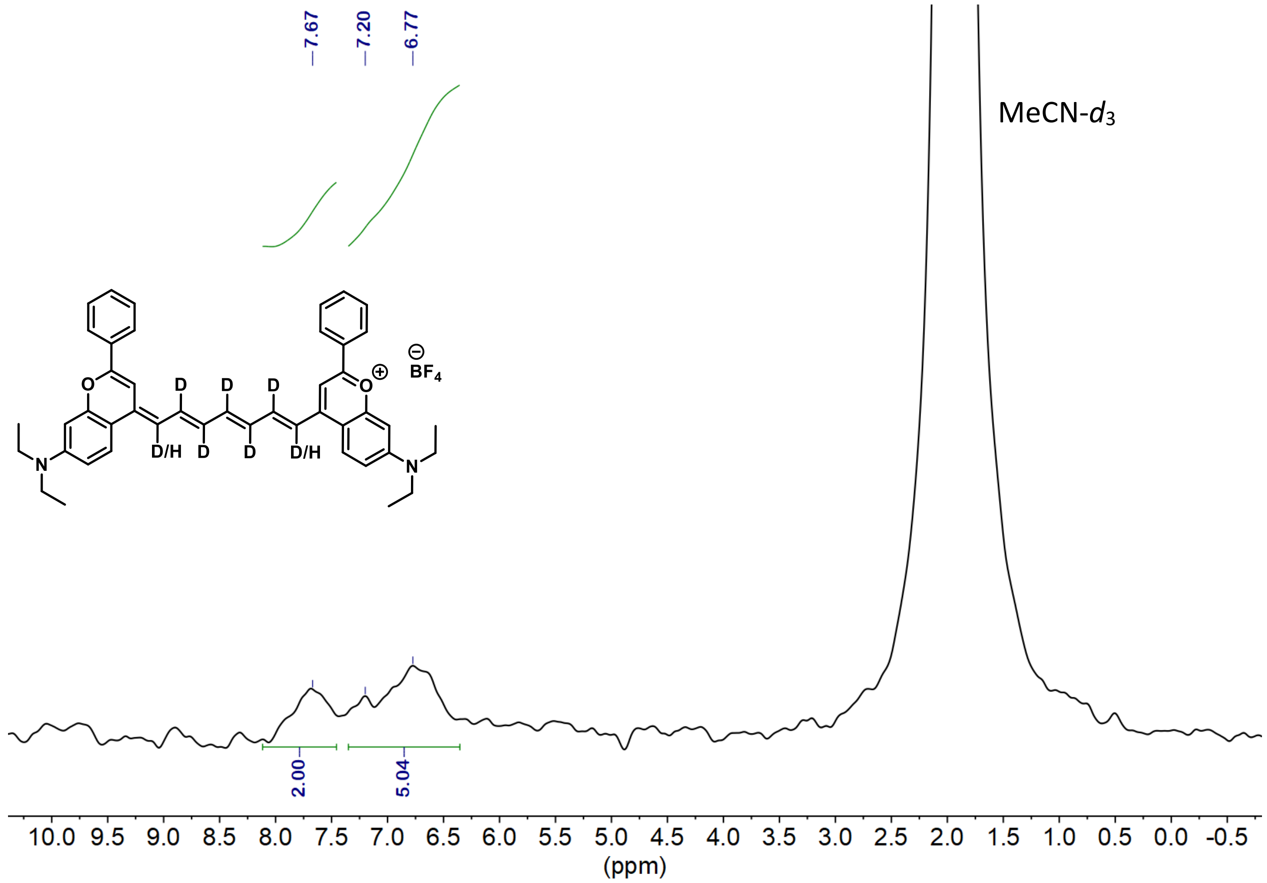

Figure S22. ^{2}H NMR (92 MHz, MeCN): **1D**

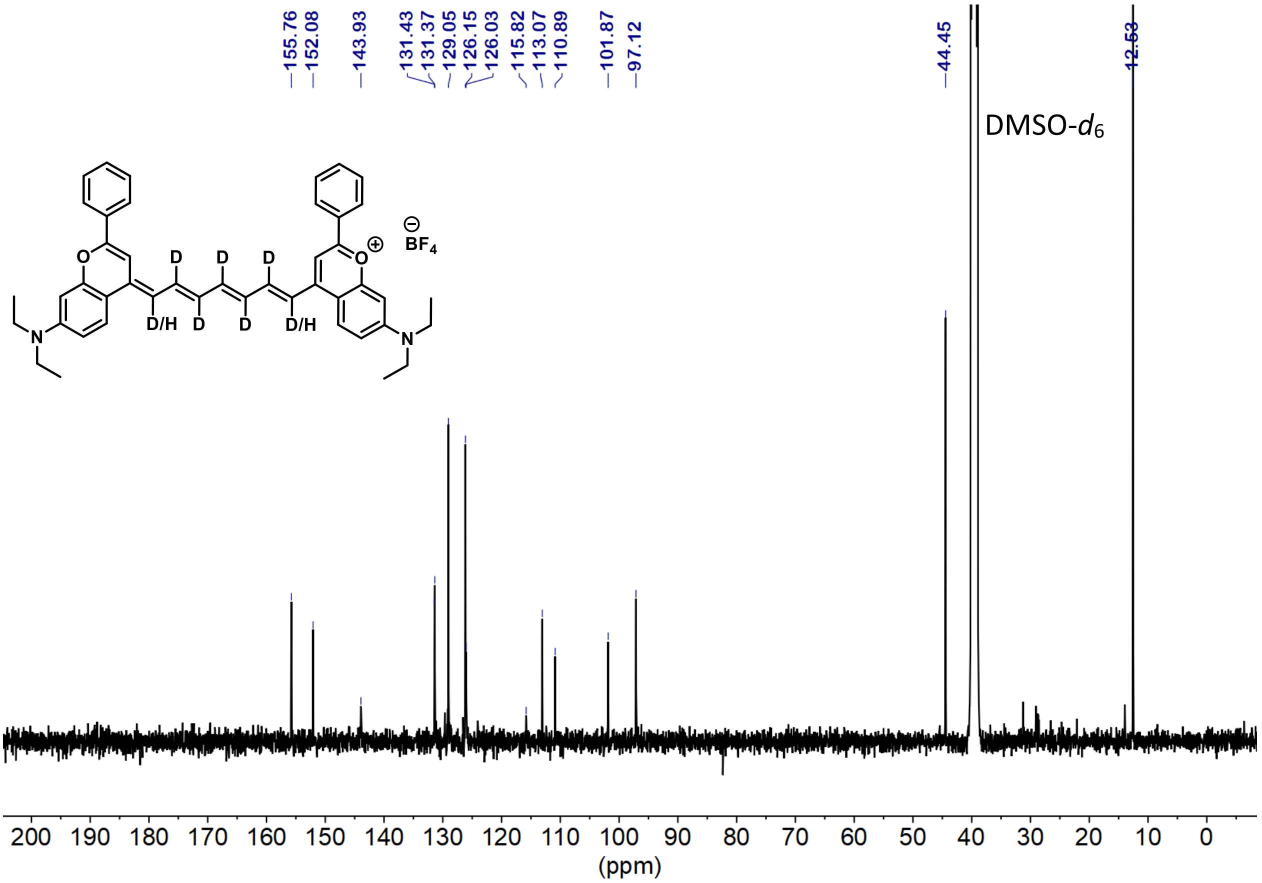

Figure S23. $^{13}C\{^1H\}$ NMR (126 MHz, DMSO-d_6): **1D**

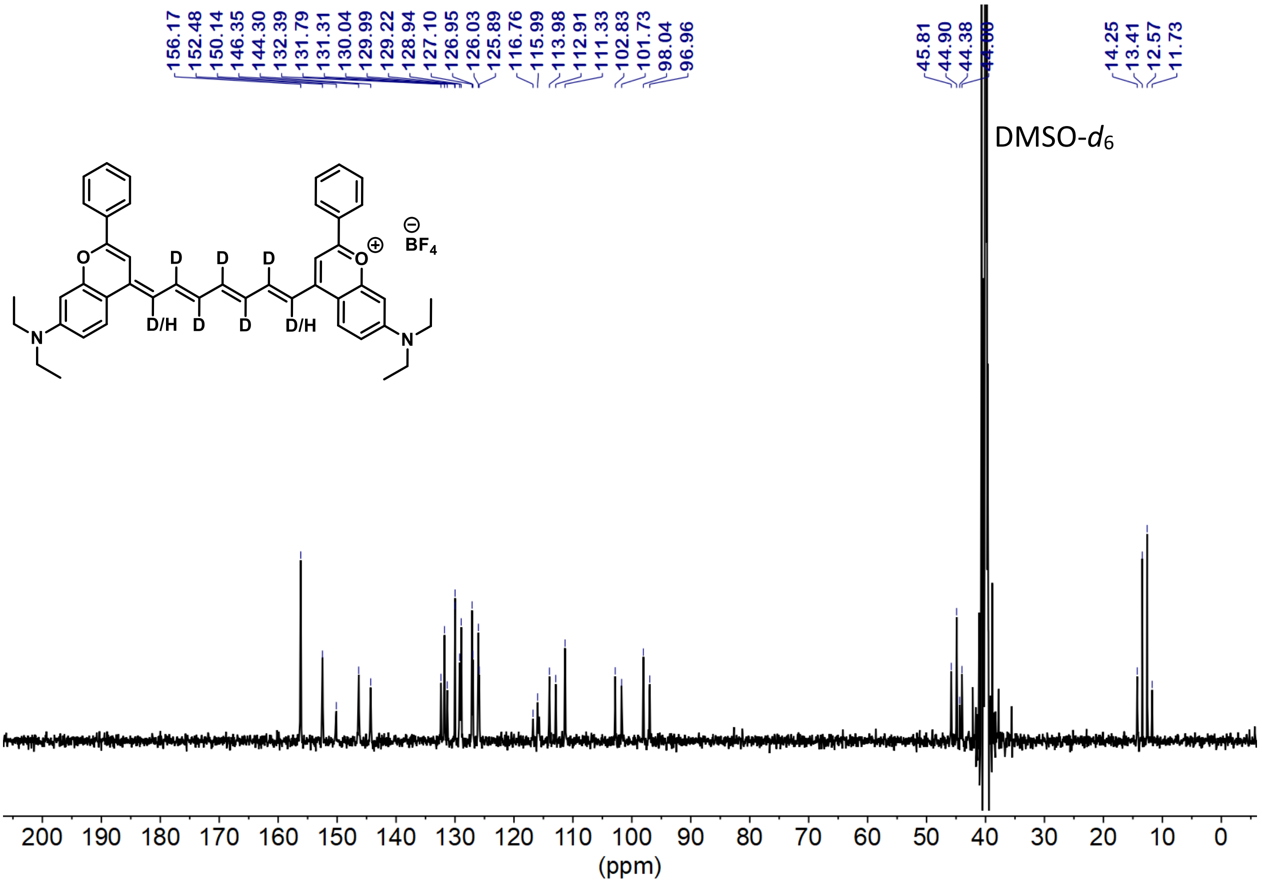

Figure S24. $^{13}C\{^2H\}$ NMR (151 MHz, DMSO-d_6): **1D**

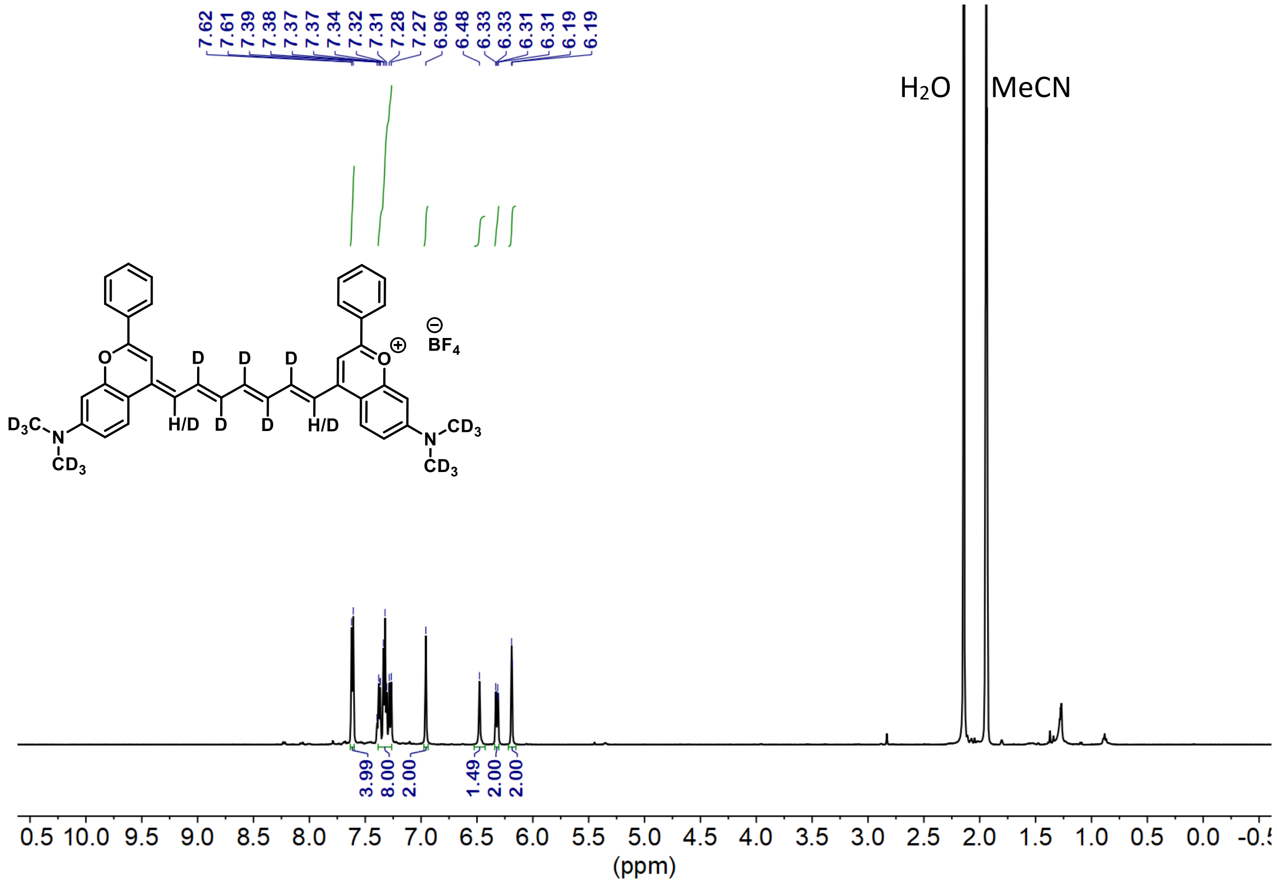

Figure S25. ^{1}H NMR (500 MHz, MeCN-d_3): **1DD**

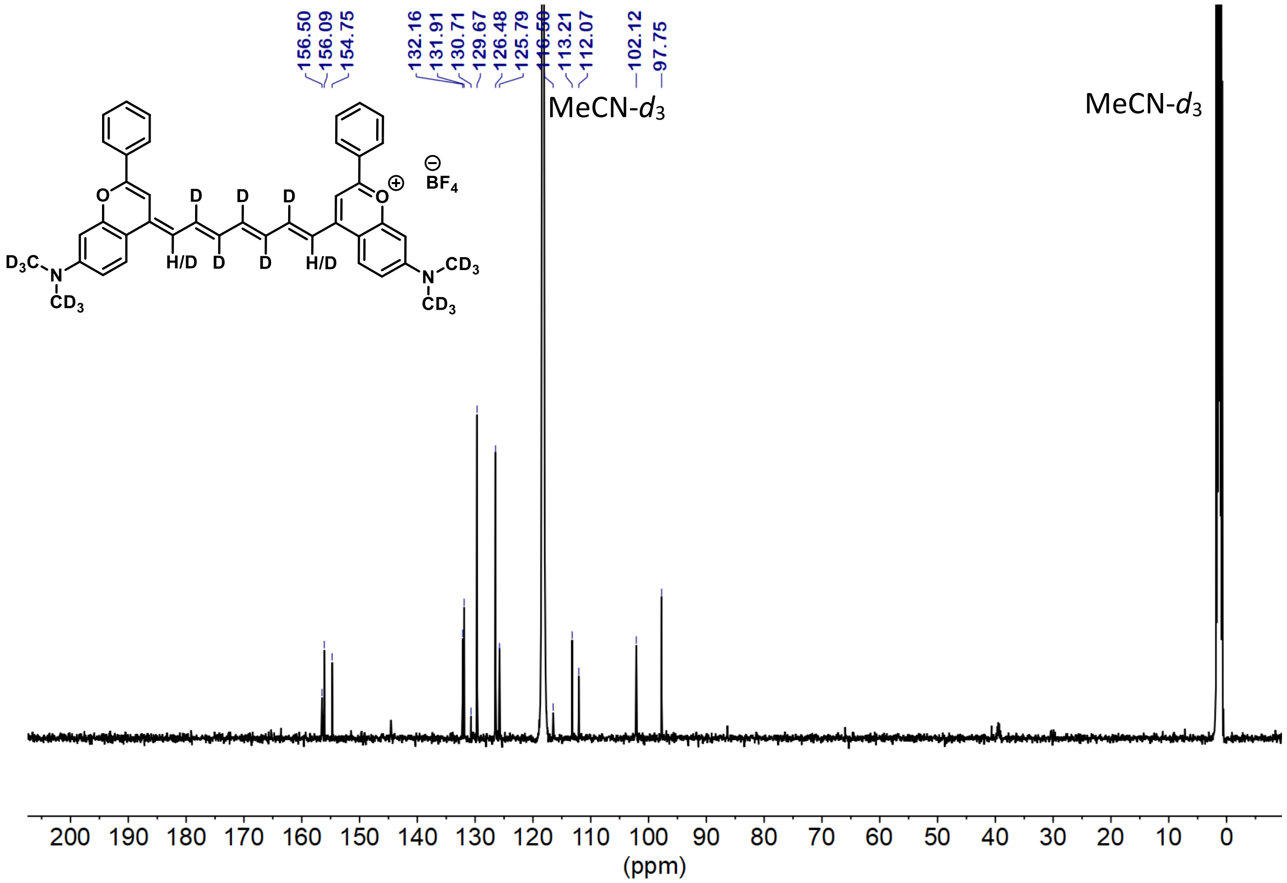

Figure S26. $^{13}C\{^{1}H\}$ NMR (126 MHz, MeCN-d_3): **1DD**

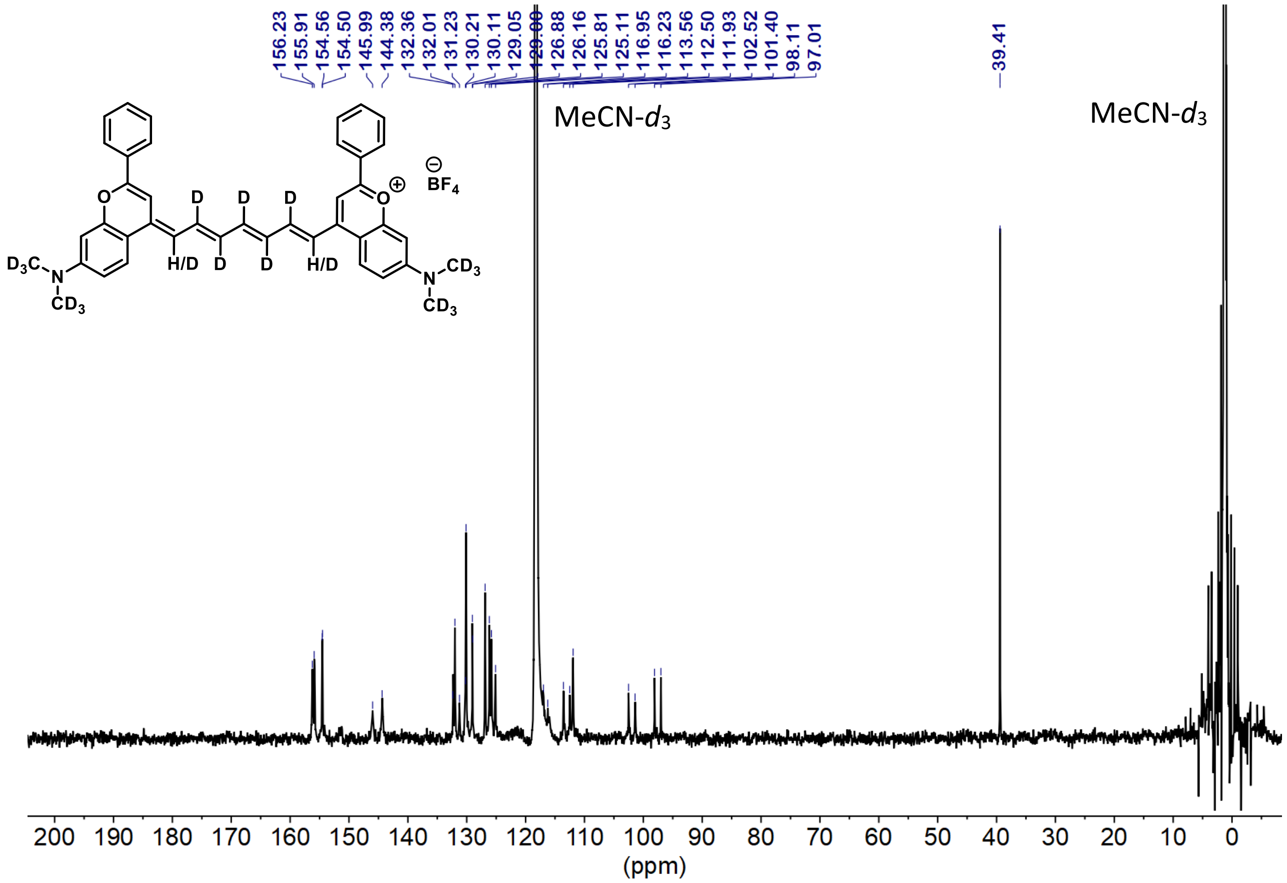

Figure S27. $^{13}C\{^{2}H\}$ NMR (151 MHz, MeCN-d_3): **1DD**

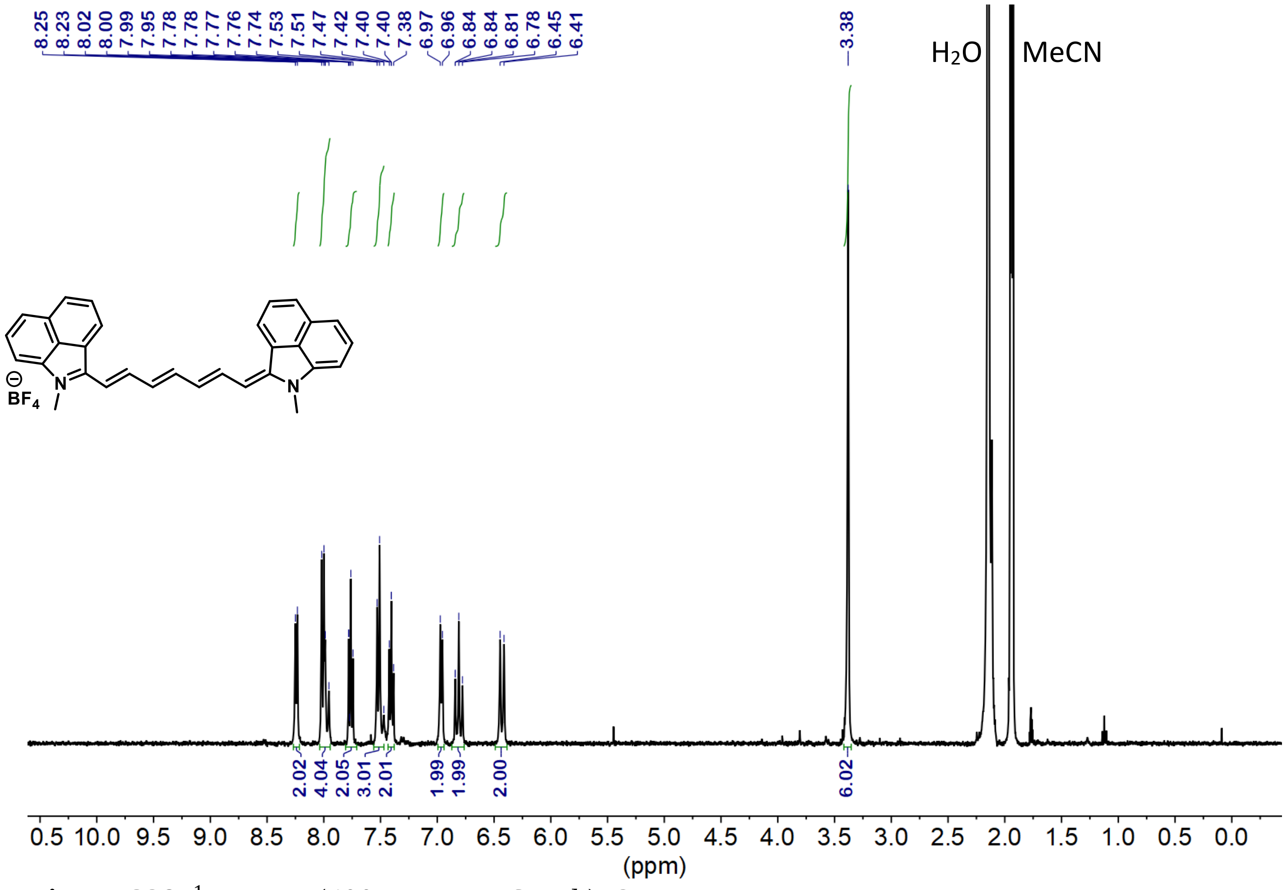

Figure S28. ^{1}H NMR (400 MHz, MeCN-d_3): **2H**

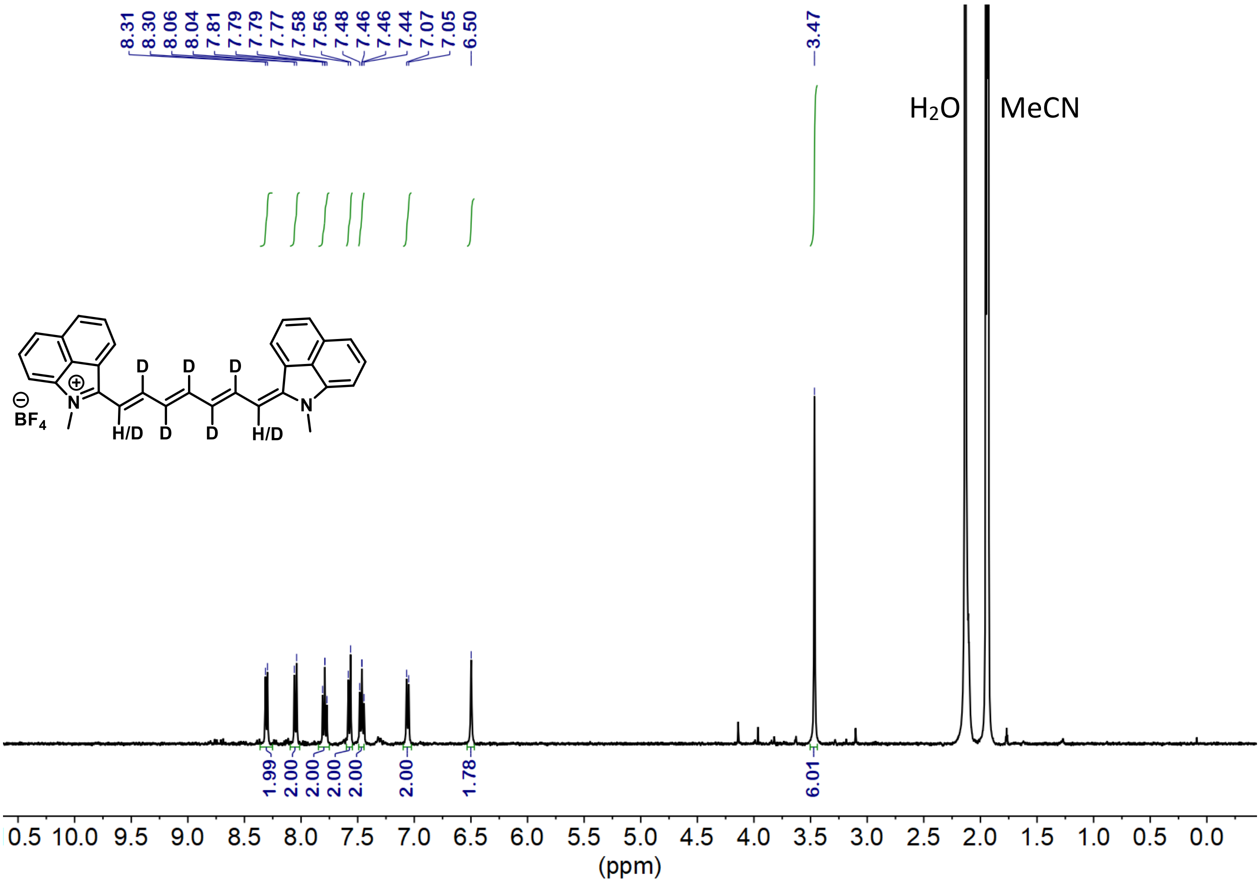

Figure S29. ^{1}H NMR (400 MHz, MeCN-d_3): **2D**

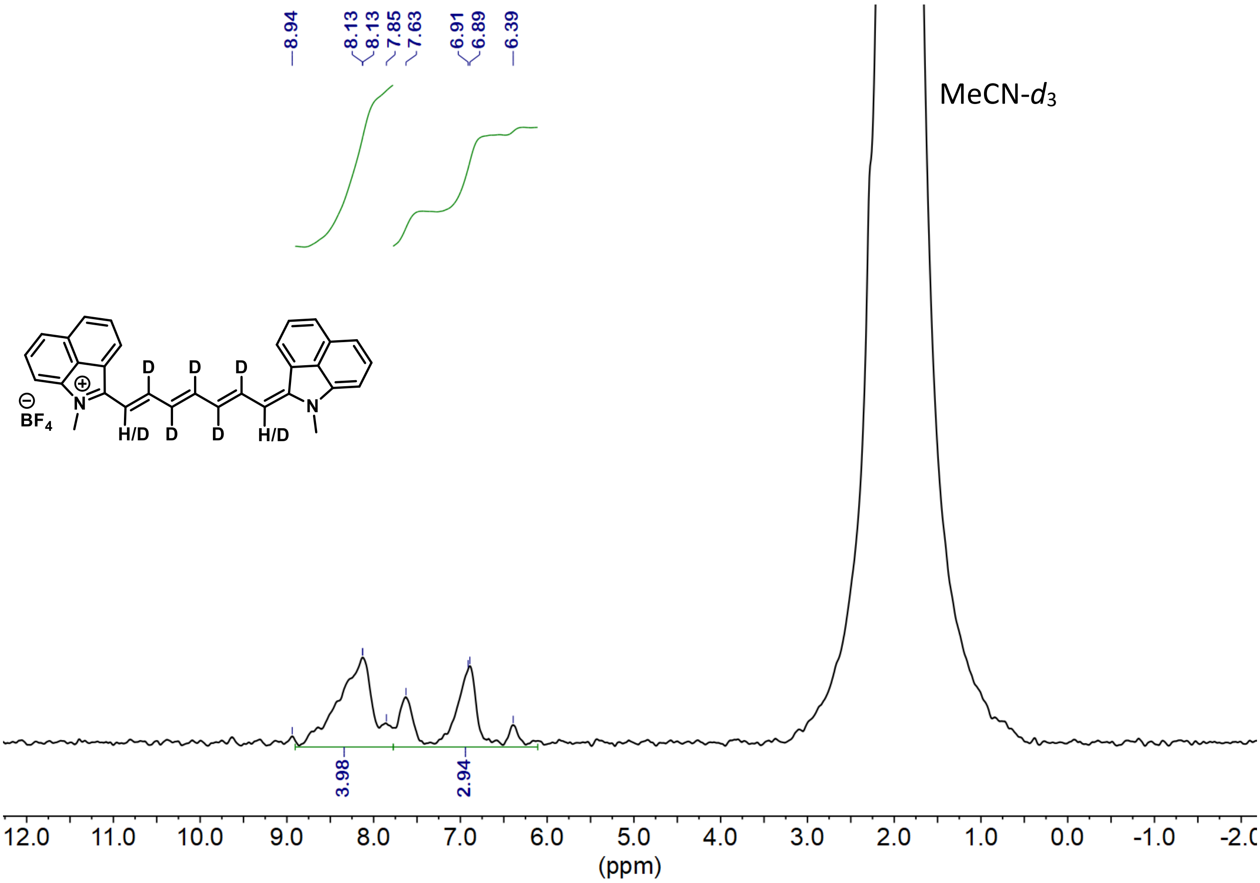

Figure S30. ^{2}H NMR (92 MHz, MeCN): **2D**

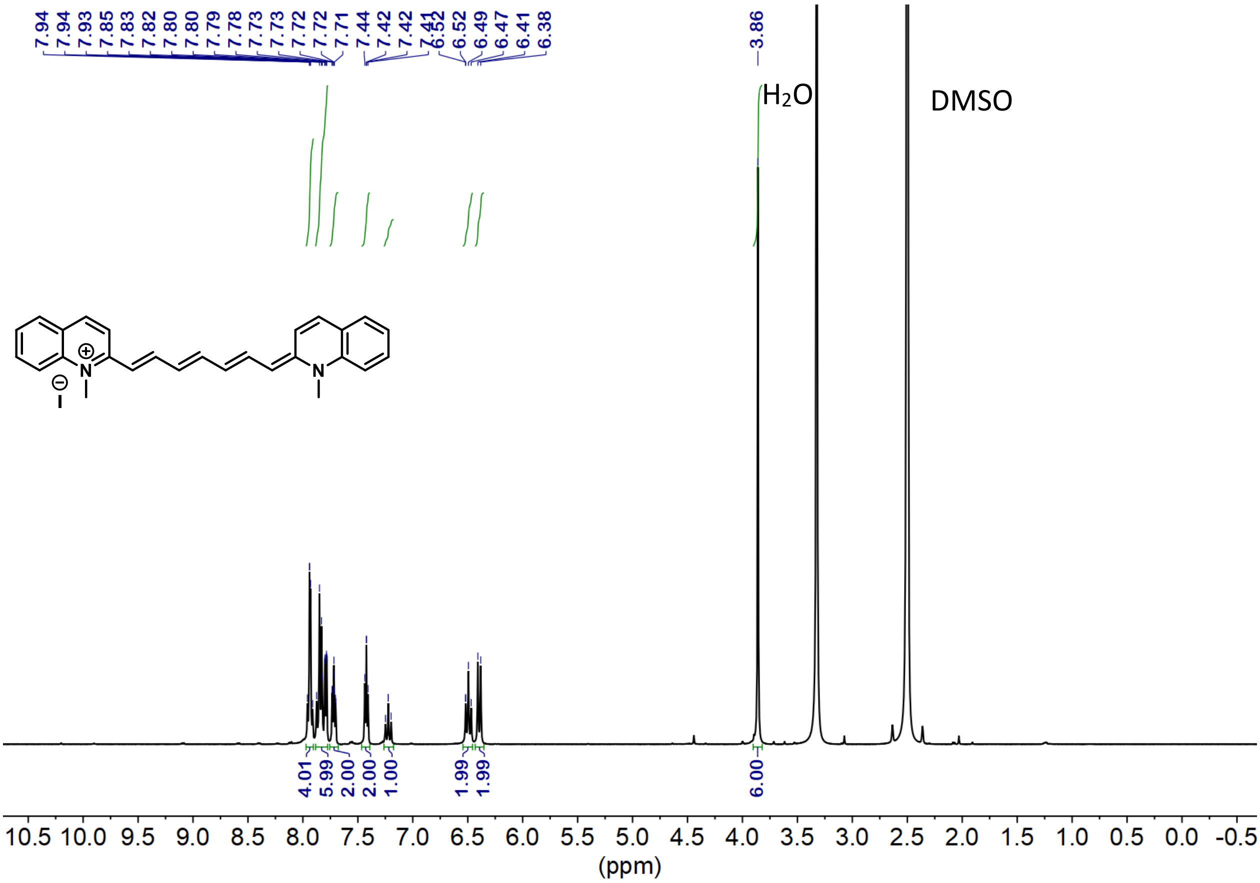

Figure S31. ^{1}H NMR (500 MHz, DMSO-d_6): **3H**

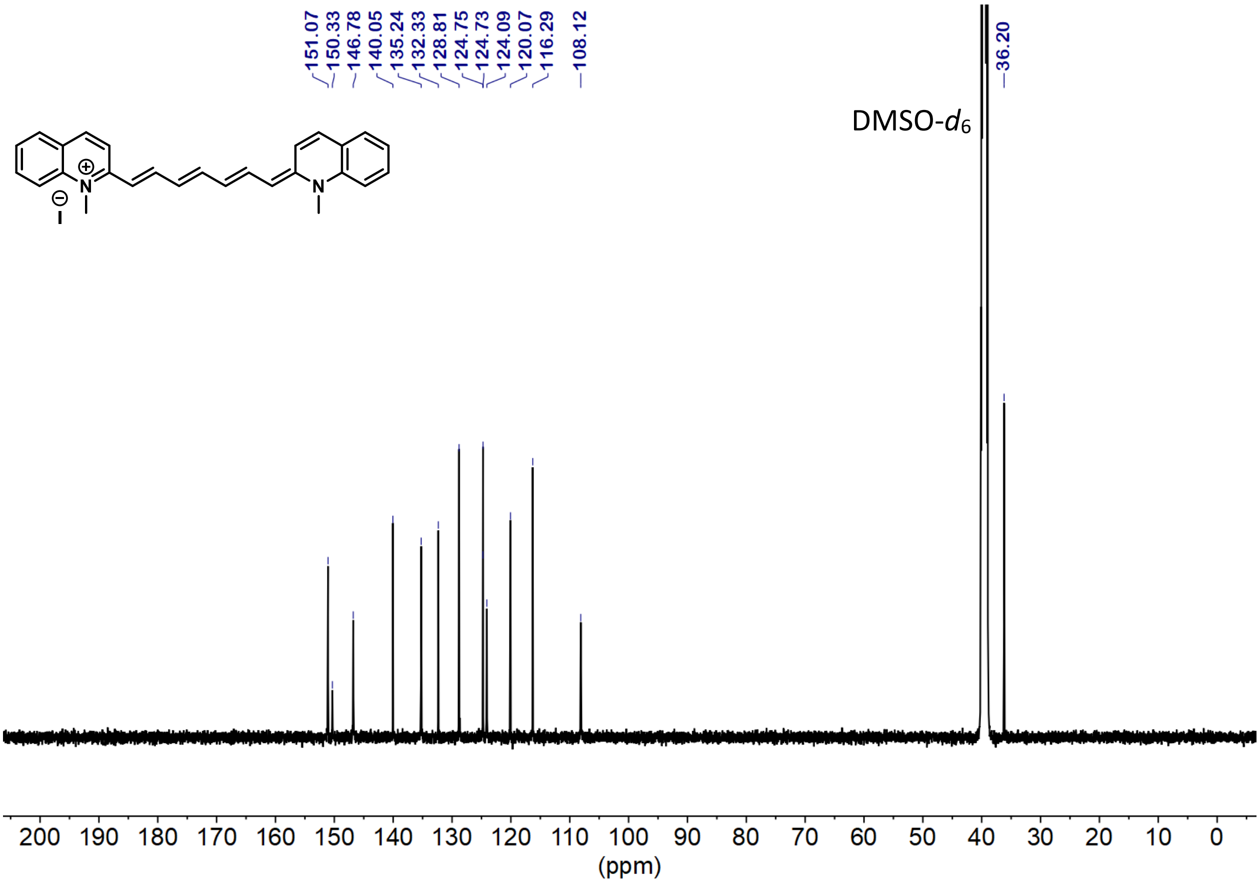

Figure S32. $^{13}C\{^{1}H\}$ NMR (126 MHz, DMSO-d_6): **3H**

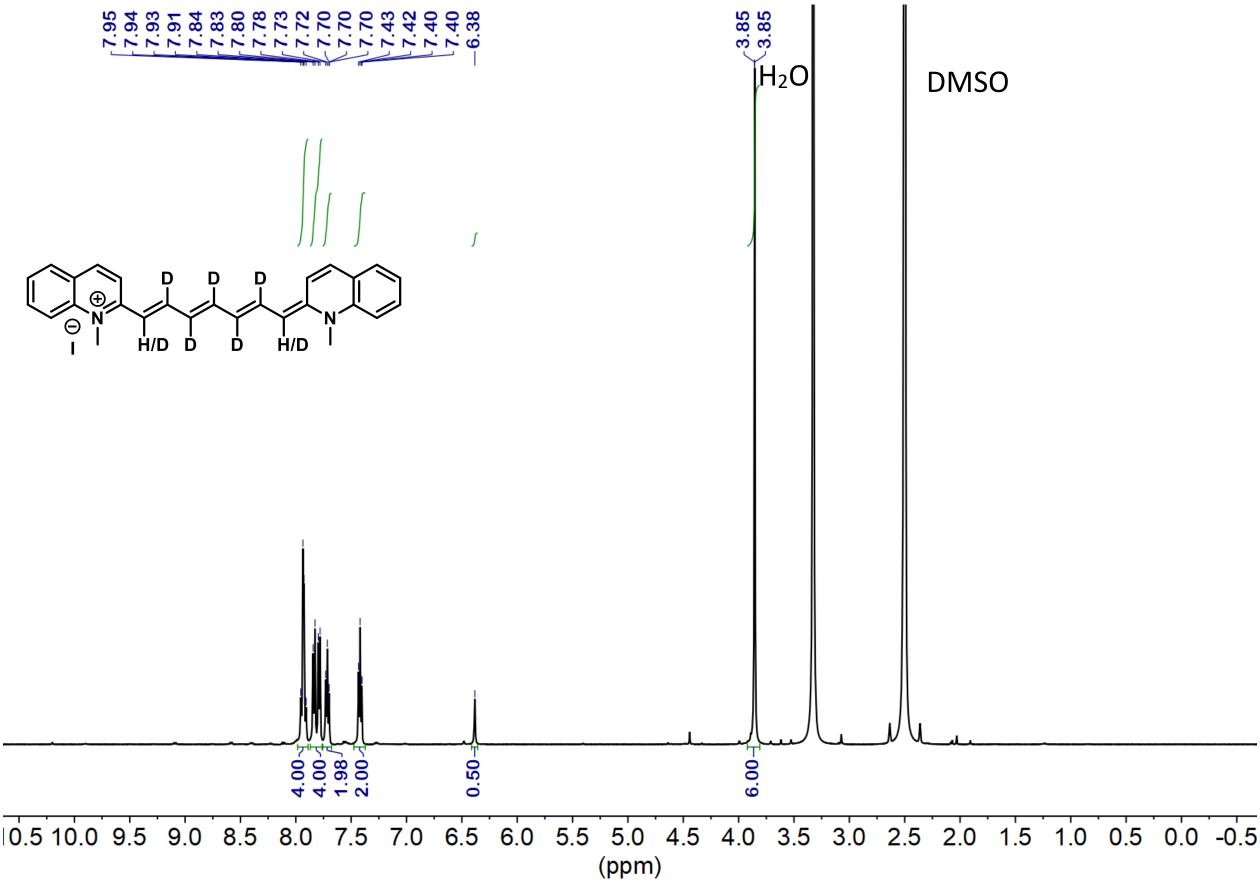

Figure S33. ^{1}H NMR (500 MHz, DMSO-d_6): **3D**

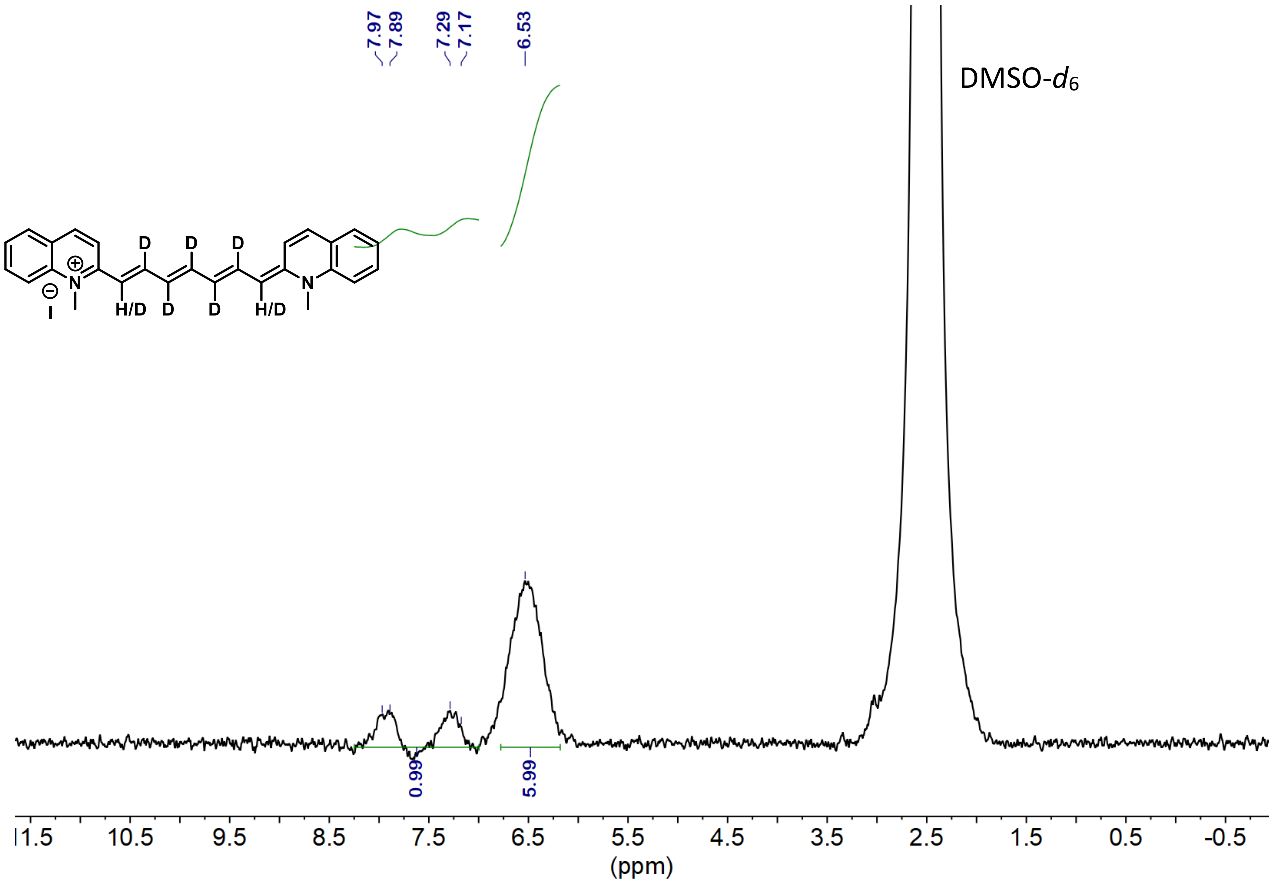

Figure S34. ^{2}H NMR (92 MHz, DMSO): **3D**

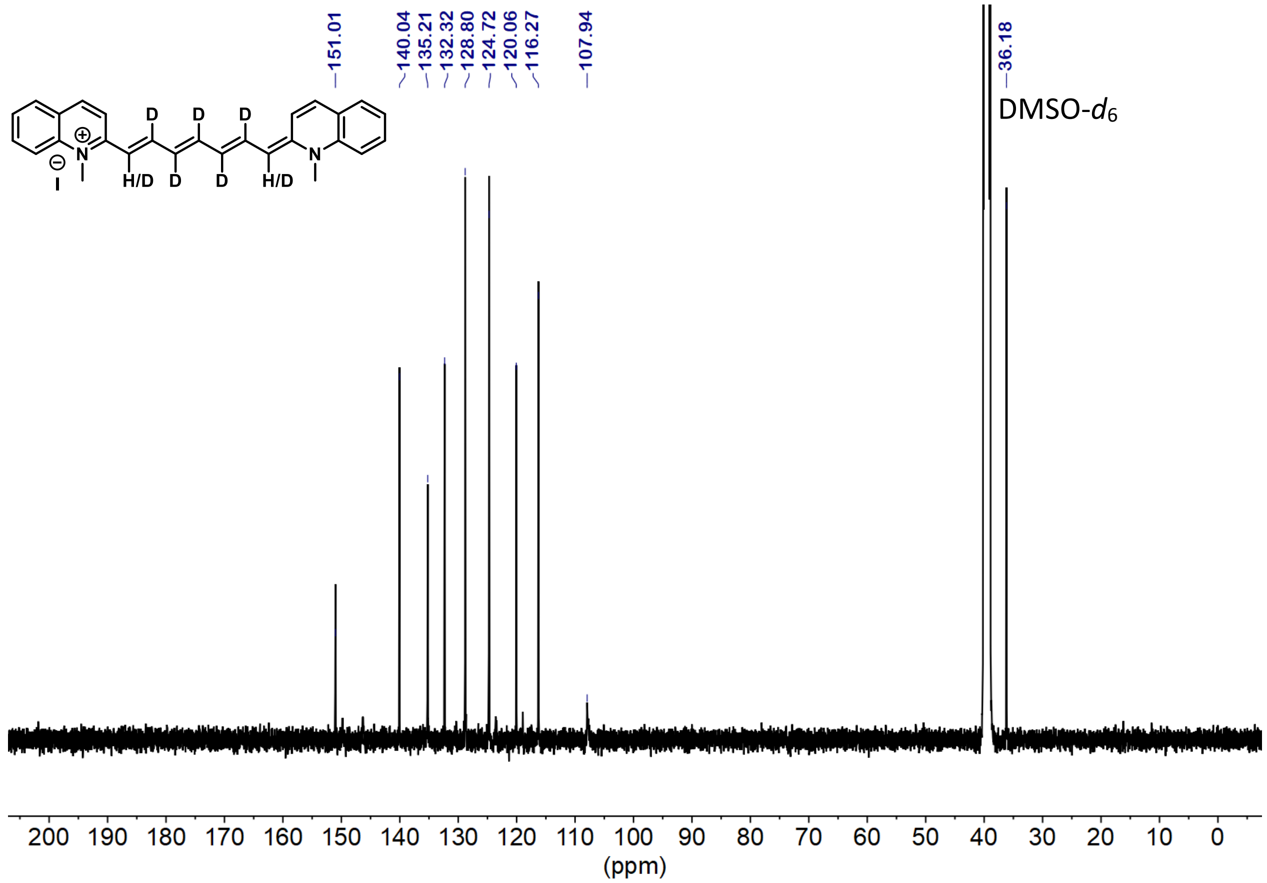

Figure S35. $^{13}C\{^{1}H\}$ NMR (126 MHz, DMSO-d_6): **3D**

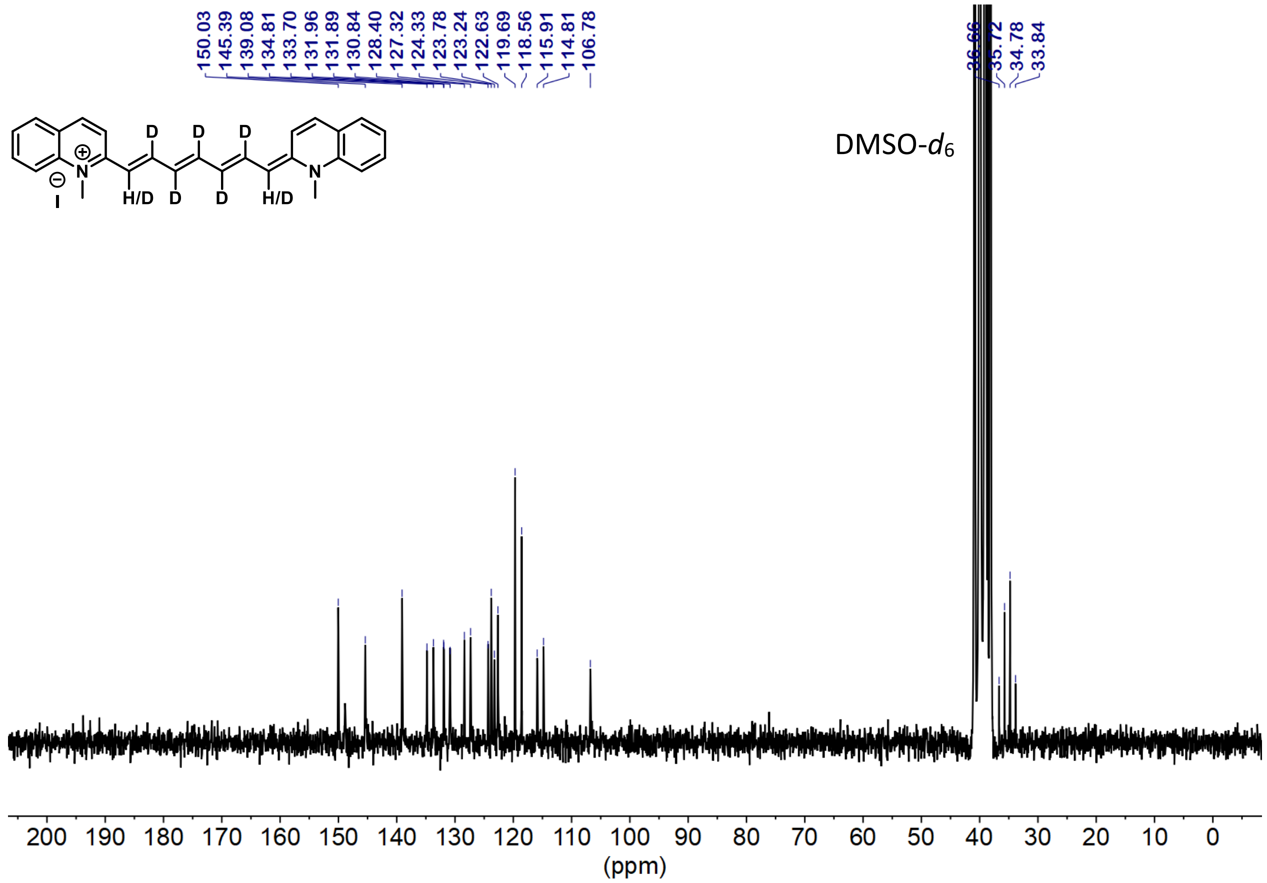

Figure S36. $^{13}C\{^{2}H\}$ NMR (151 MHz, DMSO-d_6): **3D**

HRMS Spectroscopy

Calculation the Relative ratio of d_5:d_6:d_7 Cyanines

The relative ratio of d_5:d_6:d_7 for the final cyanines **1–4D** was calculated from isotope pattern of the molecular peak detected by HRMS. Based on the relative abundance of the respective masses compared to the expected isotopic pattern ratios for each of the derivatives d_5 – d_7 the relative ratio was calculated. The d_5 isomers were taken as a referential starting point due to their specific main peak (100% abundance) not coinciding with other isotopic values. Using **1H-D** as an example, the intensity of mass corresponding to d_5 experiences contribution by the *d5*-isomer:

$[d_5] = I(\mathrm{M})$, where M corresponds to the calculated exact mass of d_5-isomer

The mass corresponding to d_6-**1D** experiences contribution from d_6-**1D** and the isotope pattern of d_5-**1D**, which can be calculated from the molecular formula (e.g. in ChemDraw):

$[d_6] + [d_5] \times 0.487 = I(\mathrm{M}+1)$, where M+1 corresponds to the calculated exact mass of the d_6-isomer.

The mass corresponding to d_7-**1D** experiences contribution from d_7-**1D** and the isotope patterns of d_6- and d_5-**1D**, which can be calculated from the molecular formula (e.g. in ChemDraw):

$[d_7] + [d_6] \times 0.487 + [d_5] \times 0.116 = I(\mathrm{M}+2)$, where M+2 corresponds to the calculated exact mass of the d_7-isomer.

Solving these three equations and normalizing the sum of $[d_7] + [d_6] + [d_5]$ to 100 gives the ratio of the three isomers in percent.

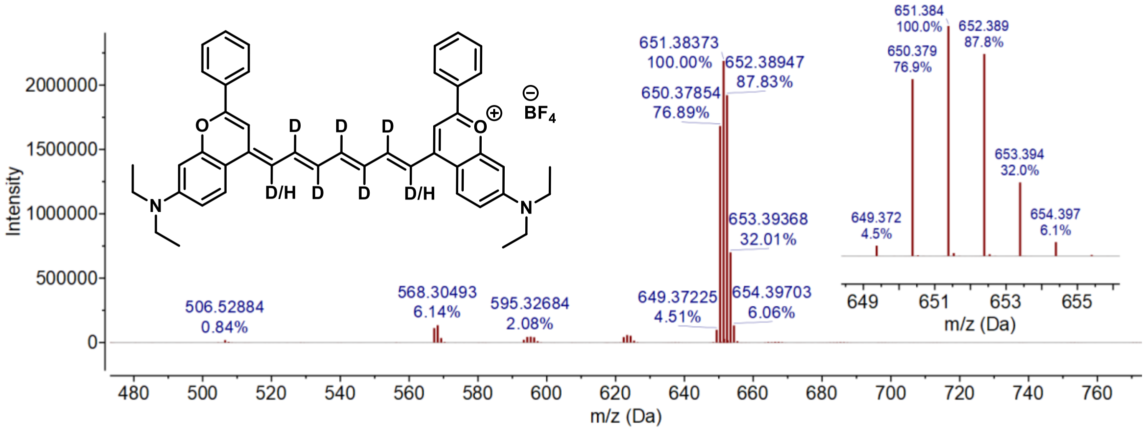

Figure S37. HRMS (ESI+): **1D**

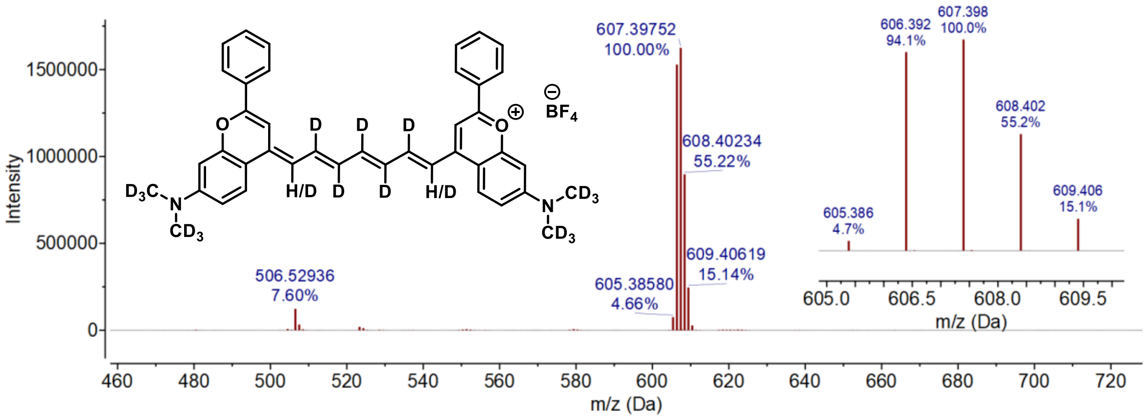

Figure S38. HRMS (ESI+): **1DD**

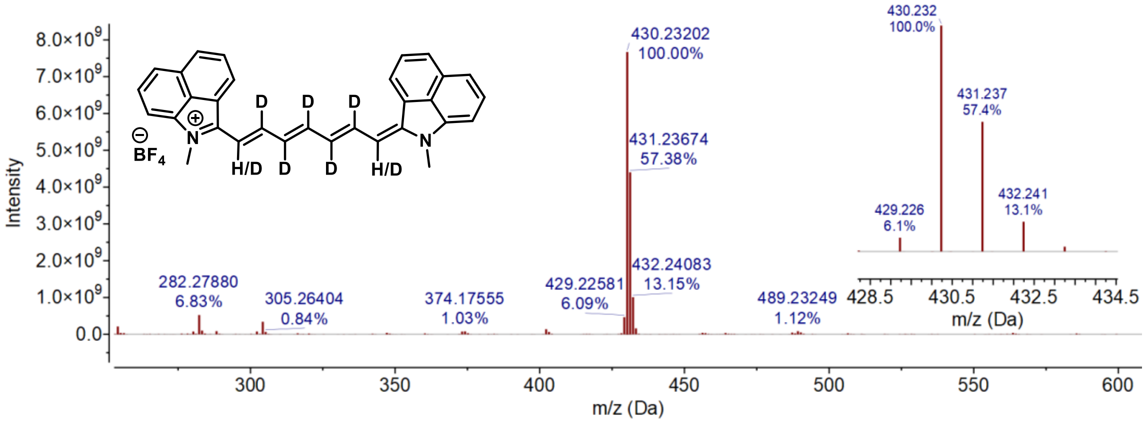

Figure S39. HRMS (ESI+): **2D**

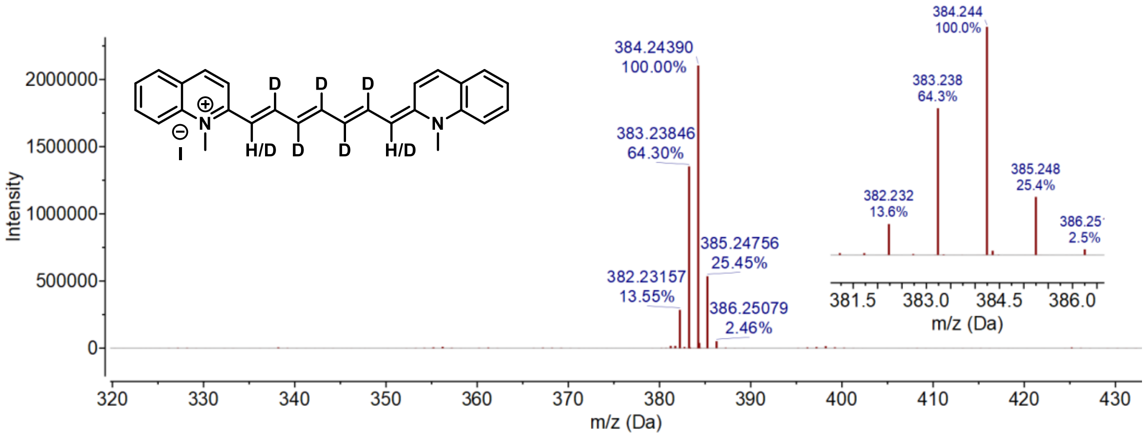

Figure S40. HRMS (ESI+): **3D**

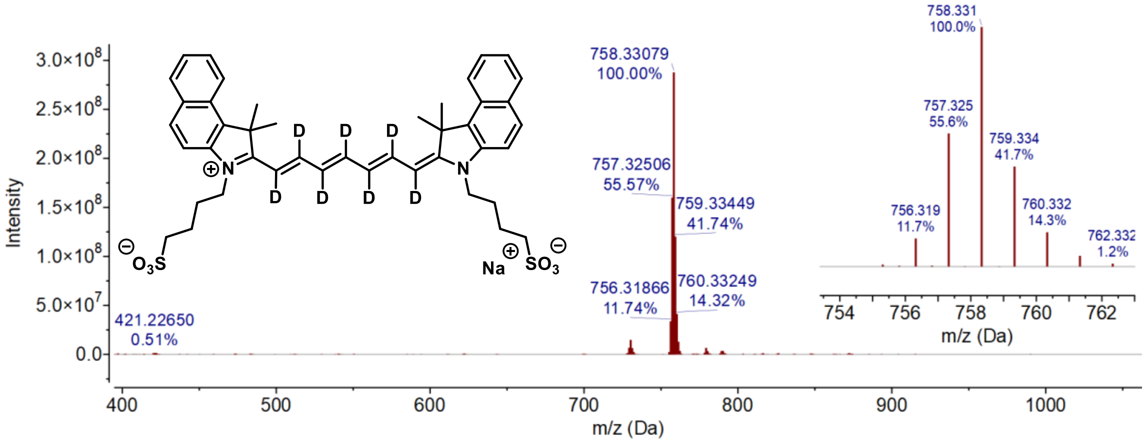

Figure S41. HRMS (ESI+): **4D**

UV-Vis Absorption and Emission Spectroscopy of 1–4H and 1–4D.

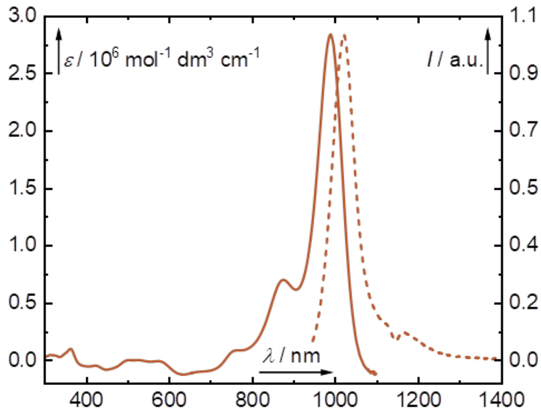

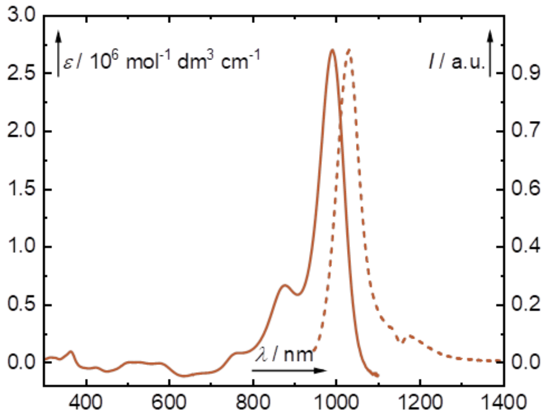

Figure S42. UV-Vis absorption (solid) and emission (dashed) spectra of **1H** (left) and **1D** (right) in DCM.

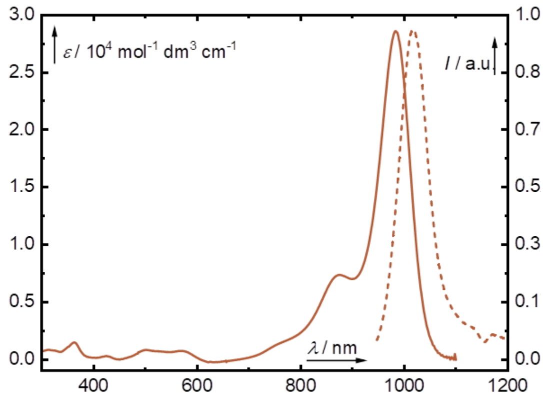

Figure S43. UV-Vis absorption (solid) and emission (dashed) spectra of **1DD** in DCM.

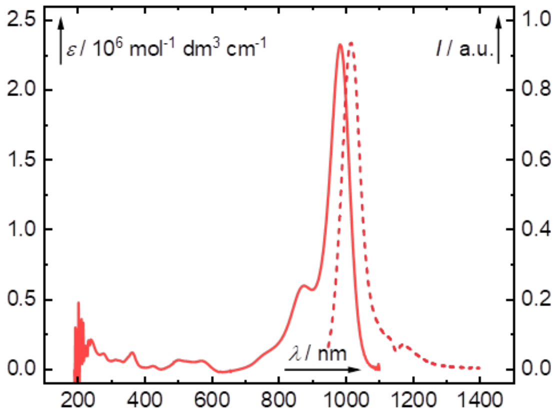

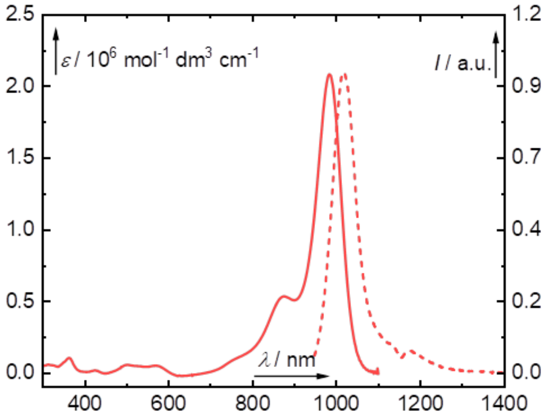

Figure S44. UV-Vis absorption (solid) and emission (dashed) spectra of **2H** (left) and **2D** (right) in DCM.

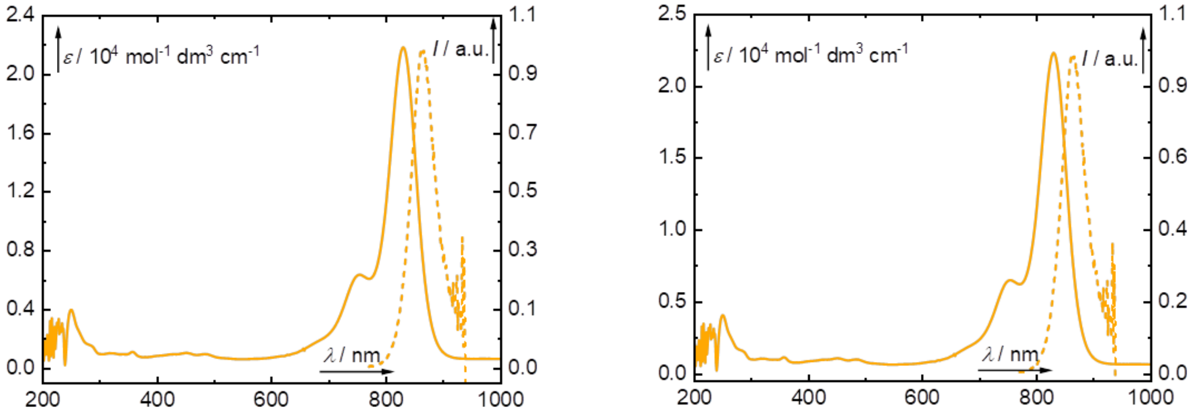

Figure S45. UV-Vis absorption (solid) and emission (dashed) spectra of **3H** (left) and **3D** (right) in DCM.

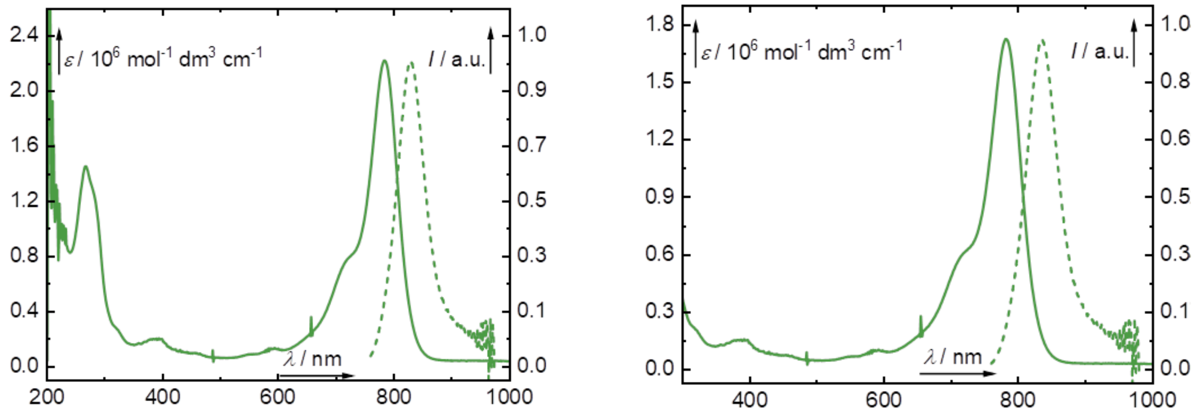

Figure S46. UV-Vis absorption (solid) and emission (dashed) spectra of **4H** (left) and **4D** (right) in DCM.

Absorption Coefficients of 1–4H and 1–4D.

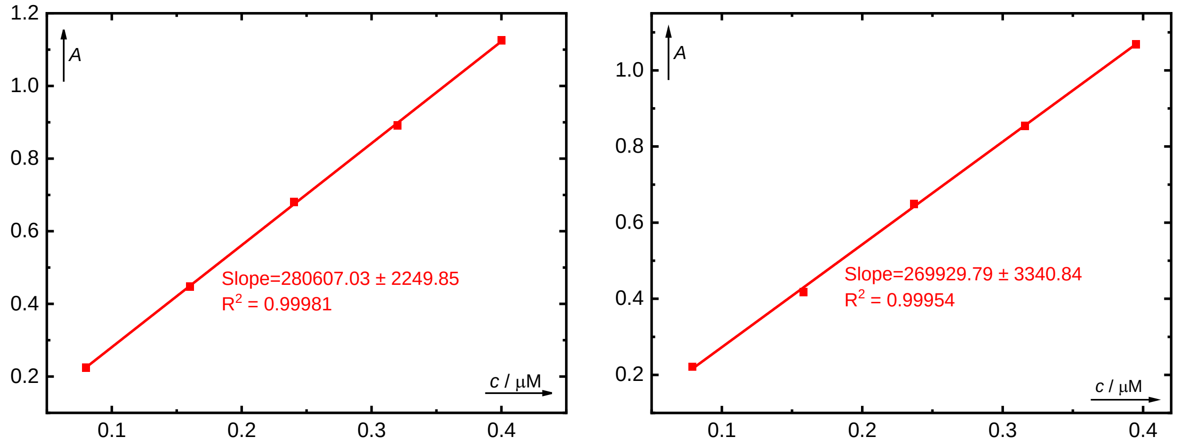

Figure S47. Dependence of absorption at λ_{max} on the concentration of **1H** (left) and **1D** (right) in DCM.

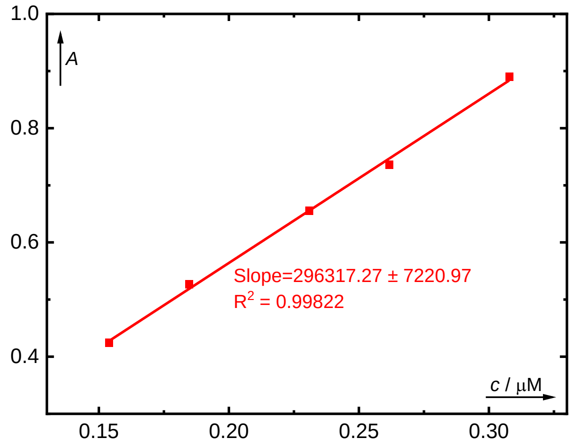

Figure S48. Dependence of absorption at λ_{max} on the concentration of **1DD** in DCM.

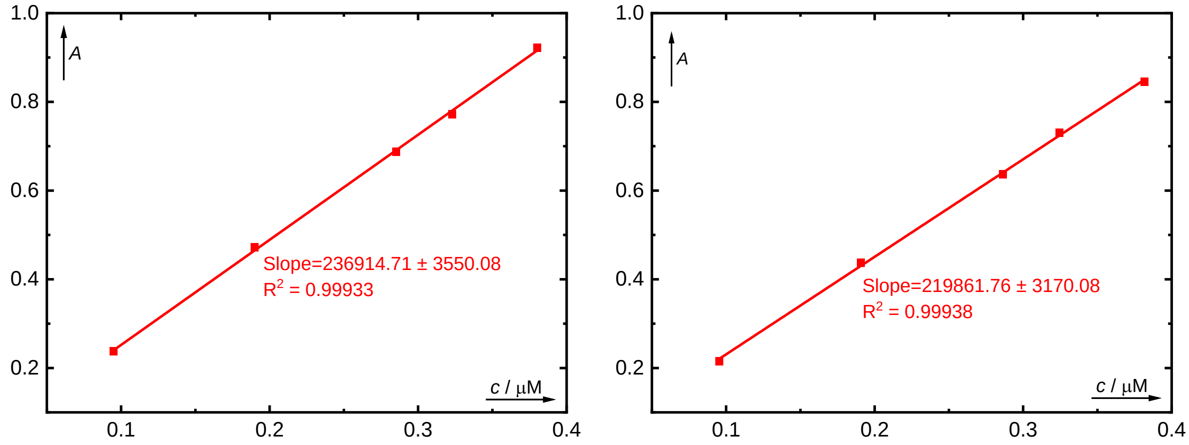

Figure S49. Dependence of absorption at λ_{max} on the concentration of **2H** (left) and **2D** (right) in DCM.

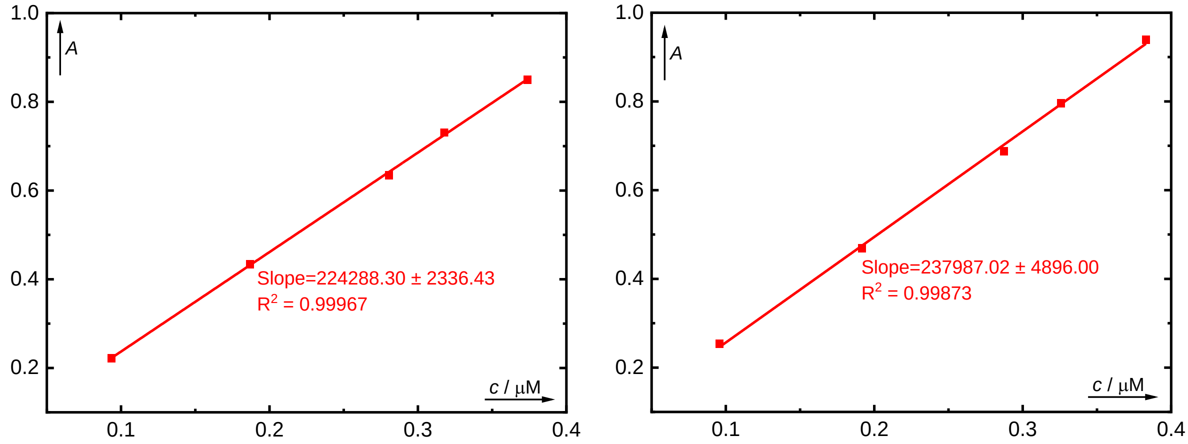

Figure S50. Dependence of absorption at λ_{max} on the concentration of **3H** (left) and **3D** (right) in DCM.

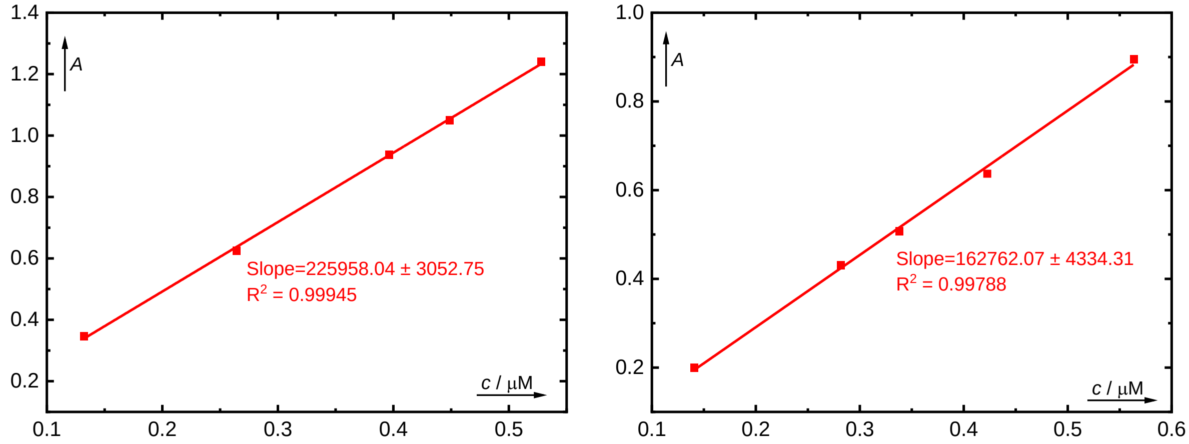

Figure S51. Dependence of absorption at λ_{max} on the concentration of **4H** (left) and **4D** (right) in MeOH.

Quantum Yields of Fluorescence of 1–4H and 1–4D.

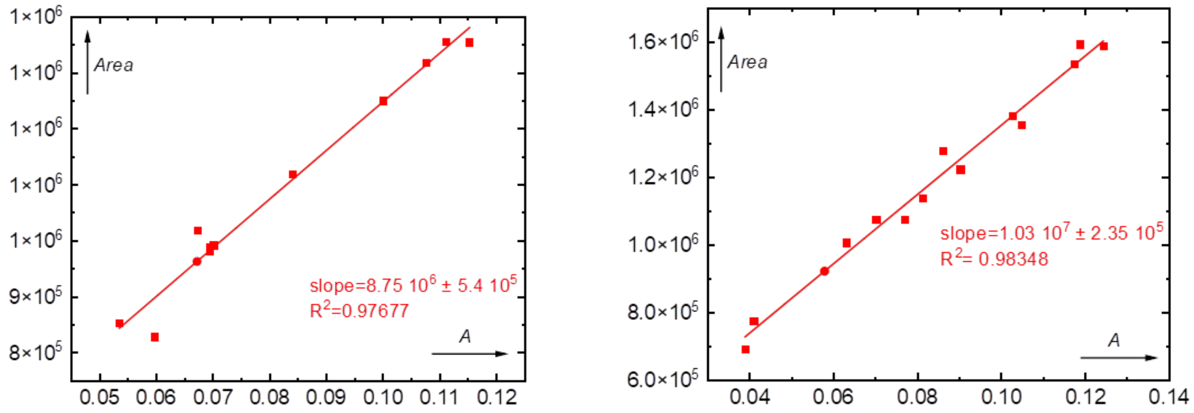

Figure S52. Dependence of the integral of emission on the absorption at λ_{exc} nm for **1H** (left) and **1D** (right) in DCM.

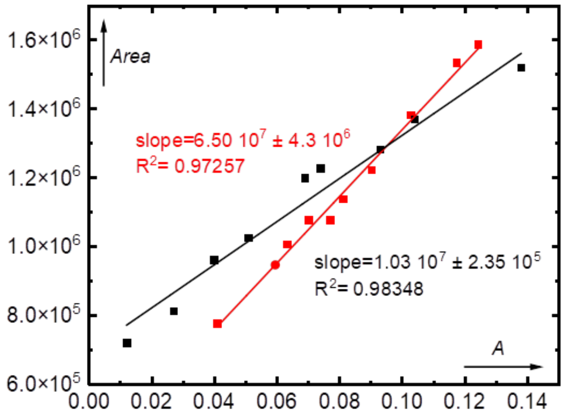

Figure S53. Dependence of the integral of emission on the absorption at λ_{exc} nm for **1H** in DCM (depicted in red) and DMSO (depicted in black).

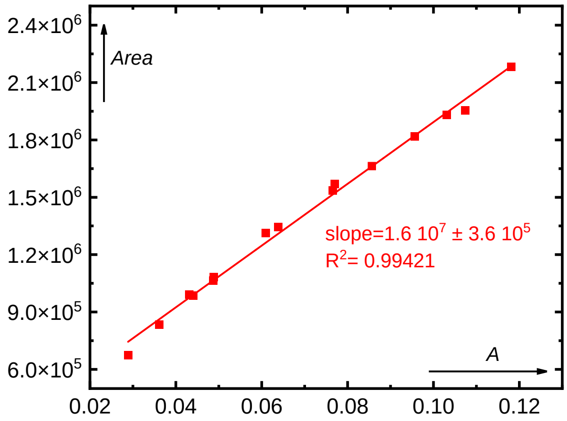

Figure S54. Dependence of the integral of emission on the absorption at λ_{exc} nm for **1DD** in DCM.

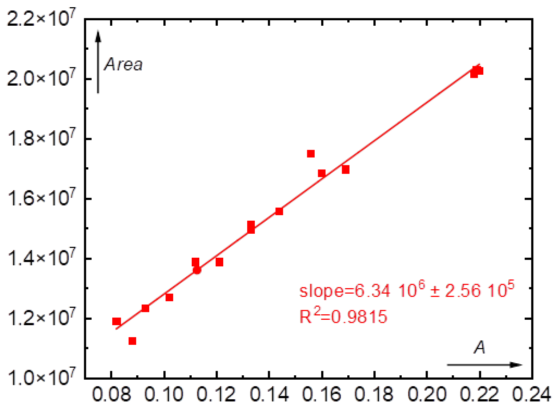

Figure S55. Dependence of the integral of emission on the absorption at λ_{exc} nm for **2H** (left) and **2D** (right) in DCM.

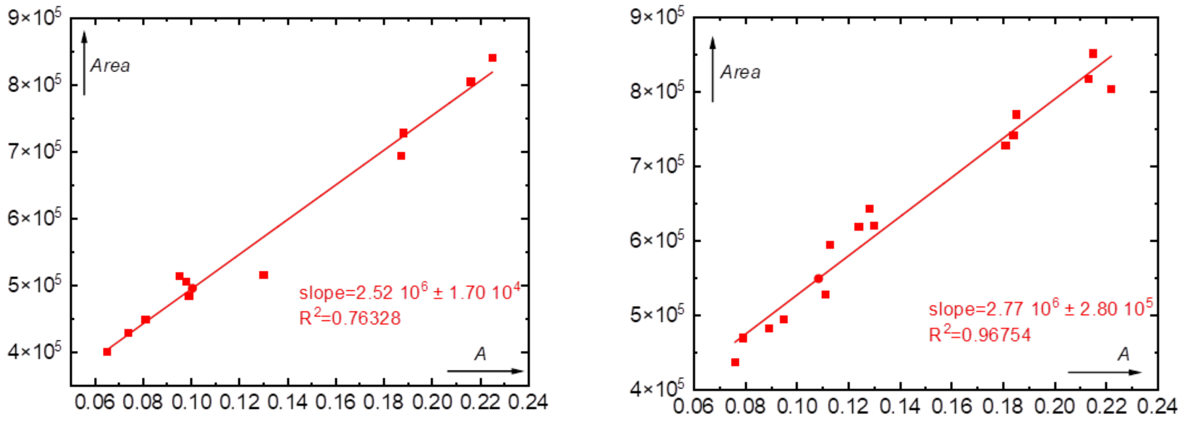

Figure S56. Dependence of the integral of emission on the absorption at λ_{exc} nm for **3H** (left) and **3D** (right) in DCM.

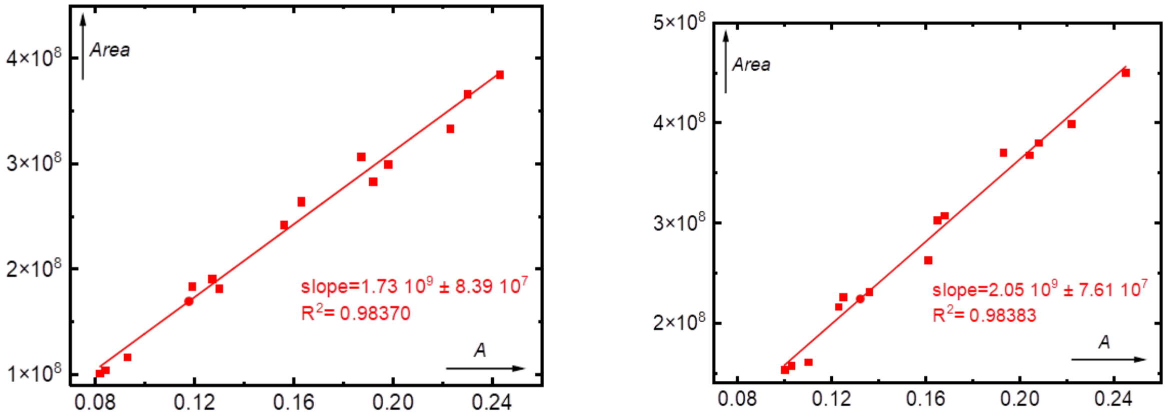

Figure S57. Dependence of absorption at λ= 750 nm on the area of emission of **4H** (left) and **4D** (right) in DCM.

Deuterium-Exchange Experiments

The degree of deuteration at C1′ and C7′ positions is related to the ability of the heterocycle to undergo deuterium exchange at the activated methyl under the reaction conditions. Premixing heterocycle **10** in d_4-CD_3OD led to no discernable deuterium enrichment at the methyl after 1 hour (<5%), presumably due to low acidity of the hydrogens (Fig. S57). Addition of Et_3N or premixing with AcONa in case of heterocycles **10** and **13**, respectively, did not increase deuterium incorporation in the final cyanines **1D** and **2D**.

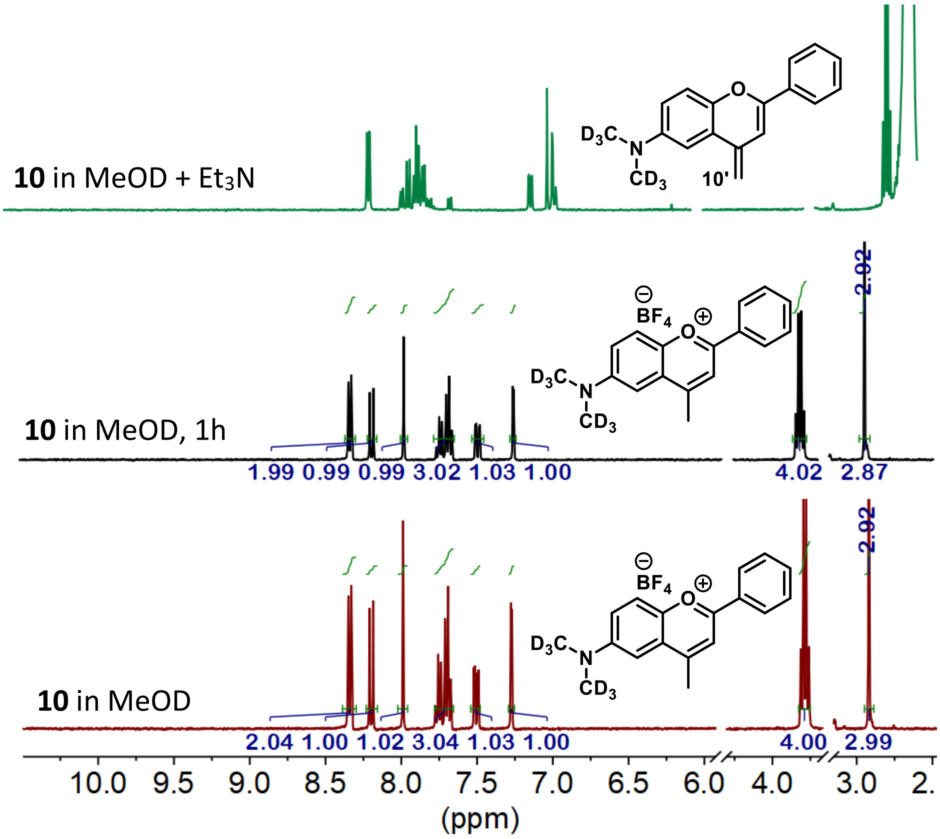

Figure S58. ¹H NMR spectrum of heterocycle **10** collected immediately after dissolving (red), after 1h of standing in MeOD (black), and after the addition of 3 µL of Et_3N.

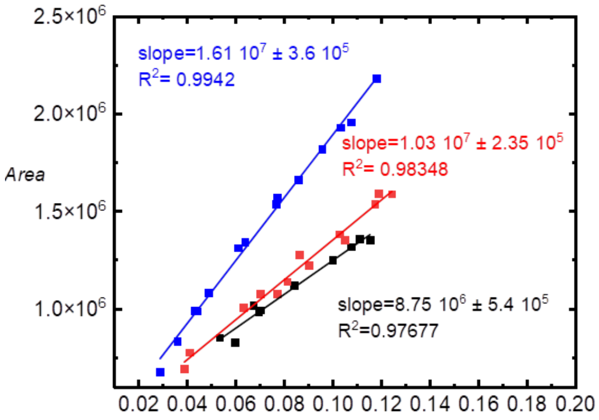

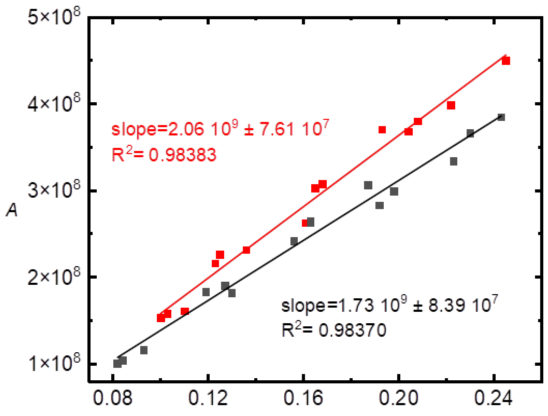

Figure S59. (left) The Φ_F data for **1H–DD** from Figure S51 and S53 plotted in a single graph. (right) The Φ_F data for **4H–D** from Figure S56 plotted in a single graph.

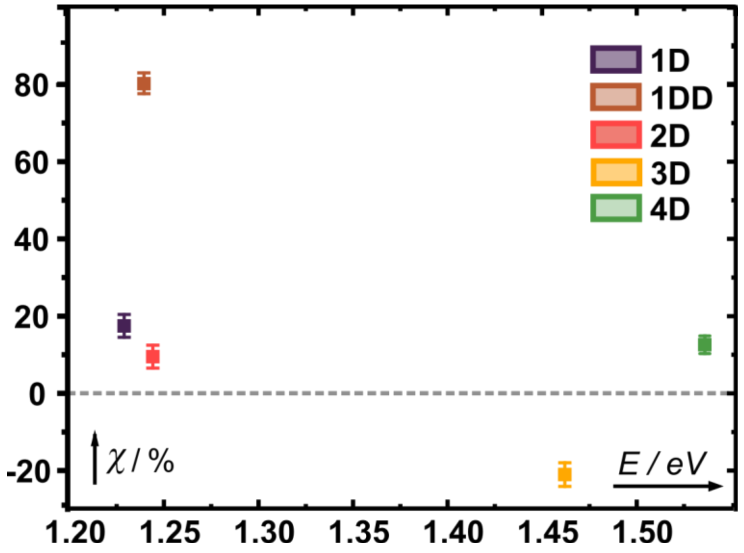

Figure S60. Enhancement (χ) of Φ_F in **1D–4D** as a function of the HOMO-LUMO gap.

Table S3. Statistical analysis of the relative Φ_F values.

compounds	*t*-value	*p*
**1H** vs **1D**	3.75	<0.10
**1H** vs **1DD**	14.64	<0.025
**1D** vs **1DD**	13.26	<0.025
**4H** vs **4D**	2.83	<0.15

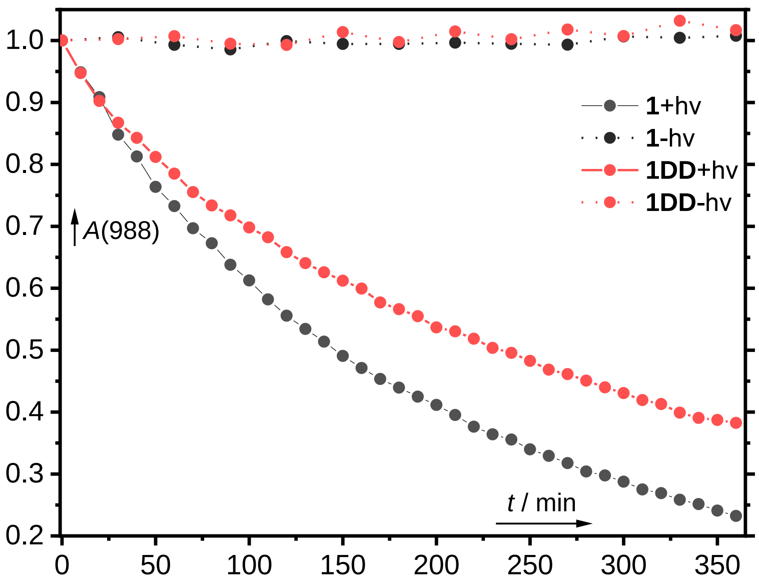

Figure S61. Comparison of stability of **1H** (red) and **1DD** (black) in DCM in the dark (dotted line) and under irradiation with light at 820 nm.

References

[1] Cosco E. D., Spearman A L., Shyam Ramakrishnan S., Lingg J. G. P., Saccomano M., Pengshung, M., Arús B. A., Wong K. C. Y, Glasl S., Ntziachristos V., Warmer M., McLaughlin R. R., Bruns O. T., Sletten E. M. *Nat. Chem.* **2020**, *12*, 1123–113.

[2] Tian M., Sun J., Dong B., Lin W. *Angew. Chem. Int. Ed.* **2018**, *57*,16506–16510.

[3] Li D., D. Smith B. D. *Chem. Eur. J.* **2021**, *27*, 14535– 14542.

[4] Jiang F., Hu Q., Zhang Z., Li H., Li H., Zhang D., Li H., Ma Y., Xu J., Chen H., Cui Y., Zhi Y., Zhang Y., Xu J., Zhu J., Lu T., Chen Y. *J. Med. Chem.* **2019**, *62*, 11080–11107.

[5] Sinha S. H., Owens E. A., Feng Y., Yang Y., Xie Y., Tu Y., Henary M., Zheng Y. G. *Eur. J. Med. Chem.* **2012**, *54*, 647–659.

[6] Cosco E. D., Caram J. R., Bruns O. T., Franke D., Day R. A., Farr E. P., Bawendi M. G., Sletten E. M. *Angew. Chem. Int. Ed.* **2017**, *56*, 13126–13129.

[7] Philip R., Penzkofer A., Bäumler W., Szeimies R. M., Abels C. J. Photochem. Photobiol., A **1996**, *96*, 137–148.
